# Localization, traffic and function of Rab34 in adipocyte lipid and endocrine functions

**DOI:** 10.1186/s12929-023-00990-8

**Published:** 2024-01-05

**Authors:** Jaime López-Alcalá, Ana Gordon, Andrés Trávez, Carmen Tercero-Alcázar, Alejandro Correa-Sáez, María Jesús González-Rellán, Oriol A. Rangel-Zúñiga, Amaia Rodríguez, Antonio Membrives, Gema Frühbeck, Rubén Nogueiras, Marco A. Calzado, Rocío Guzmán-Ruiz, María M. Malagón

**Affiliations:** 1 Department of Cell Biology, Physiology, and Immunology, Maimonides Institute for Biomedical Research of Córdoba (IMIBIC), University of Córdoba (UCO), Reina Sofía University Hospital (HURS), Córdoba, Spain; 2 CIBER Physiopathology of Obesity and Nutrition (CIBERobn), ISCIII, Madrid, Spain; 3 Department of Physiology, CiMUS, University of Santiago de Compostela-Instituto de Investigación Sanitaria, Santiago de Compostela, Spain; 4 Lipids and Atherosclerosis Unit, IMIBIC/University of Córdoba (UCO), Reina Sofía University Hospital (HURS), Córdoba, Spain; 5 Metabolic Research Laboratory, Department of Endocrinology & Nutrition, Clinic, University of Navarra, IdiSNA, Pamplona, Spain; 6 Department of Medical-Surgical Specialties, University of Córdoba (UCO), Reina Sofia University Hospital (HURS), Córdoba, Spain

**Keywords:** Rab34, Adipocytes, Golgi apparatus, Lipid droplets, Protein trafficking, Adiponectin, Lipid metabolism

## Abstract

**Background:**

Excessive lipid accumulation in the adipose tissue in obesity alters the endocrine and energy storage functions of adipocytes. Adipocyte lipid droplets represent key organelles coordinating lipid storage and mobilization in these cells. Recently, we identified the small GTPase, Rab34, in the lipid droplet proteome of adipocytes. Herein, we have characterized the distribution, intracellular transport, and potential contribution of this GTPase to adipocyte physiology and its regulation in obesity.

**Methods:**

3T3-L1 and human primary preadipocytes were differentiated in vitro and Rab34 distribution and trafficking were analyzed using markers of cellular compartments. 3T3-L1 adipocytes were transfected with expression vectors and/or Rab34 siRNA and assessed for secretory activity, lipid accumulation and expression of proteins regulating lipid metabolism. Proteomic and protein interaction analyses were employed for the identification of the Rab34 interactome. These studies were combined with functional analysis to unveil the role played by the GTPase in adipocytes, with a focus on the actions conveyed by Rab34 interacting proteins. Finally, Rab34 regulation in response to obesity was also evaluated.

**Results:**

Our results show that Rab34 localizes at the Golgi apparatus in preadipocytes. During lipid droplet biogenesis, Rab34 translocates from the Golgi to endoplasmic reticulum-related compartments and then reaches the surface of adipocyte lipid droplets. Rab34 exerts distinct functions related to its intracellular location. Thus, at the Golgi, Rab34 regulates cisternae integrity as well as adiponectin trafficking and oligomerization. At the lipid droplets, this GTPase controls lipid accumulation and lipolysis through its interaction with the E1-ubiquitin ligase, UBA1, which induces the ubiquitination and proteasomal degradation of the fatty acid transporter and member of Rab34 interactome, FABP5. Finally, Rab34 levels in the adipose tissue and adipocytes are regulated in response to obesity and related pathogenic insults (i.e., fibrosis).

**Conclusions:**

Rab34 plays relevant roles during adipocyte differentiation, including from the regulation of the oligomerization (i.e., biological activity) and secretion of a major adipokine with insulin-sensitizing actions, adiponectin, to lipid storage and mobilization from lipid droplets. Rab34 dysregulation in obesity may contribute to the altered adipokine secretion and lipid metabolism that characterize adipocyte dysfunction in conditions of excess adiposity.

**Supplementary Information:**

The online version contains supplementary material available at 10.1186/s12929-023-00990-8.

## Background

White adipose tissue (WAT) constitutes a highly dynamic, metabolically active organ, that stores energy excess and also serves as an important endocrine organ [1, 2]. The mature cells of WAT, the adipocytes, store fatty acids from the diet in form of triacylglycerols (TGs) (i.e., lipogenesis) in lipid droplets (LDs) [3, 4]. In conditions of energy demand, TGs are hydrolyzed (lipolysis) to fatty acids and glycerol, which are then used as fuel by other tissues [5]. These fine-tuned processes of lipid storage and mobilization in adipocytes are essential for whole-body energy homeostasis and therefore, they rely on complex regulatory mechanisms involving many molecular components [6, 7].

Many of these regulatory mechanisms occur at the LDs, which represent the integrating center of lipid metabolism in adipocytes. LDs also participate in protein degradation [8], and serve as platforms for cell defense against pathogens [9, 10]. LDs are composed of a TG core surrounded by a phospholipid monolayer that is associated with a large variety of proteins [3, 11]. Besides lipases and enzymes involved in TG synthesis, the LD coat also comprises various members of perilipin (PLIN) and cell death-inducing DFFA-like effector (CIDE) families, which regulate LD formation, growth, and maintenance [3, 12, 13]. The LD surface is also decorated with proteins involved in intracellular trafficking processes and/or in the interaction of LDs with other organelles, such as Rab and ADP-Ribosylation Factor (ARF) GTPases, and soluble N-ethylmaleimide-sensitive factor attachment proteins receptors (SNAREs) [3, 12, 13]. More recently, LDs have been shown to associate with components of the ubiquitin–proteasome system to maintain LD protein turnover, and to serve as molecular folding platforms [13–17]. Despite the knowledge gained on LD protein coat composition, the mechanisms and intracellular routes by which proteins target the LD surface are still not fully understood [18].

Among the most represented proteins in the LD coat is the Rab family of small GTPases [19, 20]. Rab proteins act as molecular switches that, when activated, associate with intracellular membranes and recruit specific effector proteins that translate Rab actions on target membranes [20, 21]. Some LD-associated Rab proteins play key roles in lipid storage and mobilization [20]. This is the case of the best characterized Rab protein in adipocytes, Rab18, which interacts with SNARE complexes on the endoplasmic reticulum (ER) to regulate LD biogenesis in adipocyte precursors, namely the preadipocytes [22–24]. Moreover, studies from our lab have shown that Rab18 promotes LD-ER interactions and participates in both insulin-mediated lipogenesis and β-adrenergic-induced lipolysis in adipocytes [24]. These results, together with the increased expression levels of Rab18 found in adipocytes from individuals with obesity support a role for this GTPase in the regulation of lipid homeostasis under both physiological and pathological conditions [23, 24]. In this line, dysfunctional Rab signaling due to changes in Rabs or Rab-interacting proteins has been associated with various diseases such as cancer and neurodegenerative diseases [25].

Our group recently discovered that Rab34, a member of the Rab family, is present in the LD protein cover in adipocytes [19]. However, its function in these cells remains unknown. In this study, our objective was to investigate the trafficking, mechanisms of action, and impact of Rab34 on adipocyte differentiation (i.e., adipogenesis) and function, as well as to unveil the role of this GTPase in lipid turnover.

## Methods

### Cell culture

3T3‐L1 cells [American Type Culture Collection (ATCC), USA] were differentiated into adipocytes according to our standard protocols [24, 26]. Briefly, cells were seeded onto 6-well/15 cm diameter plates or glass coverslips at a density of 1800 cells/cm^2^ and differentiated in Dulbecco’s modified Eagle’s medium (DMEM) supplemented with 10% v/v fetal bovine serum (FBS), 0.5 mmol/L 3-isobutyl-1-methylxanthine, 0.25 mmol/L dexamethasone, and 10 μg/mL insulin for 3 days. Thereafter, the medium was replaced by DMEM with 10% v/v FBS and 10 mg/mL insulin and incubated for an additional 3-day period. Then medium was replaced with fresh DMEM containing 10% v/v FBS every 3 days until day 10 (D10). Cells and culture medium were collected on different days of differentiation and/or after the experimental treatments. For experiments using three-dimensional (3D) cell cultures, 3T3-L1 cells were cultured and differentiated in 3D-based collagen type I (COL-I) microgels using our established protocols [19].

Human primary adipocytes (mature adipocytes and preadipocytes, either freshly isolated or differentiated in vitro) were obtained from subcutaneous (SC) and omental (OM) adipose tissue samples of individuals with obesity [Body Mass Index (BMI) > 40 kg/m^2^] undergoing bariatric surgery (see “Declarations” section) following our standard protocols [27]. Patients underwent a clinical assessment including medical history, physical examination, and body composition analysis (Additional file 1). Biochemical assays were carried out as previously described [27, 28]. Briefly, following adipose tissue digestion using 2 mg/mL collagenase (Sigma-Aldrich, Madrid, Spain), the resulting mixture was filtered through cell strainer filters with a 100 µm pore size to eliminate undispersed tissue, and mature adipocytes were isolated as described [27]. After washing, a mixture of 35 μL of mature adipocytes and 35 μL of Matrigel (BD Biosciences, Bedford, USA) was prepared and quickly placed on a coverslip in a 35 mm diameter plate. The mixture was carefully spread on the surface of the coverslip and incubated for 30 min at 37 ºC. Then, 1 mL of dispersion medium was added to the plate, which was incubated for 1 h at 37 ºC, and washed with phosphate-buffered saline (PBS) before the mature adipocytes were fixed with 4% w/v paraformaldehyde (PFA) (15 min). Regarding the preadipocytes, they were obtained and differentiated in vitro as previously described [27]. Briefly, cells of the stromal-vascular fraction (SVF) were seeded in preadipocyte-proliferation medium DMEM/F-12 (1:1) supplemented with 8 mmol/L biotin, 18 mmol/L d-pantothenate acid, 100 mmol/L ascorbate, 1% v/v penicillin–streptomycin, and 10% v/v new-born calf serum (NCS) at 37 ºC in a humidified atmosphere with 95% air: 5% CO_2_. Medium was replaced every 48 h until confluency. Thereafter, cells were detached with trypsin–EDTA solution and cultured at 4000 cells/cm^2^ three times to purify and amplify the cell culture following our established methods [27]. Preadipocytes were seeded onto glass coverslips at a density of 4000 cells/cm^2^ and induced for adipogenic differentiation. Cells were fixed with 4% w/v PFA (15 min) at D5 and D10 of differentiation. Immunostaining of preadipocytes and mature adipocytes was carried out using our standard protocols (see “Immunocytochemistry and Confocal Microscopy” section).

HEK-293 AD cells (ATCC) were cultured in DMEM medium containing 1 g/L glucose and supplemented with 2 mmol/L L-glutamine, 0.25 μg/mL gentamicin/amphotericin solution and 10% v/v FBS, and kept at 37 ºC and 5% CO_2_. For co-immunoprecipitation studies, cells were seeded on 15 cm plates at a density of 6000 cells/cm^2^, and transfected at 60% confluency.

### Animal models

Male wild-type (WT) and leptin-deficient (ob/ob) mice (C57/BL/6 J; 8-week-old) were used for the experiments. All animals were housed in individual cages under controlled conditions of illumination (12 h light/dark cycle), temperature and humidity. The animals were allowed free access to water and a standard laboratory diet (STD) (Scientific Animal Food & Engineering, proteins 16%, carbohydrates 60% and fat 3%) or high fat diet (HFD) (Research Diets 12,492; 60% of calories from fat, 5.24 kcal/g; Research Diets, New Brunswick, NJ) for 12 weeks [29]. The animals were euthanatized, and all the tissues were removed rapidly, frozen immediately on dry ice, and kept at − 80 ºC until analysis.

### Immunocytochemistry and confocal microscopy

3T3‐L1 cells or human (pre)adipocytes were fixed in 4% w/v PFA (15 min), incubated with PBS containing 0.3% w/v saponin and 1% w/v bovine serum albumin (1 h at RT), and then exposed to the corresponding primary antibodies (Additional file 2). Thereafter, Alexa Fluor™ 594‐conjugated secondary antibody, alone or in combination with Alexa Fluor™ 488- and/or an Alexa Fluor™ 405‐conjugated secondary antibody (Additional file 2) were employed. In some experiments, cells were counterstained with Oil Red O [for LD quantification] (Sigma-Aldrich) or ER-Tracker [for ER quantification] (Invitrogen, Carlsbad, CA, USA). Samples were mounted on slides with Dako Fluorescence Mounting Medium (Agilent Technologies, Madrid, Spain) with or without 1 μg/mL 4’, 6-diamidino-2-phenylindole (DAPI) (Sigma-Aldrich) to visualize the nuclei and examined under a ZEISS LSM710 confocal laser scanning microscope (Carl Zeiss AG., Oberkochen, Germany). Confocal images were processed using the Huygens Essential software package (SVI, Hilversum, Netherlands). Colocalization of the fluorescence signals was estimated by determining the overlapping pixel map of the channels (i.e., mask) using the Colocalization Finder plugin for Fiji/ImageJ (NIH), and the Manders’ coefficient using the Colocalization Threshold plugin for the same software. Negative controls without primary or secondary antibodies were included to assess nonspecific staining.

### Plasmid expression vectors

Plasmids coding for GFP (pEGFP-N1) and ubiquitin (pHA-ubiquitin) were kindly supplied by Dr. M.A. Calzado (IMIBIC, Córdoba, Spain) [30, 31]. Plasmids coding for wild-type Rab34 (pEGFP-Rab34-WT), constitutively active Rab34 (pEGFP-Rab34-Q111L), and constitutively inactive Rab34 (pEGFP-Rab34-T66N) were kindly supplied by Dr. T. Wang (Membrane Biology Laboratory, Institute of Molecular and Cell Biology, Singapore, Singapore) [32]. Plasmids coding for Adipose Triglyceride Lipase (ATGL) (pGFP-ATGL), Hormone-Sensitive Lipase (HSL) (pGFP-HSL) and Perilipin (pGFP-PLIN1) were kindly supplied by Dr. T. Osumi (Graduate School of Life Science, University of Hyogo, Hyogo, Japan) [33]. Plasmids coding for Fatty Acid Binding Protein 4 (FABP4) (pFABP4-GFP) and 5 (FABP5) (pFABP5-EGFP) were kindly supplied by Dr. N. Noy (Case Western Reserve University, USA) and Dr. M. Kaczocha (Stony Brook University, USA), respectively [34, 35]. Plasmid coding for biotin ligase BirA (pcDNA3.1mycBioID) was purchased from Addgene (Cambridge, USA). Plasmid coding for E1 Ubiquitin-Activating Enzyme 1 (UBA1) (pCMV3-UBA1-c-Myc) was purchased from SiNo Biological (Düsseldorfer, Eschborn, Germany). Plasmids coding for Ubiquitin C-Terminal Hydrolase L3 (UCHL3) (pCMV6-UCHL3-c-Myc) and ISG15 Ubiquitin Like Modifier (ISG15) (pCMV6-ISG15-c-Myc) were purchased from OriGene (Rockville, USA). Other plasmids coding for Rab34 (pKate-Rab34-WT and pcDNA3.1mycBioID*Rab34) were prepared for this work following our established protocols [26].

### Expression and silencing studies

For expression assays, 3T3‐L1 cells, human adipocytes, and HEK-293 AD cells, were transfected with the corresponding plasmid vectors at 2.5 µg/mL using a 7.5:1000 dilution of Lipofectamine 2000 (Invitrogen), and cultured for 48 h prior to the experiments. For silencing studies, cells were transfected with a 7.5:1000 dilution of Lipofetamine RNAiMAX (Invitrogen) and mouse Rab34 siRNA (Dharmacon), mouse UBA1 siRNA (Dharmacon), or control siRNA (Sigma-Aldrich) (scrambled-transfected cells) at 25 nmol/L. Then, cells were kept in culture for 72 h. At the end of the experiments, cells were processed for confocal microscopy and/or immunoblotting as indicated in the corresponding sections. In another set of experiments, cells were collected in radioimmunoprecipitation assay (RIPA) buffer and intracellular concentration of TGs was determined using Triglyceride Reagent (Sigma-Aldrich) and Amplex UltraRed Reagent (Invitrogen), while culture media were analyzed for free glycerol content using Amplex UltraRed Reagent (Invitrogen) and Free Glycerol Reagent (Sigma-Aldrich) as previously described [24].

### Quantification of adiponectin secretion

Quantification by ELISA of intra- and extracellular adiponectin levels in 3T3-L1 cells expressing GFP-Rab34 or silenced for this protein by Rab34 siRNA treatment was carried out using Quantikine ELISA Mouse Adiponectin/Acrp30 Immunoassay (R&D Systems Europe, Abingdon, UK) using manufacturer’s instructions.

### Experimental treatments

In order to unveil Rab34 traffic route(s) during adipocyte differentiation, 3T3‐L1 adipocytes at D2 and D4 of differentiation were preincubated in serum‐free culture medium (2 h) and then cultured in the absence or presence of brefeldin A (BFA) (Sigma-Aldrich) (20 µmol/L) for 1 h. Thereafter, medium was removed and cells were maintained in fresh culture medium for 48 h, when they were processed for confocal microscopy.

In another set of experiments, 3T3-L1 cells at D5 (for expression studies) or D6 (for silencing studies) of differentiation were exposed to MG132 (Sigma-Aldrich) (10 µmol/L, 12 h) for protein turnover studies. Cells were collected and processed for immunoblotting studies and/or analysis of lipogenesis/lipolysis.

### Immunoblotting

Protein extracts were obtained from cells lysed in RIPA buffer containing 50 mmol/L Tris‐HCl (pH 7.4), 150 mmol/L NaCl, 1% v/v Triton‐X‐100, 1 mmol/L EDTA and 1 μg/mL protease and phosphatase inhibitor cocktail (Thermo Fisher Scientific, Barcelona, Spain). Extracts were resuspended in loading buffer 5X (500 mmol/L Tris–HCl, 7.5% w/v SDS, 10 mmol/L EDTA, 50% w/v sucrose, 5% v/v β-mercaptoethanol, 250 mmol/L DTT, and 5 mg/mL bromophenol blue, pH 6.8) and heated at 97 ºC for 5 min. For detection of adiponectin oligomers, cell lysate samples or proteins precipitated by acetone‐methanol from the culture media [19] were mixed with non-reducing loading buffer (without β-mercaptoethanol) containing 500 mmol/L Tris–HCl, 7.5% w/v SDS, 10 mmol/L EDTA, 50% w/v sucrose, and 5 mg/mL bromophenol blue, pH 6.8.

Samples (20–30 µg) were separated by SDS-PAGE under denaturing conditions and transferred to nitrocellulose membranes (Bio-Rad Laboratories, Inc.) as described previously [26, 27]. Primary antibodies (Additional file 2) were dispensed overnight (4 ºC) and peroxidase‐conjugated secondary antibodies (Additional file 2) were incubated for 1 h at RT. The immunoreaction was visualized using Clarity Western ECL Substrate (BioRad, Hercules). β-Actin or Ponceau S [28] were selected as loading controls. Densitometric analysis of the immunoreactive bands was carried out with Fiji/ImageJ (NIH) software. The immunoblots shown in the manuscript included 3–4 biological replicates corresponding to the complete set of samples (control and experimental groups) that were run on the same gel, unless otherwise indicated in the corresponding figure legend. The original uncropped blots of specific Figures are provided as supplementary materials (Additional file 3: Figs. S6–S21).

### Identification of Rab34 interactome

In order to identify the interacting protein partners of Rab34, two different techniques were employed: i) Proximity-dependent biotin identification (BioID) studies and, ii) Immunoprecipitation of protein extracts from 3T3-L1 cells expressing GFP-Rab34 followed by Tandem Mass Spectrometry (MS/MS) analysis.

#### Proximity-dependent biotin identification (BioID) studies

Proximity-dependent biotin identification (BioID) has been widely used for the identification of candidate protein–protein interactions [36]. For studies on Rab34, 3T3-L1 cells were transfected at D6 with the plasmid vectors c-Myc-BirA (mock) or c-Myc-BirA-Rab34. After 24 h, cells were incubated with complete medium supplemented with 1 µg/mL doxycycline and 50 µmol/L biotin for another 24 h. Cells were then lysed at 25 ºC in 1 mL lysis buffer [50 mmol/L Tris (pH 7.4), 500 mmol/L NaCl, 0.4% w/v SDS, 5 mmol/L EDTA, 1 mmol/L DTT, and 1 μg/mL protease and phosphatase inhibitor cocktail (Thermo Fisher Scientific)] and then sonicated with 3 pulses of 10 s, in the presence of 2% v/v Triton X-100. Subsequently, an equal volume of 50 mmol/L Tris (pH 7.4, 4 ºC) was added and sonicated again. Cells were centrifuged at 16,000 g (4 ºC) and the supernatant (6 mg protein) was incubated with 6 mg Dynabeads [MyOne Streptavadin C1 Dynabeads (Invitrogen)] overnight (4 ºC). Beads were collected and washed in 1 mL wash buffer 1 (2% w/v SDS in H_2_O) and then, in wash buffer 2 (0.1% w/v sodium deoxycholate, 1% v/v Triton X-100, 500 mmol/L NaCl, 1 mmol/L EDTA, and 50 mmol/L HEPES, pH 7.5, 25 ºC), wash buffer 3 (250 mmol/L LiCl, 0.5% v/v NP-40, 0.5% w/v sodium deoxycholate, 1 mmol/L EDTA, and 10 mmol/L Tris, pH 8.1, 25 ºC), wash buffer 4 (50 mmol/L Tris, pH 7.4 and 50 mmol/L NaCl, 25 ºC), and finally, in 50 mmol/L NH_4_HCO_3_ (25 ºC). Thereafter, beads were exposed to 20 mmol/L DTT in vertical shaking (30 min, 37 ºC) to reduce proteins. Then, iodoacetamide was added to a final concentration of 20 mmol/L and beads were incubated (40 min, 37 ºC) in the dark and vertical shaking for alkylation. Finally, mass spectrometry grade trypsin (Promega, New South Wales, Australia) was added in a 1:50 ratio for overnight digestion at 37 ºC. Beads were separated by magnetic attraction, and formic acid (Scharlab, Barcelona, Spain) was added to the peptide solution to a concentration of 2% v/v, before MS/MS analysis.

Digested peptides (4 µg of sample) of each sample were separated using Reverse Phase Chromatography. Data acquisition was carried out in a Triple quadrupole Time-Of-Flight (TripleTOF) 6600 System (Ab Sciex, Madrid, Spain) using a Data dependent workflow. After MS/MS analysis, data files were processed using ProteinPilot 5.0.1 software (Ab Sciex). False discovery rate (FDR) was determined using a non-linear fitting method and only those results with FDR < 1% were selected. Only proteins that were absent in mock-transfected cells were considered potential interaction partners. Endogenously biotinylated mammalian carboxylases, nuclear histones and usual contaminants (keratins, IgGs and proteolytic enzymes) were also removed [36].

#### Immunoprecipitation and MS/MS studies

3T3-L1 cells were transfected at D6 with GFP-Rab34 or mock plasmid vector and immunoprecipitation studies were carried out as described [26, 31]. Briefly, cells were lysed 48 h after transfection with lysis buffer [50 mmol/L Tris–HCl (pH 7.4), 150 mmol/L NaCl, 5 mmol/L EDTA, 1% Triton X-100, 1% protease inhibitor cocktail]. After incubation with anti-GFP antibody or anti-IgG (control) overnight at 4 °C, immune complexes were captured by mixing with protein G-Sepharose beads (GE Healthcare Bio-Sciences, Madrid, Spain) for 2 h at RT. After washing with washing buffer [(20 mmol/L Tris–HCl (pH 7.5), 150 mmol/L NaCl, 1 mmol/L EDTA, 1% v/v Triton-X100 and 1% phosphatase inhibitor cocktail], samples were eluted and the supernatants resuspended in 150 µL buffer containing 50 mmol/L Tris‐HCl (pH 7.4), 150 mmol/L NaCl, 1% v/v Triton‐X‐100, 1 mmol/L EDTA, and 1 μg/mL protease and phosphatase inhibitor cocktail.

Proteins in the samples were extracted using methanol-chloroform and protein pellets were reconstituted in 40 µL of RapiGest (Waters Cromatografía, Barcelona, Spain). Protein content was assessed by microfluorimetry using Qubit Protein Assay (Thermo Fisher Scientific) and proteins in the samples were double-digested with advanced iST (in-Stage-Tip kit) using Trypsin and LysC as enzymes, according to manufacturer’s instructions (Preomics, Munich, Germany). Samples (100 ng) were loaded onto Evotip Pure (Evosep Biosystems, Odense, Denmark) and then analyzed by Liquid Chromatography (LC)-MS/MS (DIAmode/EvosepOne/TIMSTOF-Flex). Proteins were subsequently identified and quantified using DIA-NN software [37].

### Co-immunoprecipitation assays

In order to determine the interaction between Rab34 and the selected proteins, either due to their altered levels or because they were identified by both BioID and immunoprecipitation studies (ATGL, HSL, FABP4, FABP5, ISG15, PLIN1, UBA1, and UCHL3), we employed a standard protocol for co-immunoprecipitation studies in HEK‐293 AD cells transfected with the corresponding expression vectors [26, 31]. We also performed co-immunoprecipitation studies in HEK‐293 AD cells transfected with wild-type Rab34 (pEGFP-Rab34-WT), constitutively active Rab34 (pEGFP-Rab34-Q111L), or constitutively inactive Rab34 (pEGFP-Rab34-T66N) and FABP5 (pFABP5-EGFP). Details of the antibodies used for pulling down and/or detecting target proteins by Western blot are listed in Additional file 2.

### RNA extraction and real-time PCR

RNA isolation and purification from 3T3-L1 cells were performed as described [19]. Specific primers for Rab34 (mouse, forward primer 5′-AAGGTCATCGTTGTGGGAGA-3′, and reverse primer 5′-GTTGGAGACTGAAGGGGACA-3′), FABP5 (mouse, forward primer 5′-CGAGAGCACAGTGAACACGA-3′, and reverse primer 5′-CCATTGCTGGTGCTGGA-3′), Fatty Acid Synthase (FASN) (mouse, forward primer 5′-ATACAATGGCACCCTGAACC-3′, and reverse primer 5′-TTACAGAGGAGAAGGCCACAA-3′), Acyl-CoA Synthetase Long Chain Family Member 1 (ACSL1) (mouse, forward primer 5′-AGCAGTTCATCGGCCTCTT-3′, and reverse primer 5′-GTTTGGCTTTTTCTGGCTTG-3′) and Glycerol-3-Phosphate Acyltransferase (GPAT) (mouse, forward primer 5′-TTATCACCAGGACGGAAAGG-3′, and reverse primer 5′-TCTCTTTGAAAACCCCGATG-3′) were employed for RT-PCR studies using our established protocols [19]. Specific signals were normalized with the constitutively expressed gene, hypoxanthine–guanine phosphoribosyltransferase (HPRT) [19]. All samples were run in duplicates and the average values were calculated.

### Statistical analysis

Statistical analysis was carried out using GraphPad Prism 8 statistical software, (GraphPad Software). Prior to each analysis, outliers were identified and eliminated using the ROUT method (Q = 1%). The normal distribution of variables was assessed using the Shapiro–Wilk’s test. One/Two‐Way ANOVA, Kruskal–Wallis’s test, Independent/Multiple‐Samples *t*-test or Mann–Whitney’s test were used where appropriate. A post‐hoc statistical analysis using Tukey’s test was performed to identify significant differences between groups. Values were considered significant at P < 0.05. For confocal imaging assays, at least 6 micrographs from two individual experiments were analyzed. For the remaining assays, at least three individual experiments were analyzed.

## Results

### Intracellular distribution of Rab34 in preadipocytes and adipocytes

**Fig. 1:**
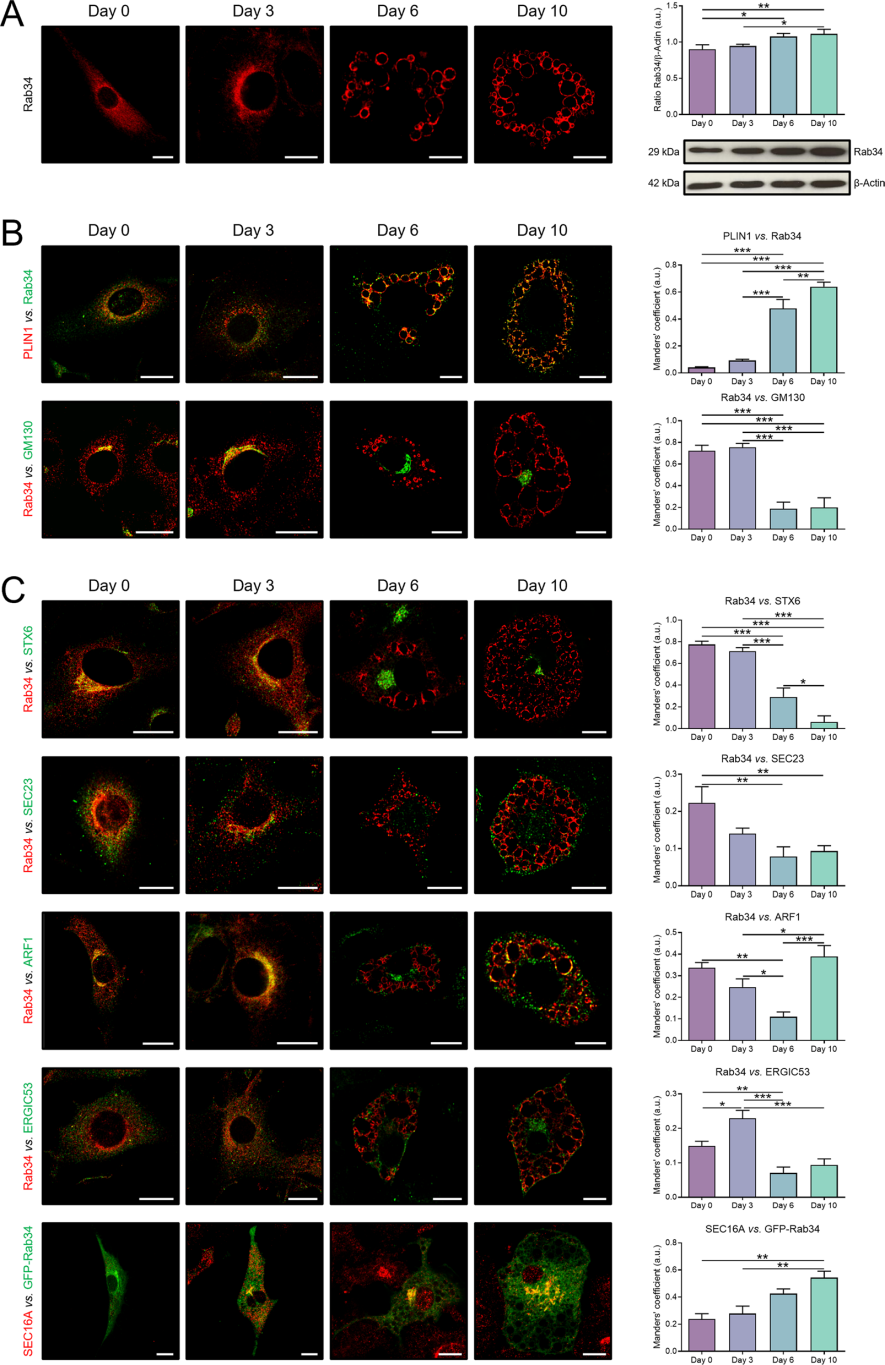
Rab34 distribution and expression during differentiation of 3T3-L1 cells into adipocytes. **A** Representative confocal images of 3T3-L1 cells immunostained for Rab34 during differentiation (days 0, 3, 6 and 10), and representative immunoblot and protein quantification of Rab34 in 3T3-L1 cell extracts during differentiation (days 0, 3, 6 and 10). Data are expressed as the ratio of Rab34 immunosignal to β-Actin immunosignal and represent the mean ± SEM of n = 4 biological replicates. **B** Representative images of 3T3-L1 cells double-immunostained with antibodies against Rab34 and the LD marker, PLIN1, or the cis-Golgi marker, GM130, respectively. **C** Colocalization study of Rab34 with cell compartments during the process of differentiation of 3T3-L1 cells. 3T3-L1 cells were labeled with anti-Rab34 or GFP-Rab34 and antibodies against different intracellular markers: STX6 (trans-Golgi), SEC23 (COPII vesicles), ARF1 (COPI vesicles), ERGIC53 (ERGIC), or SEC16A (ERES). Manders’ coefficients were calculated to assess the colocalization between signals. Data are expressed as the mean ± SEM (n = 12 cells/differentiation day, 2 replicate studies). *P < 0.05; **P < 0.01; ***P < 0.001. Scale bar: 10 μm

We recently identified Rab34 as a component of the LD proteome in differentiated 3T3-L1 adipocytes [19]. In order to study in detail its role during adipocyte differentiation, we analyzed Rab34 expression by confocal microscopy in 3T3-L1 cells. As shown in Fig. 1A, we observed a clear association of Rab34 with the LD surface from D3 of differentiation onwards, concurrent with the appearance of LDs in the cytoplasm of differentiating preadipocytes [38]. A progressive increase in the amount of Rab34 throughout the differentiation process (D0-D10) was also observed (Fig. 1A). Colocalization of Rab34 with the LD surface marker PLIN1 increased by 11- and fivefold at D6 as compared to D0 and D3, respectively, and reached its maximum value at D10 (Fig. 1B). At early stages of adipocyte differentiation (D0-D3), i.e., when the cells still display a fibroblast-like appearance and are devoid of LDs, Rab34 accumulated in juxtanuclear structures reminiscent of the Golgi apparatus (Fig. 1A). Double-immunolabeling experiments utilizing an antibody against the cis-Golgi matrix marker, GM130, confirmed this observation. Quantitative assessment of the colocalization rate between Rab34 and GM130 immunosignals revealed an opposite pattern to that of Rab34-PLIN1 (Fig. 1B).

To validate the results, we examined the distribution of GFP-Rab34, which displayed a distribution comparable to that of endogenous Rab34 at both early and late stages of differentiation, with some labeling persisting at the Golgi in differentiated cells (Fig. 1C). In fact, a clear overlap between endogenous and exogenous Rab34 signals could be observed (Additional file 3: Fig. S1A). Additionally, we employed two different Rab34 mutant vectors: i) pEGFP-Rab34-Q111L, and ii) pEGFP-Rab34-T66N [32]. It has been shown that only the wild-type and the GTP-restricted Q111L mutant were capable of binding GTP, whereas the GDP-restricted form failed to interact with GTP [32]. Interestingly, the intracellular distribution of the constitutively active variant of Rab34, GFP-Rab34-Q111L, was similar to the WT version (Additional file 3: Fig. S1B). In contrast, slightly lower signal intensity was observed for the constitutively inactive variant, GFP-Rab34-T66N, which was restricted to the Golgi apparatus (Additional file 3: Fig. S1C). The differences in intracellular distribution between the WT version and the constitutively active (GFP-Rab34-Q111L) *vs*. the constitutively inactive variant (GFP-Rab34-T66N) are consistent with previous reports and have been described in other cell types [32, 39–41]. In summary, these results demonstrate that Rab34 exhibits a distinct pattern of intracellular localization throughout the process of adipocyte differentiation.

### Rab34 as a model of protein traffic to LDs during adipocyte differentiation

The mechanisms by which proteins target the LD coat represent an active research area [18]. In this line, we took advantage of the stage-dependent dual localization of Rab34 during adipocyte differentiation to explore how this GTPase reaches the LD surface. First, we carried out double labeling studies in 3T3-L1 cells throughout differentiation (D0-D10) using the anti-Rab34 antibody (or GFP-Rab34 when appropriate antisera for double immunolabeling experiments were not available) in combination with location markers of different intracellular compartments. The results showed that Rab34 localized to the Golgi apparatus, from the *cis* side to the *trans*-Golgi network (TGN), at early stages of adipocyte differentiation (Fig. 1C). As observed for GM130, colocalization rates of Rab34 and the TGN marker, syntaxin-6 (STX6), were significantly reduced from D6, reaching the lowest values in differentiated adipocytes (Fig. 1C). A similar trend was observed for the Coat protein complex II (COPII) marker, SEC23 (Fig. 1C). Notably, while colocalization of Rab34 with the Coat protein complex I (COPI) marker, ARF1, also decreased gradually from D0-D6, it significantly increased in differentiated adipocytes, i.e., when Rab34 was mostly associated with the LD surface (Fig. 1C). Similarly, it has been also proposed that the function of ARF1 in protein delivery to LDs occurs in the context of a close apposition of ER-Golgi Intermediate Compartment (ERGIC) and the ER Exit Sites (ERES) with LDs [42]. Accordingly, we also analyzed the colocalization rates between Rab34 and markers of these compartments, ERGIC53 and SEC16A, respectively. The findings indicate that a peak of colocalization between Rab34 and ERGIC53 immunosignals occurred at D3 and then decreased significantly toward the end of the differentiation process. In contrast, the Manders’ coefficient for SEC16A/GFP-Rab34 exhibited a gradual increase from D0 to D10 (Fig. 1C).

**Fig. 2:**
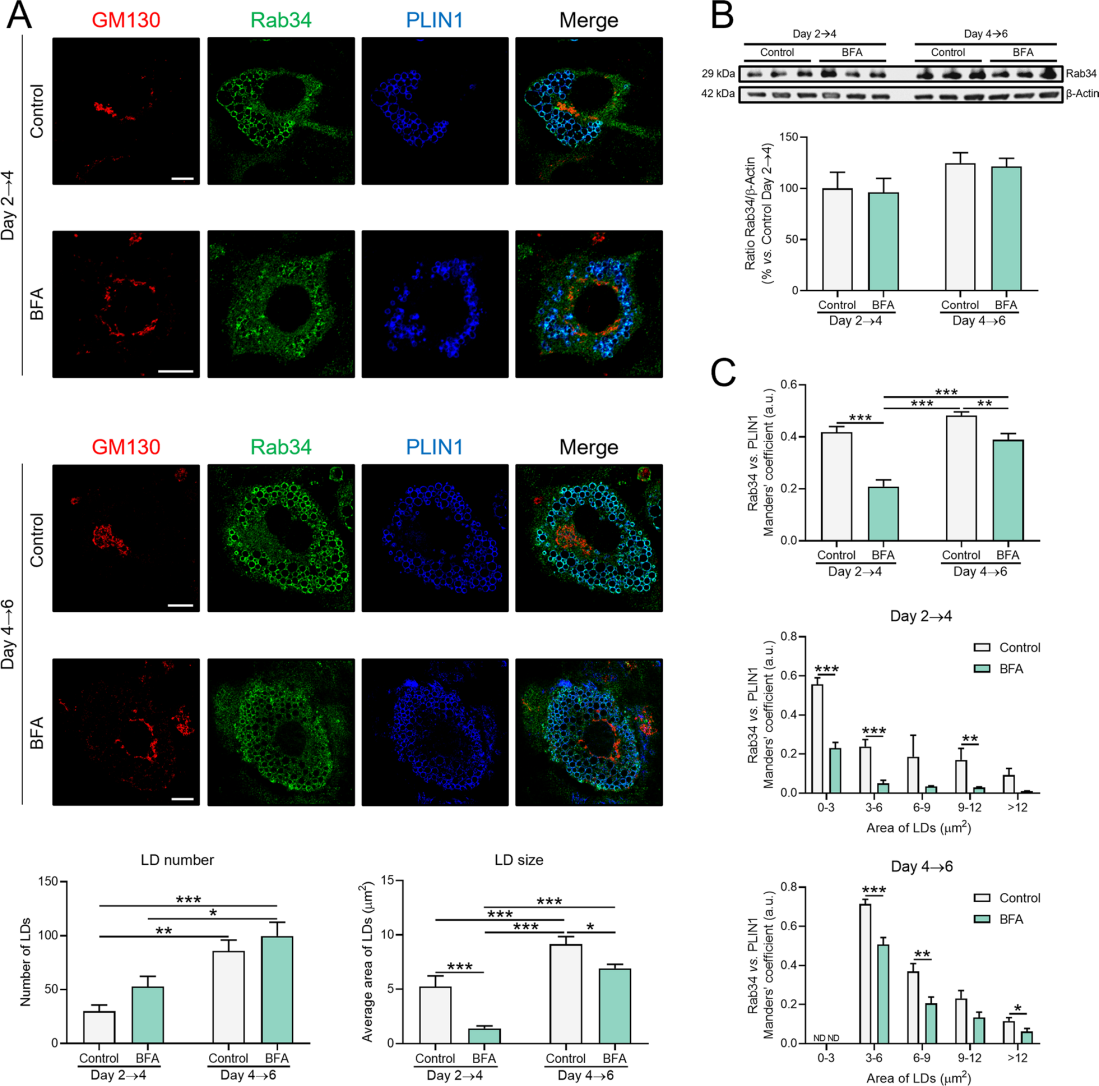
Analysis of Rab34 association with LDs upon brefeldin A (BFA) treatment. 3T3-L1 cells were exposed to BFA (20 μmol/L, 1 h) at day 2 or day 4 of differentiation. Forty-eight hours after treatment, cells were processed for confocal microscopy or immunoblotting studies. **A** Cells were triple-immunostained with anti-GM130, anti-Rab34 and anti-PLIN1 sera. Graphs show the number and average size of LDs (µm^2^) per cell and per experimental condition (n = 10 cells/experimental condition, 2 replicate studies). Scale bar: 10 μm. **B** Western blot analysis of Rab34 protein content in cells exposed to medium alone (control) or to BFA at D2 or D4. Data are referred to control cultures at day 2 of differentiation (100%) and expressed as mean ± SEM (n = 3 biological replicates/experimental condition). **C** Manders’ coefficient (expressed in arbitrary units, a.u.) for colocalization of Rab34 and PLIN1 immunosignals in 3T3-L1 cells, expressed as mean value per cell (upper graph) or according to LD area (µm^2^) (middle and lower graphs). Data are expressed as mean ± SEM (n = 10 cells/experimental condition, 2 replicate studies). *P < 0.05; **P < 0.01; ***P < 0.001

Finally, since the ARF1/COPI vesicular trafficking machinery is required for ER-to-LD targeting of several LD-associated proteins [14], we explored whether this system was also important for Rab34 traffic to LDs in adipocytes. To this end, 3T3-L1 cells were exposed to BFA, a reversible inhibitor of ER-to-LD trafficking [43], at early (D2) and later (D4) stages of differentiation. As expected, BFA treatment induced Golgi fragmentation (Fig. 2A). Although BFA treatment reduced the size of LDs, particularly when given at D2, no changes in LD number were observed (Fig. 2A). Protein expression studies indicated that Rab34 content was not altered by BFA at any of the time points tested (Fig. 2B). However, our microscopy studies revealed that BFA administration to 3T3-L1 cells at either D2 or D4 diminished the association of Rab34 with LDs, irrespective of LD size (Fig. 2C). Notably, the inhibitory effect of BFA on Rab34 binding to LDs was considerably higher when the drug was administered at early stages of differentiation (50% reduction in cells treated at D2 *vs.* 23% at D4 with respect to their corresponding controls) (Fig. 2C).

These data collectively support that Rab34 accumulates in the Golgi apparatus in preadipocytes. Our results also suggest that, when LD biogenesis is induced, the GTPase migrates to ERGIC or ERES, and this retrograde transport may involve BFA-sensitive pathways. During LD formation, Rab34 may reach the LD surface through the ER mainly via ERGIC, while the ERES could represent the main Rab34-targeting pathway from the ER to expanding/mature LDs.

### Role of Rab34 in Golgi apparatus organization

**Fig. 3:**
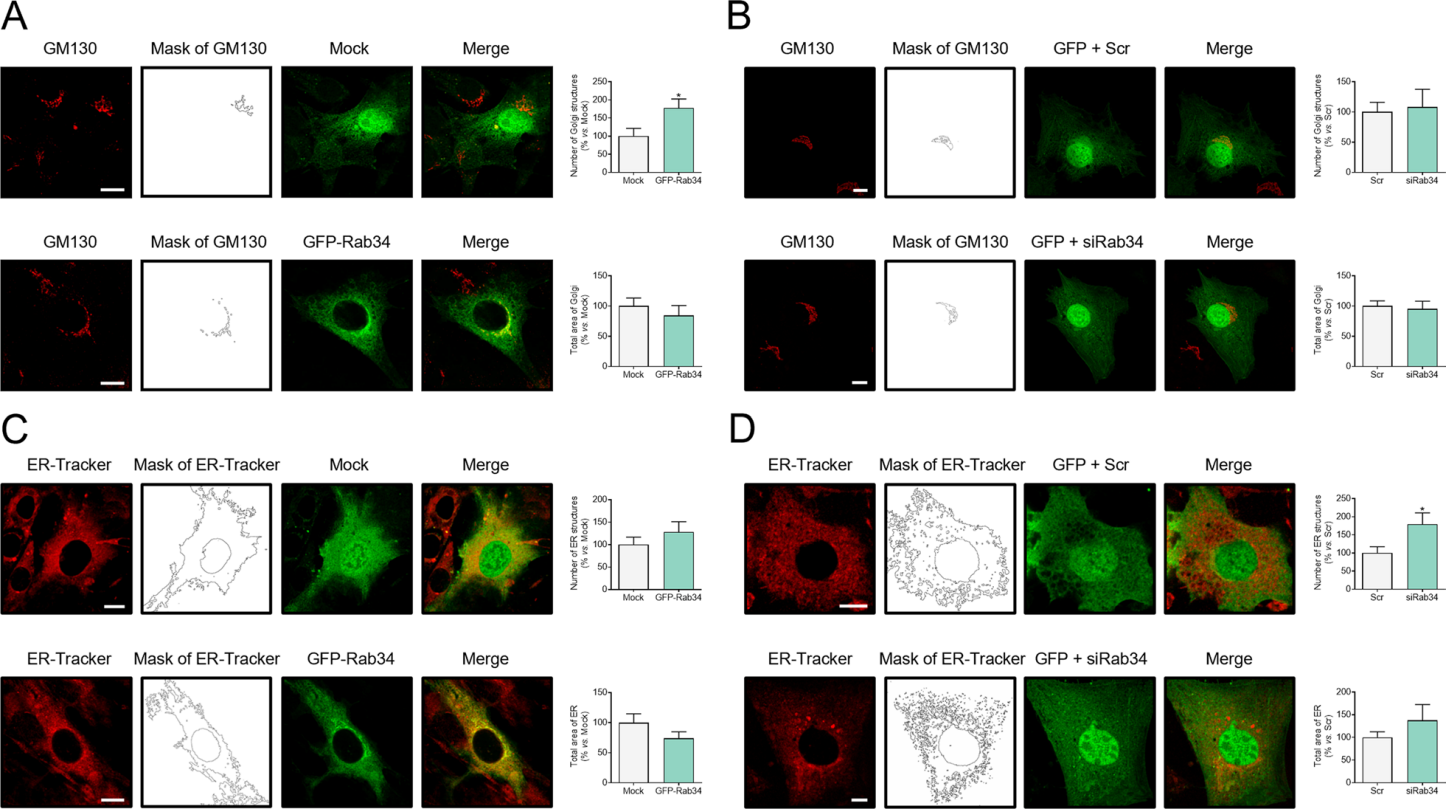
Effects of Rab34 expression/silencing on Golgi/ER structure in 3T3-L1 adipocytes. **A**–**D** Representative images of 3T3-L1 cells transfected with GFP (Mock) or GFP-Rab34 vectors and Scramble siRNA (Scr) or Rab34 siRNA (siRab34), and stained with anti-GM130 (**A**,** B**) or ER-Tracker (**C**,** D**). Cells were transfected at day 3 and collected at day 5 (expression studies) or day 6 (silencing studies) of differentiation. Morphometric analyses were carried out using ImageJ software. The number of GM130-positive structures and total Golgi area (**A**,** B**), and ER-positive structures and total ER area (**C**,** D**) in cells expressing GFP-Rab34 or siRab34 are referred to their corresponding controls (Mock/Scr; 100%). Data are expressed as mean ± SEM (n = 10 cells/experimental condition, 2 replicate studies). Scale bar: 10 μm

Based on the localization of Rab34 in preadipocytes, we next investigated whether this protein may be involved in Golgi apparatus organization and membrane trafficking. To this end, the effects of GFP-Rab34 expression or transient reduction by siRNA-dependent silencing on the structure of both the Golgi apparatus and the ER were analyzed (48 or 72 h after transfection for expression and silencing studies, respectively). For expression experiments, a vector coding only for GFP (mock) was used. Consistently with our previous observations [27, 44], mock transfected 3T3-L1 cells showed a diffuse and non-specific green labeling throughout the cytoplasm and the nucleus (Figs. 3A, C). Colocalization of the GFP empty vector with the Golgi matrix protein, GM130, and an endoplasmic reticulum (ER) marker showed a significantly low overlapping in both cellular compartments when compared to GFP-Rab34 labeling (data not shown). GFP-Rab34 expression caused a significant increase in the number of fragments of the Golgi apparatus without affecting its total area (Fig. 3A). In contrast, Rab34 silencing had no effect on these two parameters (Fig. 3B). Notably, Rab34 depletion induced ER fragmentation, while its expression changed neither the number of ER structures nor the ER total area (Figs. 3C, D). These results suggested that Rab34 gain-of-function is more important to the Golgi organization than its loss, and the opposite holds true for the ER. Together, these results indicated that Rab34 may act as a Class 2 Golgi-associated Rab protein and relate to Golgi-to-ER transport pathways, as suggested for other Rab proteins with similar effects on the Golgi upon activation *vs.* inactivation [45].

### Role of Rab34 on adipocyte function

The presence of Rab34 from early stages of adipocyte differentiation (at the Golgi) to fully differentiated mature adipocytes (at the LDs) suggested the participation of this GTPase in the regulation of adipocyte functions, namely: i) as an endocrine cell, and/or ii) as a central hub for energy storage and release [7].

#### Rab34 regulates adiponectin secretion and oligomerization

**Fig. 4:**
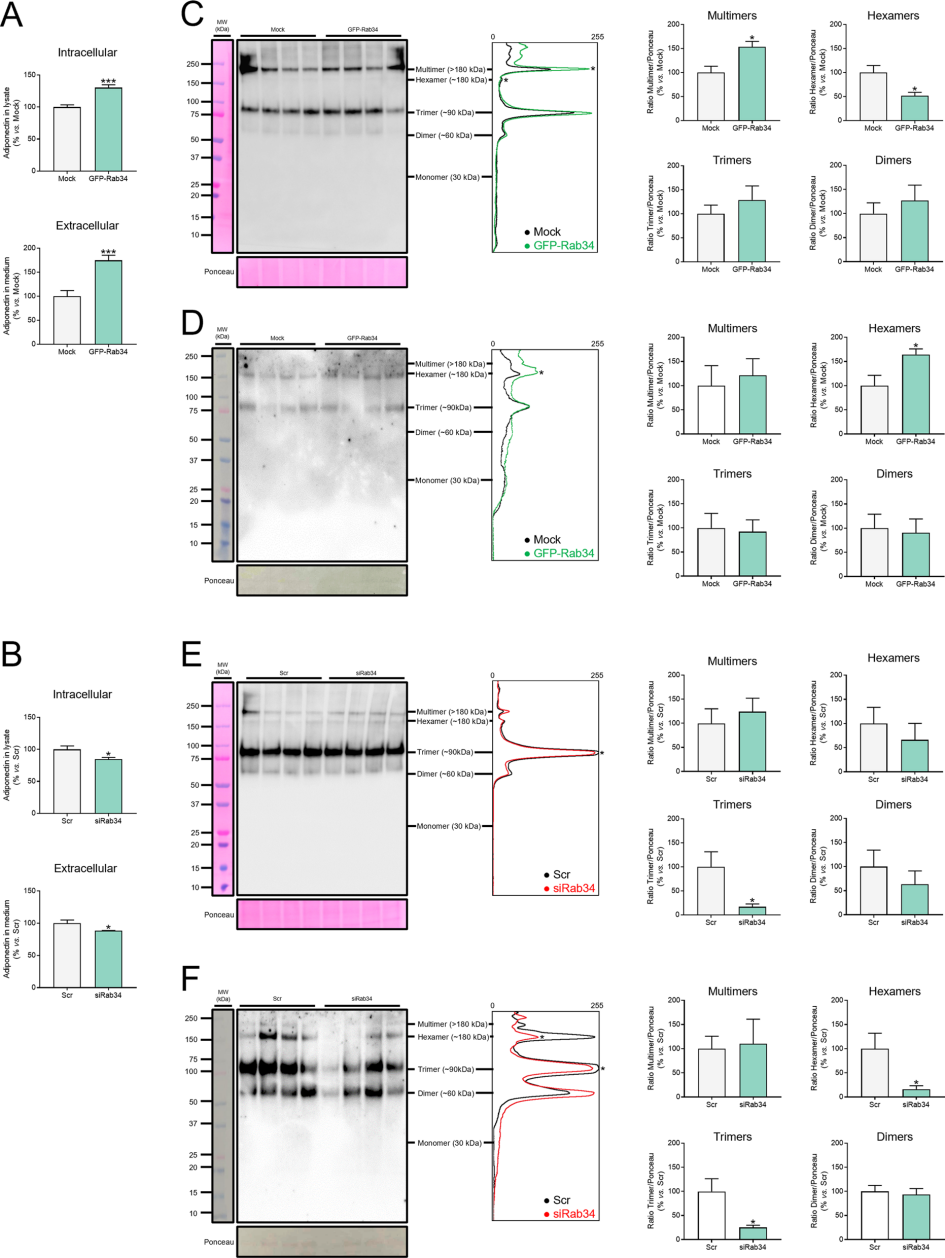
Effects of Rab34 expression/silencing on the secretory pathway in 3T3-L1 adipocytes. **A**–**F** Quantification of adiponectin secretion and oligomerization in control cells (Mock/Scr) and cells expressing GFP-Rab34 (**A**, **C**, **D**) or siRab34 (**B**, **E**, **F**). **A**, **B** Adiponectin content in protein extracts (intracellular) and the culture media (extracellular) in control cells and cells expressing GFP-Rab34 (**A**) or transfected with siRab34 (**B**) were analyzed by ELISA (n = 6 biological replicates). **C**–**F** Representative immunoblots and quantification of adiponectin multimers, hexamers, trimers and dimers in intra- and extracellular extracts from control cells and cells expressing GFP-Rab34 (**C**, **D**) or transfected with siRab34 (**E**, **F**). Cells were transfected at day 3 and collected at day 5 (expression studies) or day 6 (silencing studies) of differentiation. Cell samples were collected and the proteins were resolved in non-reducing SDS-PAGE gels with a 4–20% gradient and subjected to Western blot and densitometric analysis. Ponceau S was used as loading control. Data are referred to values in control cells (Mock; Scr) (100%) and expressed as the mean ± SEM (n = 4 biological replicates). *P < 0.05; ***P < 0.001 *vs.* Mock/Scr

To determine the impact of Rab34 on adipocyte function, we first analyzed whether changes in its levels could affect the secretory activity of adipocytes by assessing the production of adiponectin, a major adipokine with insulin-sensitizing and anti-inflammatory properties [46]. Quantification of intra- and extracellular adiponectin levels in 3T3-L1 adipocytes by ELISA showed that while GFP-Rab34 expression increased the synthesis and secretion of adiponectin (Fig. 4A), siRab34 treatment decreased both parameters (Fig. 4B). Furthermore, analysis of intracellular and extracellular protein content using non-denaturing conditions revealed that GFP-Rab34 expression increased the accumulation of adiponectin multimers (Figs. 4C, D). However, intracellular adiponectin hexamers decreased, an effect that occurred in parallel to an increase in the amount of these adiponectin isoforms in the culture media (Figs. 4C, D). Rab34 depletion decreased intracellular adiponectin trimer content and both trimer and hexamer release to the culture medium (Figs. 4E, F).

#### Rab34 is a regulatory component of the lipid droplet coat in adipocytes

**Fig. 5:**
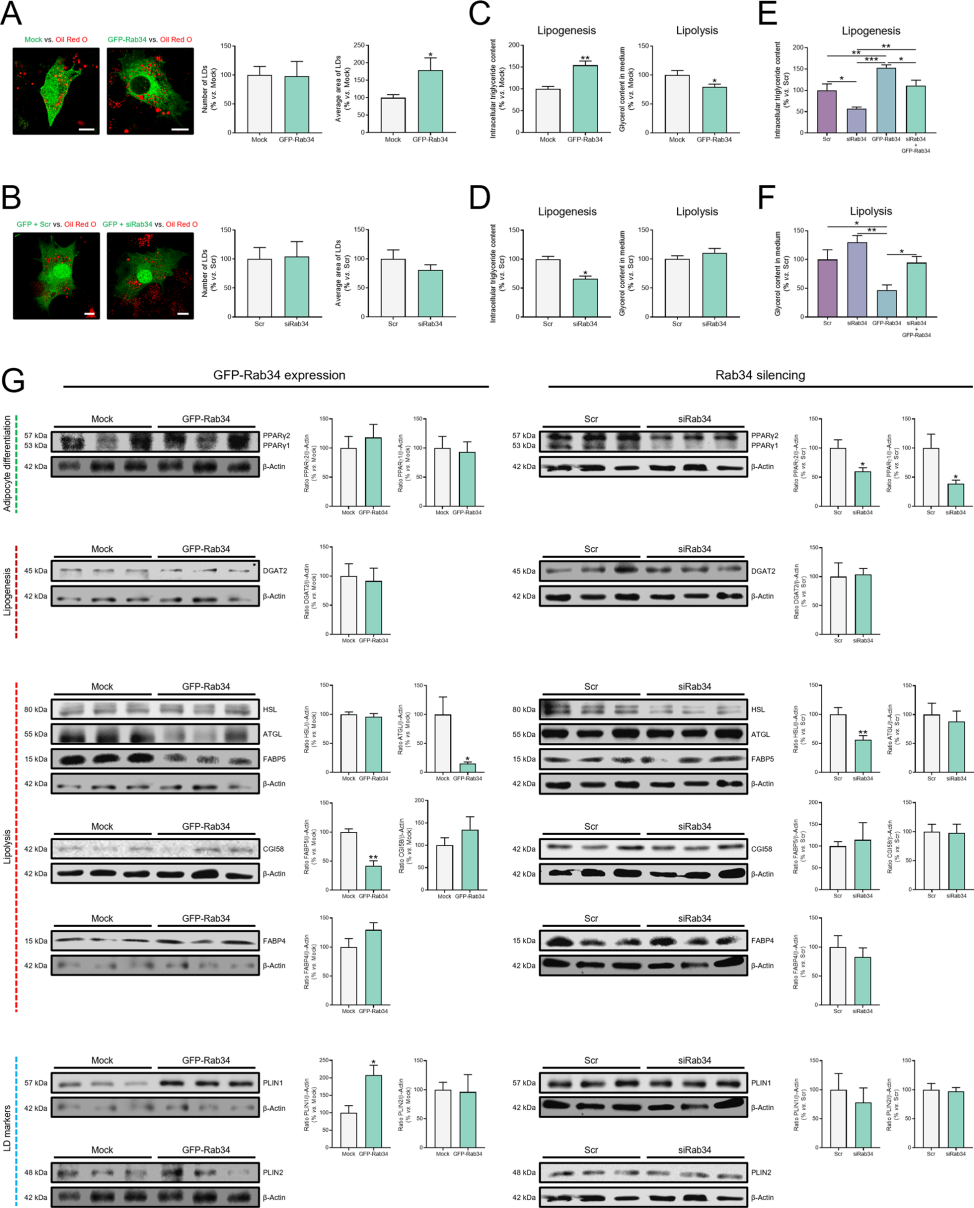
Effects of Rab34 expression/silencing on lipid accumulation in adipocytes. **A**, **B** Representative images of 3T3-L1 cells transfected at day 3 of differentiation with GFP (Mock), GFP-Rab34, Scramble siRNA (Scr) or Rab34 siRNA (siRab34) and stained with Oil Red O at day 5 (expression, **A**) or day 6 (silencing, **B**) of differentiation. Morphometric analyses were carried out using ImageJ software. The number and average area of LDs was calculated per cell and referred to values in control cells (100%) (Mock, **A**; Scr, **B**). Data are expressed as mean ± SEM (n = 10 cells/experimental condition, 2 replicate studies). Scale bar: 10 μm. **C**, **D** Quantification of TG content (lipogenesis) and glycerol release (lipolysis) in each condition. Data are referred to values in control cells (Mock; Scr) (100%) and expressed as mean ± SEM (n = 4 biological replicates). **E**, **F** Measurement of triglyceride content (lipogenesis) and glycerol release (lipolysis) in cells expressing GFP-Rab34 or siRab34, alone or in combination (Rab34 recovery). Data are referred to control cultures (Scr) (100%) and expressed as mean ± SEM (n = 3 biological replicates). **G** Quantification of the protein levels of markers of adipocyte differentiation, lipogenesis, lipolysis, and LD markers in 3T3-L1 cells transfected with Mock (GFP) or Rab34 (GFP-Rab34) expression vectors (left panel), or Scramble (Scr) or Rab34 siRNA (siRab34) (right panel). Cells were transfected at day 3 and collected at day 5 (expression studies) or day 6 (silencing studies) of differentiation. The β-Actin immunoreactive band in each membrane was employed as a reference for quantification of the corresponding proteins that were revealed in the same blot. Data correspond to the ratio of each immunosignal to β-actin immunosignal and are referred to values in control cells (Mock; Scr) (100%). Data represent the mean ± SEM (n = 3 biological replicates). *P < 0.05; **P < 0.01; ***P < 0.001

Based on our results showing the association of Rab34 with LDs from D3 onwards (Fig. 1B), we decided to explore whether Rab34 up- or down-regulation (Additional file 3: Fig. S2A) may affect LD number and size at later stages of differentiation. These studies showed that LD size increased by 78.9% while no changes were observed in LD number as compared with mock-transfected cells upon GFP-Rab34 expression (Fig. 5A). In line with these observations, quantification of intracellular TGs and free glycerol in the cell culture medium in cells expressing GFP-Rab34 indicated that intracellular lipid content was increased by 54.1% and basal lipolysis was decreased by 20.5% (Fig. 5C). On the other hand, Rab34 down-regulation by siRNA had no significant effects on LD number though the average size tended to decrease (Fig. 5B). A deeper look, analyzing the size-frequency distribution of LDs, showed that siRab34 treatment increased the number of medium LDs (6–9 mm^2^) while decreased that of small LDs (< 3 mm^2^) (Additional file 3: Fig. S2B), which could account for the down-regulation of TG content (Fig. 5D). Notably, rescue experiments returning Rab34 protein levels to basal values (Additional file 3: Fig. S2C) after expression of GFP-Rab34 in cells transfected with siRab34, clearly reverted the effects induced by this GTPase on TG content (Fig. 5E) and lipolysis (Fig. 5F).

Next, to get insights into the mechanisms mediating Rab34 action on lipid accumulation in LDs, we examined the effect of Rab34 expression or silencing (Additional file 3: Fig. S2A) on the regulation of markers of adipocyte differentiation (PPARγ1 and PPARγ2), as well as of proteins involved in TG accumulation into LDs (ACSL1, GPAT, DGAT2), de novo lipogenesis (FASN), lipolysis (p-HSL, HSL, ATGL, the ATGL coactivator CGI58, and the lipid chaperones, FABP4 and FABP5), and LD growth and/or turnover (PLIN1 and PLIN2) [47] (Fig. 5G, Additional file 3: Figs. S2D, E). Quantitative immunoblotting analyses revealed that Rab34 up-regulation increased PLIN1 content and decreased both ATGL and FABP5 (Fig. 5G, left panel). On the other hand, Rab34 down-regulation diminished the expression of PPARγ1 and PPARγ2 as well as the protein content of total HSL (Fig. 5G, right panel). Changes in phosphorylated (i.e., active) HSL levels paralleled those of total HSL and consequently, no differences were observed between control and experimental groups in p-HSL/HSL ratio (data not shown). Neither Rab34 overexpression nor silencing evoked changes in mRNA or protein levels of enzymes involved in TG storage (Fig. 5G, Additional file 3: Fig. S2D) or de novo lipogenesis (Additional file 3: Fig. S2E). As observed for the wild-type Rab34 version, the constitutively active variant (GFP-Rab34-Q111L) increased TG content and decreased lipolysis (Additional file 3: Fig. S2F). Expression of GFP-Rab34-Q111L also reduced ATGL and FABP5 protein content and increased that of PLIN1 (Additional file 3: Fig. S2G). On the other hand, GFP-Rab34-T66N modified neither the protein content of these proteins (Additional file 3: Fig. S2G) nor lipogenesis, though lipolysis was increased as compared to mock-transfected cells (Additional file 3: Fig. S2F). Altogether, these results strongly support the involvement of Rab34 in lipid accumulation in adipocytes, likely by regulating lipolysis.

### Rab34 interactome

Rab GTPases functions depend on their interaction with specific sets of downstream effector proteins that are recruited to target membranes [48]. Rab effectors include molecular motors, tethers, kinases, phosphatases, components of membrane contact sites and sorting adaptors, as well as Rab regulators [GTPase-activating proteins (GAPs) and Guanine nucleotide exchange factors (GEFs)] [49].

**Fig. 6:**
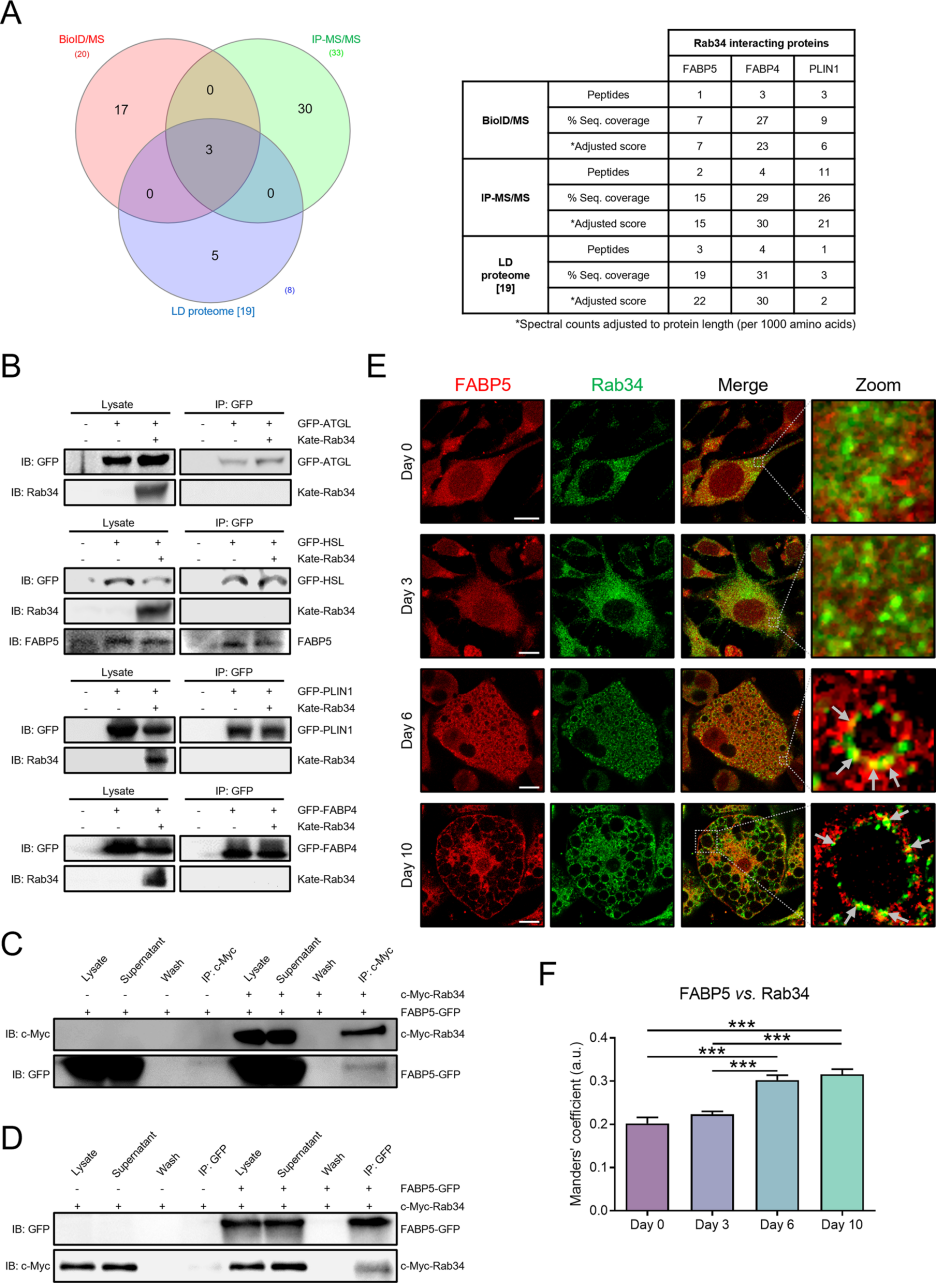
Identification of the Rab34 Interactome. **A** Venn diagram of the proteins participating in the “Metabolism of lipids” pathway from Reactome Pathways dataset that were identified by the three proteomic approaches analyzed in this study: (i) BioID/MS (20 proteins); (ii) IP-MS/MS (33 proteins); and (iii) Proteome of adipocyte LDs (8 proteins) [19]. Among them, the lipid chaperones, FABP4 and FABP5, and the LD-associated protein, PLIN1, were present in the three proteomic datasets. Spectral counts were adjusted to protein length (per 1000 amino acids). **B** Co-immunoprecipitation experiments in HEK-293 AD cells expressing Kate-Rab34 and either GFP-ATGL, GFP-HSL, GFP-PLIN1, and GFP-FABP4 using anti-GFP beads. Both lysates and immunoprecipitates (IP) were subjected to immunoblotting with anti-GFP and anti-Rab34; anti-FABP5 antibodies were also used for studies using GFP-HSL. **C**, **D** Co-immunoprecipitation experiments in HEK-293 AD cells expressing c-Myc-Rab34 and FABP5-GFP, alone or in combination, using anti-c-Myc-beads (**C**) or anti-GFP-beads (**D**). Immunoblotting studies of lysates and IP were carried out using anti-c-Myc (**C**) or anti-GFP antibodies (**D**). **E** Representative confocal images of 3T3-L1 cells showing the colocalization (merge) of Rab34 (green) and FABP5 (red) during differentiation (days 0, 3, 6 and 10). Arrows indicate Rab34/FABP5 colocalization (yellow) at the LD surface. **F** Manders’ coefficient between Rab34 and FABP5 was calculated to quantify the degree of colocalization between both signals. Data represent the mean ± SEM (n = 10 cells/differentiation day, 2 replicate studies). ***P < 0.001. Scale bar: 10 μm

In order to identify potential effectors of Rab34, we employed BioID [36] and immunoprecipitation studies using c-Myc-BirA-Rab34 and GFP-Rab34 as expression vectors, respectively, both followed by MS/MS analysis (Additional file 4). Based on our expression and silencing data suggesting the involvement of Rab34 in the regulation of lipogenesis/lipolysis, we filtered the protein lists obtained from BioID/MS and IP-MS/MS analyses for proteins related to “Metabolism of lipids” according to Reactome Pathways (Additional file 5). After further filtering for matching proteins in the two lists, we found FABP5, FABP4, and PLIN1 as potential Rab34 interactors. Moreover, the three proteins have been also identified by our group as components of the LD proteome from 3T3-L1 adipocytes [19] (Fig. 6A; Additional file 5). However, none of these proteins but FABP5 were found to interact with Rab34 in targeted immunoprecipitation studies in HEK293 cells (Figs. 6B–D). Likewise, none of the lipolytic enzymes that we found dysregulated in 3T3-L1 adipocytes in response to Rab34 silencing and/or expression (i.e., HSL and ATGL) were found to co-immunoprecipitate with the GTPase (Fig. 6B).

The interaction between Rab34 and FABP5 was confirmed using either c-Myc-Rab34 (Fig. 6C) or GFP-FAPB5 (Fig. 6D) for the pulldown experiments. Interestingly, when cells were transfected with pEGFP-Rab34-Q111L or pEGFP-Rab34-T66N and pFABP5-EGFP, only the former co-immunoprecipitated with FABP5, indicating that the GTPase needs to be bound to GTP (active) to interact with FABP5 (Additional file 3: Fig. S2H). In addition, confocal microscopy studies showed that FABP5 colocalized with Rab34 at the LDs as soon as these organelles accumulate in the cytosol (from D3 onwards) (Figs. 6E, F), suggesting that this interaction would occur at the LD surface. Altogether, these studies identify FABP5 as a potential Rab34 interactor in adipocytes. Our studies also confirmed and extended previous publications on the interaction between FABP5 and HSL (Fig. 6B) [50].

### Rab34 interacts with FABP5 to regulate lipid turnover in LDs

**Fig. 7:**
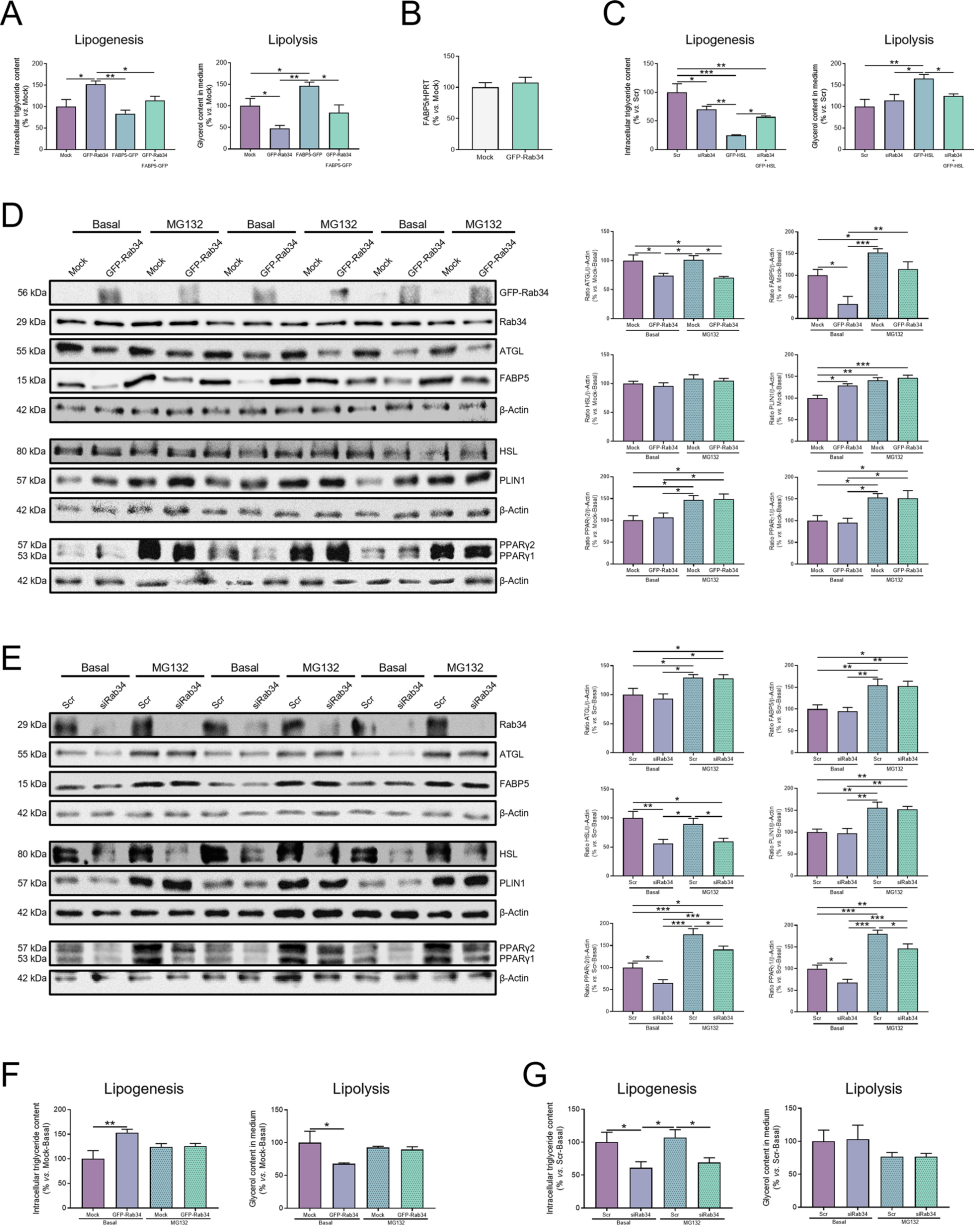
Rab34 actions on lipid metabolism are mediated by FABP5 in a proteasome-dependent manner. **A** Quantification of the lipogenic and lipolytic activities of 3T3-L1 cells expressing GFP-Rab34, FABP5-GFP or a combination of both expression vectors as compared to cells expressing the empty GFP vector (Mock). **B** RT-qPCR analysis of FABP5 mRNA expression levels in 3T3-L1 cells expressing GFP-Rab34. FABP5 mRNA levels were calculated using the Ct method and HPRT as housekeeping gene, and expressed as the mean ± SEM (n = 3 biological experiments). **C** Quantification of the lipogenic and lipolytic activities of 3T3-L1 cells expressing siRab34, GFP-HSL or a combination of both nucleic acids as compared to cells transfected with scramble siRNA (Scr). (**D**,** E**) Quantitative immunoblotting analysis of Rab34, ATGL, FABP5, HSL, PLIN1, PPARγ1, and PPARγ2 in 3T3-L1 cells expressing GFP-Rab34 (**D**) or siRab34 (**E**), and exposed or not (Basal) to the proteasome inhibitor, MG132 (10 μmol/L, 12 h). Cells transfected with the empty GFP vector (Mock) or scramble siRNA (Scr) were employed as controls. Graphs show the ratio of each immunosignal to β-actin immunosignal. Data are expressed as the mean ± SEM (n = 3 biological replicates). **F**,** G** Lipogenic and lipolytic activities of 3T3-L1 cells expressing GFP-Rab34 (**F**) or siRab34 (**G**) and exposed or not (Basal) to the proteasome inhibitor, MG132 (10 μmol/L, 12 h). Data are expressed as a percentage of values in control cultures and represent the mean ± SEM (n = 3 individual experiments). All data are referred to values in control cells (100%) (Basal; Mock/Scr). *P < 0.05; **P < 0.01; ***P < 0.001

To get insights into the functional interaction between Rab34 and FABP5 in the regulation of lipid accumulation, we examined the effects of the co-expression of these two proteins on the lipogenic and lipolytic activities of 3T3-L1 adipocytes (Fig. 7A; Additional file 3: Fig. S3A). As shown in Fig. 7A, expression of GFP-FABP5 alone increased the amount of glycerol released by adipocytes, without changing basal TG content, thus confirming the role of this chaperone in stimulating lipolysis [51]. Our co-immunoprecipitation studies (Fig. 6B) and previous reports [50] support the notion that FABP5 effects could be mediated through its interaction with HSL, as shown for FABP4 [52].

Notably, the inhibitory effect of GFP-Rab34 expression on lipolysis was reverted upon co-transfection with GFP-FABP5 (Fig. 7A) and, conversely, TG accumulation induced by Rab34 up-regulation decreased to control levels upon FAPB5-GFP expression (Fig. 7A). These results suggested that Rab34 negative action on lipolysis and concomitant induction of lipid accumulation would be mediated, at least in part, through Rab34-mediated reduction of FABP5 protein content. Interestingly, Rab34 expression had no effect on FABP5 mRNA levels (Fig. 7B).

We also analyzed the functional interaction between Rab34 and HSL. As previously shown for FABP5-GFP, expression of GFP-HSL in adipocytes increased lipolysis yet the lipase also caused a concomitant decrease in TG content (Fig. 7C; Additional file 3: Fig. S3B). Rab34 gene silencing blocked HSL-induced effects on lipolysis but not on lipogenesis (Fig. 7C). These observations, together with our immunoprecipitation studies, suggested a functional interplay between Rab34 and HSL that does rely on an indirect interaction between the two proteins.

### Rab34 regulates FABP5 protein stability

The observation that Rab34 up-regulation reduced FABP5 protein content (Fig. 5G; Additional file 3: Fig. S3A), but not FABP5 gene expression (Fig. 7B), prompted us to investigate whether the GTPase may modulate the protein stability of this chaperone. To this end, we first measured the protein levels of FABP5 and other proteins regulated by Rab34 in cells treated with the proteasome inhibitor MG132 (Figs. 7D, E; Additional file 3: Figs. S3C, D). As shown in Figs. 7D and E, MG132 treatment increased the protein content of FABP5, PLIN1, PPARγ1, PPARγ2, and ATGL in control cells (either transfected with GFP alone or scramble siRNA). Among the proteins tested, only FABP5 protein levels increased to control values in the presence of MG132 in cells expressing GFP-Rab34 (Fig. 7D), suggesting the involvement of the GTPase in the regulation of FABP5 turnover. Interestingly, MG132 also reverted both Rab34-mediated increase in lipogenesis and decrease in lipolysis, while no changes in these parameters were observed when this inhibitor was administered to mock-transfected cells (Fig. 7F). However, MG132 had no effect on the inhibition of lipogenesis caused by Rab34 siRNA treatment (Fig. 7G). Lastly, silencing experiments also revealed that MG132 partially reverted PPARγ down-regulation in adipocytes exposed to Rab34 siRNA (Fig. 7E).

### E1 Ubiquitin-activating enzyme (UBA1) participates in Rab34-mediated regulation of FABP5 actions on lipid storage and mobilization in adipocytes

**Fig. 8:**
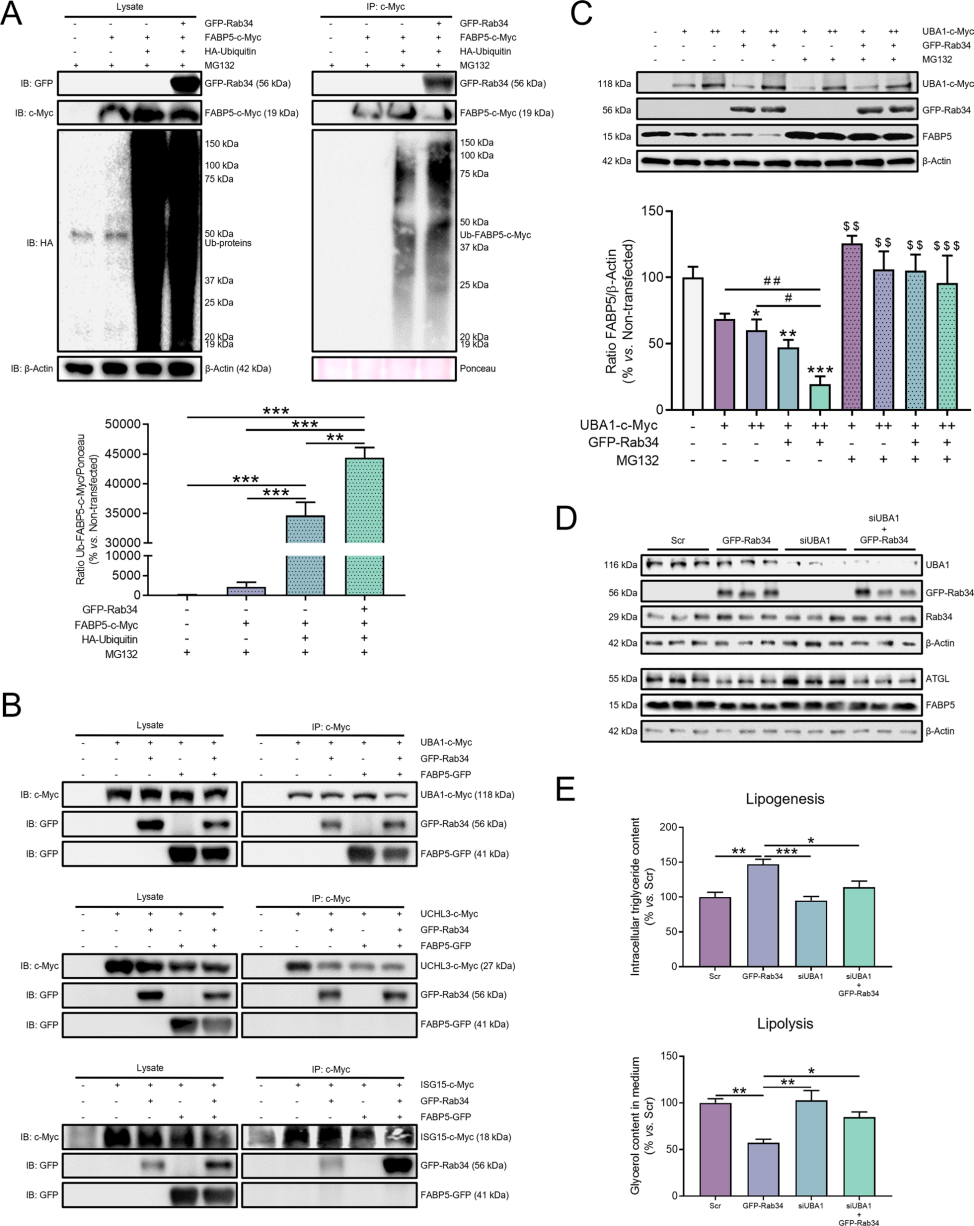
UBA1 conveys Rab34 action on FABP5 stability to regulate lipid metabolism. **A** Transfected 3T3-L1 cells with the indicated plasmids (GFP-Rab34 or FABP5-c-Myc) were treated with MG132 (10 μmol/L, 12 h) and lysed under denaturing conditions. c-Myc-tagged FABP5 was purified by anti-c-Myc immunoprecipitation and ubiquitinated FABP5 was detected by Western Blot. An expression vector coding for hemagglutinin (HA)-tagged ubiquitin (HA-Ubiquitin) was employed for cotransfection of cells expressing FABP5-c-Myc, alone or in combination with GFP-Rab34. The graph shows the ratio of Ubiquitinated-FABP5-c-Myc immunosignal to Ponceau S immunosignal. Data are referred to values in non-transfected cells (100%) and expressed as mean ± SEM (n = 3 biological replicates). **P < 0.01; ***P < 0.001. **B** Co-immunoprecipitation analysis in cells expressing GFP-Rab34 and vectors coding for LD-associated proteins related to ubiquitination/deubiquitination processes: UBA1-c-Myc (top panel), UCHL3-c-Myc (middle panel), or ISG15-c-Myc (bottom panel). In each experimental setting, proteins were purified by anti-c-Myc immunoprecipitation and detected by Western Blot using anti-c-Myc or anti-GFP antibodies. **C** Representative immunoblots and quantification of FABP5 levels in 3T3-L1 cells transfected with GFP-Rab34 (+ , 0.8 µg/µL), UBA1-c-Myc (+ , 0.8 µg/µL; +  + , 1.6 µg/µL) or both expression vectors in the absence or presence of MG132 (10 µmol/L, 12 h). Data represent the ratio of FABP5 immunosignal to β-actin immunosignal and referred to values in non-transfected cells (100%). Data are expressed as mean ± SEM (n = 3 biological replicates). *P < 0.05; **P < 0.01; ***P < 0.001 *vs.* non-transfected cells. ^$$^P < 0.01; ^$$$^P < 0.001 *vs.* their respective condition treated with MG132. ^#^P < 0.05; ^##^P < 0.01. **D, E** Rescue experiments of FABP5 in 3T3-L1 cells expressing GFP-Rab34 and UBA1 siRNA (siUBA1), alone or in combination. At the end of the experiments, cells were processed for immunoblotting studies (**D**) (see also Fig. S4) and for measurement of TGs (lipogenesis) and glycerol content (lipolysis) (**E**). Data are referred to values in control cells (100%; Scr), and expressed as mean ± SEM (n = 3 biological replicates). *P < 0.05; **P < 0.01; ***P < 0.001

Our previous results suggested that Rab34 actions on lipolysis and TG storage might be mediated through the regulation of FABP5 ubiquitination and proteasomal degradation. To address this question, we expressed FABP5, alone or in combination with Rab34 and/or an expression vector coding for hemagglutinin (HA)-tagged ubiquitin (Fig. 8A). These experiments were carried out in cells exposed to MG132 to prevent FABP5 degradation. Quantification of the immunoblots stained for an anti-HA serum revealed that the levels of ubiquitinated FABP5 were significantly higher in cells expressing Rab34 (Fig. 8A).

These observations led us to test whether there could be enzymes involved in ubiquitination or deubiquitination among the potential effectors of Rab34 action on FABP5. To be more specific, we checked the protein lists obtained from our BioID and IP-MS/MS studies for the presence of ubiquitinating (UBs) and/or deubiquitinating (DUBs) enzymes (Additional file 6). Among the proteins identified, we found: i) UBA1 (BioID and IP-MS/MS data lists), which catalyzes the first step in ubiquitin conjugation to mark cellular proteins for degradation [53], ii) UCHL3 (BioID protein list), an ubiquitin C-terminal hydrolase [54], and iii) ISG15 (IP-MS/MS protein list), an ubiquitin-like protein [53]. Remarkably, these three proteins were also detected in our previous proteomic study of isolated LDs from 3T3-L1 adipocytes [19] (Additional file 6). Thus, we next checked whether these proteins may interact with Rab34 and/or FABP5 in co-immunoprecipitation studies. These experiments confirmed that the three proteins can interact with Rab34, though only UBA1 interacted with FABP5, alone or in combination with Rab34 (Fig. 8B).

Next, to test the possible participation of UBA1 in FABP5 ubiquitination and degradation in response to Rab34, we evaluated FABP5 levels in protein extracts from 3T3-L1 adipocytes transfected with UBA1-c-Myc and/or GFP-Rab34 in the presence or absence of MG132. Interestingly, UBA1 expression decreased FABP5 content in a concentration-dependent manner, especially in the presence of Rab34, and this effect was fully blocked by MG132 treatment (Fig. 8C). Likewise, UBA1 depletion by siRNA (82.9% with respect to control values) did not modify the protein content of Rab34 or ATGL (Fig. 8D; Additional file 3: Figs. S4A–C). On the contrary, UBA1 silencing fully prevented Rab34-mediated decrease in FABP5 content (Fig. 8D; Additional file 3: Fig. S4D).

Finally, functional assays in 3T3-L1 adipocytes transfected with siUBA1 and/or GFP-Rab34 demonstrated that UBA1 down-regulation prevented the effects of Rab34 on both lipid accumulation and hydrolysis (i.e., decreased lipogenesis and increased lipolysis, respectively) (Fig. 8E). Taken together, these findings provide strong evidence for a functional connection between Rab34 and UBA1 in the regulation of FABP5 ubiquitination and proteasomal degradation. In this line, double immunolabeling studies using antisera against Rab34 and UBA1 revealed that the two proteins colocalized at the LD surface, especially in adipocytes at D6 and D10 of differentiation (Additional file 3: Fig. S4E).

### Rab34 in human adipocytes and disease models

**Fig. 9:**
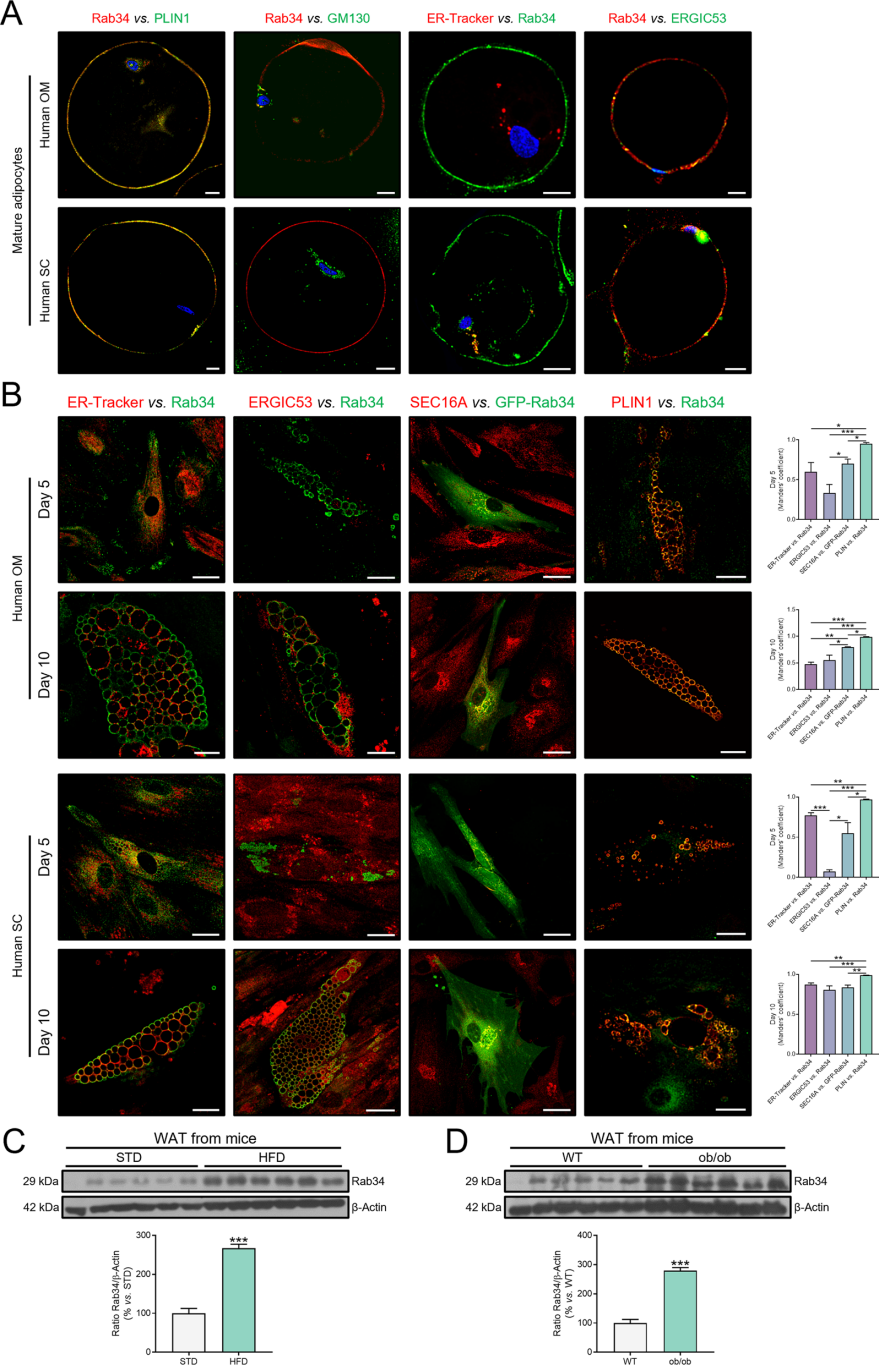
Characterization of Rab34 in human adipocytes and animal models of obesity. **A** Representative confocal microscopy images of mature adipocytes isolated from human omental (OM) and subcutaneous (SC) adipose tissue double-immunostained with the anti-Rab34 antibody and antibodies against PLIN1, GM130, and ERGIC53 or labeled with the ER-Tracker. **B** Representative confocal microscopy images of human OM and SC preadipocytes at different days of differentiation (day 5 and 10). Cells were incubated with the anti-Rab34 antibody and either antibodies against ERGIC53 or PLIN1 or the ER-Tracker. For detection of ERES, cells expressing GFP-Rab34 were incubated with an anti-SEC16A antibody. Manders’ coefficients were calculated to assess the colocalization between signals. Data are expressed as the mean ± SEM (n = 13 cells/differentiation day, 2 replicate studies). **C**, **D** Representative immunoblots and quantification of Rab34 levels in white adipose tissue (WAT) samples from mice fed standard (STD) *vs*. high-fat diet (HFD) (**C**), and wild-type (WT) *vs*. ob/ob mice (**D**). Data are referred to values in control mice (100%), and expressed as mean ± SEM (n = 6). *P < 0.05; **P < 0.01; ***P < 0.001. Scale bar: 10 μm

Analysis of published data from transcriptomic studies of human abdominal SC fat samples [55, 56] showed that Rab34 transcript content is higher in individuals with obesity than in lean individuals (Additional file 3: Fig. S5A). From this perspective, we then decided to explore whether the results in the 3T3-L1 cell line could be also extended to human primary adipocytes (mature adipocytes and preadipocytes, either freshly isolated or differentiated in vitro). To this end, we analyzed Rab34 localization in mature adipocytes isolated from OM and SC samples obtained from individuals with obesity during bariatric surgery procedures (Additional file 1). As shown in Fig. 9A, most of Rab34 immunosignal colocalized with the unilocular LD in mature adipocytes, although a slight residual signal could be also seen at the Golgi (more visible in OM mature adipocytes). We also explored the distribution of Rab34 in relation to both the ER and the ERGIC domain. We observed that Rab34 partially colocalized with the ER-Tracker in SC mature adipocytes and with ERGIC53 in both OM and SC mature adipocytes (Fig. 9A).

Regarding in vitro differentiation studies, preadipocytes isolated from the SVF of OM and SC fat samples were cultured and differentiated to mature adipocytes using standard protocols (Fig. 9B) [27]. In particular, we analyzed the localization of Rab34 signal in relation to the LDs and the ER, including the ER subdomains, ERGIC and ERES, at different stages of differentiation (D5 and D10) (Fig. 9B). Irrespective of the fat depot, Rab34 immunosignal overlapped with the LD surface marker (PLIN1). Notably, Rab34 immunosignal clearly colocalized with UBA1 and FABP5 signals at the LD surface in both SC and OM adipocytes (Additional file 3: Fig. S4F).

In addition, an extensive colocalization of Rab34 immunosignal with the ER-Tracker was observed in both OM and SC adipocytes throughout differentiation. As previously observed in 3T3-L1 cells, Rab34 is associated with ER subdomains in differentiating human adipocytes. Quantitative analysis of colocalization signals in double labeling experiments showed a higher overlap between Rab34 and the ERES marker, SEC16A, than with ERGIC53 at either D5 or D10 in OM adipocytes (Fig. 9B). In fact, at D10, the colocalization rate of Rab34-SEC16A was higher than with the ER tracker. In SC adipocytes at D5, colocalization between Rab34 and ERGIC53 was almost negligible, and most of the Rab34 immunosignal overlapped with SEC16A (Fig. 9B). At late stages of differentiation, SC adipocytes displayed similar rates of colocalization between Rab34 and SEC16A or ERGIC53 (Fig. 9B). In GFP-Rab34-transfected cells, the signal was also observable in the Golgi apparatus (Fig. 9B), as we had also observed for 3T3-L1 cells (Fig. 1C).

We also evaluated Rab34 expression in different murine models of obesity. In this context, we first analyzed Rab34 protein content in the adipose tissue of diet- or genetically-induced obese mice (Figs. 9C, D). The quantification showed that Rab34 levels increased by 2.7-fold in protein extracts from WAT fed a HFD compared to mice on a STD (Fig. 9C). Similarly, Rab34 levels increased by 2.8-fold in protein extracts of WAT from ob/ob mice compared to WT mice (Fig. 9D). These results, together with those previously depicted showing the role of Rab34 on adipocyte function, indicate that Rab34 expression in the adipose tissue is directly related to the development of obesity, behaving as a biomarker of this disease.

Finally, based on the results obtained in human and animal models, we wanted to test whether, in addition to changes in Rab34 content, the localization of this GTPase may be also affected under obesity conditions. To this end, we employed a 3D culture model of adipocytes previously validated by us that mimics the fibrotic microenvironment found in the adipose tissue of individuals with obesity and insulin resistance [19]. As shown in Additional file 3: Fig. S5B, culture of 3T3-L1 adipocytes in COL-I-based 3D microgels enriched in the proteoglycan, lumican (i.e., obesity conditions), induced a decrease in the number of LDs immunostained for Rab34, though no changes were observed in total Rab34 immunosignal per cell. Together, these observations suggest that obesity-related insults may modify Rab34 association with LDs.

## Discussion

The initial view of LDs as simple organelles responsible for energy storage has changed notably in recent years. In this way, it is currently accepted that they are highly dynamic and complex organelles involved in important cellular processes, which necessarily include the storage and mobilization of TGs in a strictly regulated manner [57, 58]. This paradigm shift has been largely supported by the identification of the proteins associated with the LD coat of adipocytes and other cell types [19, 59–61]. Pioneering proteomic studies of LDs by Brasaemle et al. revealed that stimulation of lipolysis in 3T3-L1 adipocytes by exposure to β-adrenergic agonists induced the recruitment of proteins such as PLIN2, CAV1, and Rab GTPases (i.e., Rab18) to the LD surface [62]. More recently, a proteomic study of LDs isolated from differentiated 3T3-L1 adipocytes allowed us to identify the presence of an additional Rab family member, Rab34, associated with the LD cover, both under unstimulated conditions and upon insulin stimulation [19]. Herein, we provide experimental evidence supporting a regulatory role for Rab34 in lipid storage in LDs by modulating the availability at the LD surface of the lipid chaperone and regulator of lipolysis, FABP5.

**Fig. 10:**
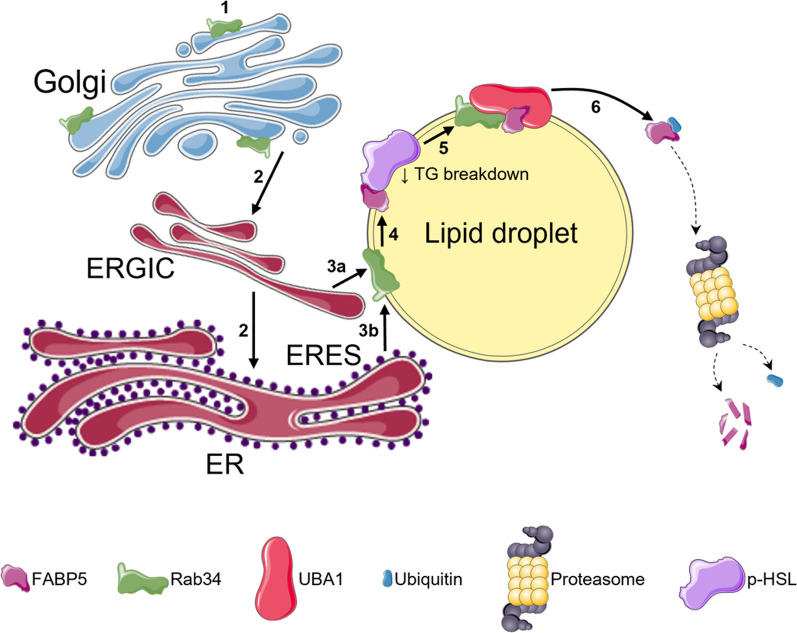
Schematic representation of the proposed trafficking route and mechanisms of action of Rab34 in adipocytes. Rab34 is located at the Golgi apparatus in preadipocytes (**1**) and transferred to the LD surface upon LD biogenesis. Specifically, Rab34 is transported from the Golgi to ER-related compartments via COPI-mediated retrograde transport pathway (**2**) and targeted to the LD surface via ERGIC (**3a**) and/or ERES (**3b**). Once at the LDs, Rab34 could bind FABP5 (**4**) and recruit UBA1 (**5**), which would promote ubiquitination and proteasomal degradation of FABP5 (**6**). FABP5 clearance from LDs would prevent full activation of phosphorylated HSL (p-HSL) thus reducing lipolysis, which would ultimately result in increased lipid accumulation in LDs. The figure was partly generated using Servier Medical Art, provided by Servier, licensed under a Creative Commons Attribution 3.0 unported license

A relevant feature of Rab34 is that it transits from the Golgi apparatus to the LDs in a differentiation-dependent manner, i.e., when LDs appeared in 3T3-L1 preadipocytes (D3). This characteristic has enabled us to unveil the trafficking route followed by Rab34 to reach the LDs (Fig. 10). According to our double-staining studies in differentiating 3T3-L1 adipocytes, this route would include Rab34 retrograde transport from the Golgi to ER-related compartments at early differentiation stages followed by transfer to LDs (from D3 onwards). Our confocal microscopy studies showing high colocalization rates between Rab34 and ARF1 at early stages of differentiation suggest that the first phase could be mediated via the COPI retrograde transport pathway. This is supported by the results obtained using BFA, a fungal drug that blocks COPI vesicle formation by preventing the membrane association of ARF1 [43]. Thus, exposure of 3T3-L1 cells to BFA before LD formation (i.e., D2) significantly reduced ulterior Rab34 association with LDs, supporting a relationship between Rab34 retrograde transport and COPI trafficking.

Notably, most COPI has been located at the ERGIC and proposed to participate in the organization of this compartment [63, 64]. Moreover, a role for COPI in mediating protein traffic from the ERGIC to LDs has been proposed [42]. Our findings demonstrating an increase in the colocalization between Rab34 and ERGIC53 at D3 of differentiation suggested a role for an ERGIC-to-LD route in Rab34 targeting LDs, at least at early stages of LD biogenesis. In this context, the ER-to-LD pathway represents a major route for protein targeting LDs [18]. Thus, it is accepted that LDs form and grow from the ER [65–67]. Upon budding, mature LDs remain connected to the ER membranes through membrane bridges [65–67]. In this way, proteins in ER membranes would reach the LD surface via lateral diffusion. Interestingly, recent studies in Drosophila cells have identified a late ER-to-LD targeting pathway that organizes at ERES and targets LD proteins, such as GPAT4 and other lipid-metabolizing enzymes [18]. In line with these findings, we observed that Rab34 increasingly colocalized with SEC16A as mature LDs accumulate in the cytoplasm of differentiating 3T3-L1 adipocytes, indicating that ERES may represent a pathway for Rab34 entrance into LDs. This late route could occur at the expense of Rab34 targeting LDs via ERGIC, as indicated by the progressive decrease in Rab34-ERGIC53 colocalization that parallels the increase in the colocalization between Rab34 and SEC16A. These results are in accordance with our data from primary human cells indicative of increased Rab34 localization to ERES over time in both OM and SC adipocytes. Notwithstanding this, our immunocytochemical results in freshly isolated mature adipocytes and in vitro differentiated adipocytes (OM and, especially, SC adipocytes) indicate that the ERGIC might also represent a pathway for late Rab34 targeting. Altogether, these results suggest the existence of two distinct sites for Rab34 transfer to LDs, ERGIC and ERES, whose relevance may be regulated in a differentiation- and depot-dependent manner. According to our current data in adipocytes cultured in 3D microgels mimicking the fibrotic microenvironment of the SC in the context of obesity [19], it is tempting to speculate that Rab34 traffic through ERES and/or ERGIC is disrupted under these conditions. Further studies are needed to fully establish the protein traffic routes to LDs in adipocytes as well as to unveil whether other obesity-associated insults may differentially regulate Rab34 distribution at ERGIC, ERES and LDs.

The observation that Rab34 localized to the Golgi apparatus in preadipocytes at early differentiation and, occasionally, in differentiated adipocytes (i.e., human primary OM adipocytes), as well as when exogenously expressed, prompted us to analyze the role of this GTPase at this compartment. Similar to that reported for other Rab proteins bound to Golgi membranes [45], exogenously expressed Rab34 induced Golgi ribbon fragmentation while Rab34 silencing had no effect on Golgi organization. The latter results indicate that Rab34 may be classified as a Golgi-associated Class 2 Rab protein, as opposed to Class 1 Rabs (i.e., Rab inactivation induces Golgi fragmentation) [45]. These findings led us to investigate whether Rab34 may also have a role in Golgi functions in adipocytes, which includes the regulation of adiponectin sorting and trafficking for secretion [68, 69]. In this line, we observed that Rab34 expression increased while Rab34 silencing decreased adiponectin secretion suggesting that, when associated with the Golgi, the GTPase may participate in adiponectin trafficking. This would be consistent with the role played by Rab34 in other cell types wherein this GTPase is also located at the Golgi and regulates the release of transport vesicles from the TGN to the secretory pathway [70]. Interestingly, our studies show that expression of Rab34 in preadipocytes also increased the amount of intracellular adiponectin multimers, which would be in agreement with the role played by the Golgi in adiponectin assembly into high order oligomers [71]. As regards to secretion, changes in Rab34 expression seemed to have an effect mainly on medium and, to a lesser extent, low molecular weight adiponectin isoforms. In all, our results suggest that Rab34, when located at the Golgi, regulates both cisternae integrity and adiponectin trafficking and oligomerization, suggesting the participation of this GTPase in the maintenance of Golgi organization and function. In all, our data suggest that Rab34 function(s) at the Golgi may be relevant for adipocytes at early stages of differentiation. As adipocytes differentiate, Rab34 action on adiponectin release would be replaced by other proteins participating in the secretory pathway. In this line, Rab11 and its downstream effectors, Rab11-Family of Interacting Proteins (FIPs), have been shown to regulate adiponectin trafficking and secretion from the endosomal compartment in differentiated 3T3-L1 adipocytes [72].

Our studies support the existence of differentiation-dependent signal(s) that trigger Rab34 dissociation from the Golgi and relocation to the LD surface. It seems likely that Rab34 association with each compartment is regulated by specific binding partners and/or regulators whose expression and/or availability at these locations may, in turn, be regulated during adipogenesis. Available evidence indicates that Rab membrane targeting is mainly determined by specific membrane-localized GEFs [49, 73]. In this scenario, the observation that the inactive (GDP-loaded) Rab34 mutant (T66N) associated with Golgi membranes indicates that Rab34 activation by Golgi GEF(s) is not a requisite for binding to this compartment. In contrast to the inactive mutant, the active (GTP-loaded) mutant (Q111L) was also bound to LDs, supporting a relationship between Rab34 activation and LD targeting. In its active form at LDs, Rab34 would interact with specific effectors at this cellular compartment. In accordance with our results, similar studies about the cellular distribution of active and inactive Rab34 isoforms in other cell types have shown that Rab34 localization is dependent on the activation status [32, 39–41]

Rab GTPase effectors comprise a variety of interacting proteins or protein complexes that convey Rab specific functions in a spatiotemporally regulated manner [73]. Regarding Rab34, this GTPase has been shown to interact with a Rab7 effector, Rab-interacting lysosomal protein (RILP), to regulate the spatial distribution of melanosomes and lysosomes in melanocytes and NRK cells, respectively [32, 74]. Though we cannot exclude the possibility that such interaction also occurs in adipocytes, neither BioID nor immunoprecipitation studies identified RILP as a potential Rab34 interacting protein in adipocytes. Indeed, Rab34 association with LDs together with the observation that Rab34 expression increased both LD size and intracellular lipid content while decreasing lipolysis, with these effects being counteracted by Rab34 down-regulation, suggested that the effectors conveying Rab34 actions on lipid homeostasis in adipocytes should be also related to LDs. Interestingly, neither Rab34 expression nor silencing modified the expression of key enzymes regulating lipogenesis, thus suggesting that Rab34-induced changes in lipid content at LDs may be related to the regulation of lipid hydrolysis. In this scenario, the fatty acid transporters, FABP4 and FABP5, with critical roles in regulating lipid transport [52, 75] as well as lipolysis through their interaction with HSL [52, 75], could act as Rab34 partners in adipocytes. Nevertheless, and although FABP4 also associates with LDs in adipocytes [19], we found no interaction between this chaperone and Rab34 in immunoprecipitation experiments. In line with our previous microscopic [26] and proteomic data [19], our current immunocytochemical studies demonstrated the association of FABP5 with the LD surface, wherein it colocalizes with Rab34. Moreover, both BioID and immunoprecipitation studies identified FABP5 as part of the Rab34 interactome, an observation that was further confirmed by pull-down experiments. To be more specific, our studies using Rab34 mutant variants indicated that Rab34-FABP5 interaction would occur when the GTPase is in its active GTP-bound form. In all, these results, together with our findings on the modulation of lipolysis by expression of either Rab34 or FABP5, and the blockade of FABP5-stimulated lipolysis when co-expressed with Rab34, supported a functional relationship between these two proteins. Notably, according to our data, this GTPase would exert an inhibitory effect on FABP5 function. The observation that Rab34 expression reduced FABP5 protein content (but not mRNA) indicated that this inhibition could be accounted for by an effect of the GTPase on FABP5 protein stability, which would be mediated through Rab34-induced ubiquitination and proteasomal degradation of FABP5. Notably, though Rab34 expression also reduced the protein content of the LD-associated lipase, ATGL, that may contribute to the net effect of the GTPase on lipid accumulation, this effect was not prevented by MG132 and a direct interaction between these two proteins could not be demonstrated. Likewise, a direct interaction between Rab34 and HSL was no detected in our immunoprecipitation studies. According to our studies, it is reasonable to propose that the LD-associated E1-ubiquitin ligase, UBA1, which colocalizes with Rab34 at the LD surface, acts as the Rab34 effector that conveys the action of the GTPase on FABP5 and, thus, on the regulation of lipolysis and lipid accumulation in adipocytes (Fig. 10). To the best of our knowledge, this is the first study reporting a potential role for a Rab protein in the regulation of protein ubiquitination and degradation. In the case of Rab34, this notion is reinforced by our studies demonstrating that this GTPase interacts not only with UBA1 but also with other LD proteins related to the ubiquitin system (i.e., UCHL3, ISG15). Whether Rab34-UBA1 action is exclusively exerted at the LDs or other cellular locations occupied by the GTPase (i.e., Golgi) remains to be investigated. In this line, Rab effectors are spatial and temporarily regulated [76] and thus, it seems plausible that the protein composition of the Rab34 interactome varies depending on the cellular compartment.

## Conclusions

Our studies indicate that Rab34 acts as a multipurpose, differentiation-dependent regulator of adipocyte functionality. Thus, Rab34 at the LDs would regulate TG lipolysis and lipid accumulation by directing FABP5 for destruction by the proteasome system. In addition, it may also control the oligomerization (i.e., biological activity) and secretion of a major adipokine with insulin-sensitizing actions, adiponectin. In this scenario, dysregulation of Rab34 levels and/or localization, as demonstrated herein for obesity conditions, could contribute, at least in part, to both the changes in the secretory profile and impaired lipid metabolism that characterize adipocyte dysfunction in obesity.

### Supplementary Information

**Additional file 1.** Clinical characteristics of the obese subjects included in the study.

**Additional file 2.** List of antibodies employed in this study.

**Additional file 3: Figure S1.** Localization of exogenously expressed Rab34 variants in 3T3-L1 adipocytes. **A** Colocalization study of endogenous Rab34 and GFP-Rab34 in 3T3-L1 cells. Representative confocal microscopy images of 3T3-L1 cells transfected with the GFP-Rab34 vector and stained with anti-Rab34 (red). **B**, **C** Representative confocal images of 3T3-L1 cells transfected with expression vectors coding for the constitutively active (GFP-Rab34-Q111L; **B**) or inactive (GFP-Rab34-T66N; **C**) Rab34 mutant proteins. The insets show high-magnification images of LDs. Scale bar: 10 μm. **Figure S2.** Validation of Rab34 expression and silencing experiments and characterization of Rab34 variants (related to Figure 5). **A** Quantitative immunoblotting analysis of Rab34 protein levels in 3T3-L1 cells expressing GFP-Rab34 or Rab34 siRNA (siRab34). Data are expressed as a percentage of values in control groups: GFP alone (Mock) or Scramble siRNA (Scr) (100%). **B** Histogram of percentage of LD size distribution in 3T3-L1 cells expressing Rab34 siRNA (siRab34) *vs.* scramble siRNA. **C** Representative immunoblots and quantification of Rab34 in rescue experiments. Cells were transfected with Rab34 siRNA (siRab34), alone or in combination with GFP-Rab34 (Rab34 recovery). At the end of the experiments, cells were processed for immunoblotting studies. **D**,** E** RT-qPCR analysis of ACSL1 and GPAT (**D**) or FASN mRNA (**E**) expression levels in 3T3-L1 cells expressing or silencing Rab34. ACSL1, GPAT and FASN mRNA levels were calculated using the Ct method and HPRT as the housekeeping gene. **F** Quantification of lipogenic and lipolytic activities in 3T3-L1 cells expressing wild-type Rab34 (WT), the constitutively active (Q111L) or the inactive (T66N) Rab34 variants. **G** Representative immunoblots and quantification of proteins related to lipid metabolism in 3T3-L1 cells expressing wild-type Rab34 (WT), the constitutively active (Q111L) or the inactive (T66N) Rab34 variants. **H** Co-immunoprecipitation experiments in HEK-293 AD cells expressing FABP5-c-Myc and either GFP-Rab34-WT, GFP-Rab34-Q111L or GFP-Rab34-T66N using anti-GFP beads. Both lysates and immunoprecipitates (IP) were subjected to immunoblotting with anti-GFP and anti-c-Myc antibodies. Data are referred to values in control cells expressing GFP alone (Mock) or Scramble siRNA (Scr) (100%), and expressed as mean ± SEM (n=3 biological replicates). *P<0.05; **P<0.01; ***P<0.001. **Figure S3.** FABP5 and HSL rescue experiments and analysis of Rab34 expression regulation by the proteasome (related to Fig. 7). **A** Representative immunoblots and quantification of protein extracts from 3T3-L1 cells transfected with expression vectors coding for GFP-Rab34 or FABP5-GFP, alone or in combination (FABP5 recovery group). **B** Representative immunoblots and quantification of protein extracts from 3T3-L1 cells transfected with Rab34 siRNA (siRab34) or GFP-HSL, alone or in combination (HSL recovery group). Data are expressed as a percentage of values in control groups (GFP alone, Mock; Scramble siRNA, Scr) (100%). **C**, **D** Quantification of Rab34 protein levels in 3T3-L1 cells expressing GFP-Rab34 (**C**), or Rab34 siRNA (siRab34) (**D**) and exposed or not (Basal) to MG132 (10 μmol/L, 12 h). Basal cells transfected with GFP alone (Mock) or scramble siRNA (Scr) were employed as controls. Graphs show the ratio of each immunosignal to β-actin immunosignal. Data are referred to values in control cells (Mock; Scr) (100%) and expressed as the mean ± SEM (n=3 biological replicates). *P<0.05; **P<0.01; ***P<0.001. **Figure S4.** Immunoblot quantifications of protein extracts from 3T3-L1 cells silenced for UBA1, and Rab34-FABP5/UBA1 colocalization in 3T3-L1/human cells (related to Fig. 8). Quantification of UBA1 (**A**), Rab34 (**B**), ATGL (**C**) and FABP5 (**D**) protein levels in 3T3-L1 cells transfected with UBA1 siRNA (siUBA1) or GFP-Rab34, alone or in combination. Cells transfected with scramble siRNA (Scr) were employed as controls. Graphs show the ratio of each immunosignal to β-actin immunosignal. Data are expressed as a percentage of values in control cells (Scr) (100%) and expressed as the mean ± SEM (n=3 biological replicates). **E** Representative confocal images of 3T3-L1 cells showing the colocalization (merge) of Rab34 (green) and UBA1 (red) during differentiation (days 0, 3, 6 and 10). Arrows indicate Rab34/UBA1 colocalization (yellow) at the LD surface. Manders’ coefficient between Rab34 and UBA1 was calculated to quantify the degree of colocalization between both signals. Data represent the mean ± SEM (n=6 cells/differentiation day, 2 biological replicates). **F** Representative confocal microscopy images of human OM and SC preadipocytes at different days of differentiation (day 5 and 10). Cells were incubated with the anti-Rab34 antibody and either antibodies against FABP5 or UBA1. Manders’ coefficients were calculated to assess the colocalization between signals. Data are expressed as the mean ± SEM (n=6 cells/differentiation day, 2 biological replicates). Scale bar: 10 μm. *P<0.05; **P<0.01; ***P<0.001. **Figure S5.** Rab34 expression levels in human adipose tissue and regulation of Rab34 in response to obesity insults. **A** Rab34 expression levels in subcutaneous (SC) adipose tissue samples from individuals with normal weight (lean; n=5) or obesity (n=12). The graph represents manually curated transcriptomic data from previously published works [1, 2]. ***P<0.001. **B** Analysis of Rab34 binding to LDs in 3T3-L1 adipocytes differentiated in 3D cultures mimicking adipose tissue fibrosis. Representative confocal images of 3T3-L1 cells grown on collagen I-based matrices in the absence (Control) or presence of lumican (30 ng/ml) (Lum) and immunostained for Rab34 (green) and counterstained with DAPI (blue) for nuclei identification. Morphometric analysis of the number of Rab34-labeled LDs and the intensity of Rab34 immunolabeling per cell were carried out using ImageJ. *P<0.05. Scale bar: 10 μm. **Figure S6.** Uncropped scans of all the western blots from Figs. 1, 2 and 4. The red dashed boxes indicate the regions of interest shown in the corresponding figures. Biological replicates in Fig. 1A were run in two gels. **Figure S7.** Uncropped scans of all the western blots from Fig. 5. The red dashed boxes indicate the regions of interest shown in the corresponding figure. **Figure S8.** Uncropped scans of western blots from Fig. 6 (part 1 of 4). The red dashed boxes indicate the regions of interest shown in the corresponding figure. **Figure S9.** Uncropped scans of western blots from Fig. 6 (part 2 of 4). The red dashed boxes indicate the regions of interest shown in the corresponding figure. **Figure S10.** Uncropped scans of western blots from Fig. 6 (part 3 of 4). The red dashed boxes indicate the regions of interest shown in the corresponding figure. **Figure S11.** Uncropped scans of western blots from Fig. 6 (part 4 of 4). The red dashed boxes indicate the regions of interest shown in the corresponding figure. **Figure S12.** Uncropped scans of all the western blots from Fig. 7. The red dashed boxes indicate the regions of interest shown in the corresponding figure. **Figure S13.** Uncropped scans of western blots from Fig. 8 (part 1 of 6). The red dashed boxes indicate the regions of interest shown in the corresponding figure. Biological replicates in Fig. 8A were run in three gels. **Figure S14.** Uncropped scans of western blots from Fig. 8 (part 2 of 6). **Figure S15.** Uncropped scans of western blots from Fig. 8 (part 3 of 6). **Figure S16.** Uncropped scans of western blots from Fig. 8 (part 4 of 6). The red dashed boxes indicate the regions of interest shown in the corresponding figure. The blue dashed boxes indicate the regions of interest shown in the corresponding figure when it was necessary to increase the exposure time to improve the band observation. **Figure S17.** Uncropped scans of western blots from Fig. 8 (part 5 of 6). The red dashed boxes indicate the regions of interest shown in the corresponding figure. The blue dashed boxes indicate the regions of interest shown in the corresponding figure when it was necessary to increase the exposure time to improve the band observation. **Figure S18.** Uncropped scans of western blots from Figs. 8 (part 6 of 6) and 9. The red dashed boxes indicate the regions of interest shown in the corresponding figure. Biological replicates in Fig. 8C were run in three gels. **Figure S19.** Uncropped scans of western blots from Figures S2A, S2C and S2G. The red dashed boxes indicate the regions of interest shown in the corresponding figure. **Figure S20.** Uncropped scans of western blots from Figure S2H. The red dashed boxes indicate the regions of interest shown in the corresponding figure. Biological replicates were run in three gels. **Figure S21.** Uncropped scans of western blots from Figures S3. The red dashed boxes indicate the regions of interest shown in the corresponding figure.

**Additional file 4. **Proteins found (352) in our BioID of c-Myc-BirA-Rab34 study.

**Additional file 5. **Proteins found (20) in our Bio-ID of Rab34 study, belonging to the “Metabolism of lipids” pathway of Reactome database.

**Additional file 6. **Proteins found (7) in our BioID of c-Myc-BirA-Rab34 study, belonging to the “Protein ubiquitination” and “Deubiquitination” pathways of Reactome database.

**Additional file 7. **Raw Western blot data of Fig. 1.

## Data Availability

Uncropped scans of all western blots and raw data used to generate the western blot graphs are included in Additional file 3: Figs. S6–S21 and Additional file 7, respectively. Any additional information required to reanalyze the data reported in this paper is available from the lead contact upon request.

## Abbreviations

3D: Three-dimensional
ACSL1: Acyl-CoA Synthetase Long Chain Family Member 1
ARF1: ADP-Ribosylation Factor 1
ATCC: American Type Culture Collection
ATGL: Adipose Triglyceride Lipase
BFA: Brefeldin A
BioID: Proximity-dependent biotin identification
BMI: Body Mass Index
CIDE: Cell death-inducing DFFA-like effector
COL-I: Collagen type I
COPI: Coat protein complex I
COPII: Coat protein complex II
D0-D10: Day 0 to Day 10
DAPI: 4’, 6-Diamidino-2-phenylindole
DMEM: Dulbecco’s modified Eagle’s medium
DUB: Deubiquitinating enzyme
ER: Endoplasmic Reticulum
ERES: ER Exit Sites
ERGIC: ER-Golgi Intermediate Compartment
FABP4: Fatty Acid Binding Protein 4
FABP5: Fatty Acid Binding Protein 5
FASN: Fatty Acid Synthase
FBS: Fetal Bovine Serum
FIP: Rab11-Family of Interacting Protein
FDR: False Discovery Rate
GAP: GTPase-activating proteins
GEF: Guanine nucleotide exchange factors
GPAT: Glycerol-3-Phosphate Acyltransferase
HA: Hemagglutinin
HFD: High-fat diet
HPRT: Hypoxanthine–guanine Phosphoribosyltransferase
HSL: Hormone-Sensitive Lipase
ISG15: ISG15 Ubiquitin Like Modifier
iST: In-Stage-Tip kit
LC–MS/MS: Liquid Chromatography-Tandem Mass Spectrometry
LD: Lipid Droplet
NCS: New-born Calf Serum
OM: Omental adipose tissue
PBS: Phosphate-buffered saline
PFA: Paraformaldehyde
PLIN: Perilipin
RILP: Rab-Interacting Lysosomal Protein
RIPA: Radioimmunoprecipitation Assay buffer
SC: Subcutaneous adipose tissue
SNARE: Soluble N-ethylmaleimide-sensitive factor Attachment proteins Receptor
STD: Standard laboratory diet
STX6: Syntaxin-6
SVF: Stromal-vascular fraction
TG: Triacylglycerol
TGN: Trans-Golgi Network
TripleTOF: Triple quadrupole Time-Of-Flight
UB: Ubiquitinating enzyme
UBA1: Ubiquitin-like modifier Activating enzyme 1
UCHL3: Ubiquitin C-Terminal Hydrolase L3
WAT: White adipose tissue
WT: Wild-type
