# Supplementary material for: Localization, traffic and function of Rab34 in adipocyte lipid and endocrine functions
Source: J Biomed Sci. 2024 Jan 5;31:2. doi: 10.1186/s12929-023-00990-8 (PMC10770960; doi:10.1186/s12929-023-00990-8)
Supplement: Supplementary file 1 — Additional file 1. Clinical characteristics of the obese subjects included in the study. [file 12929_2023_990_MOESM1_ESM.docx]

**Additional file 1.** Clinical characteristics of the obese subjects included in the study.

|  |  |
| --- | --- |
| n | 9 |
| Sex (male/female) | 3/6 |
| Age (years) | 49 ± 2 |
| Weight (kg) | 133.68 ± 7.97 |
| Height (m) | 1.68 ± 0.04 |
|  |  |
| BMI (kg/m^2^) | 47.24 ± 1.53 |
| Body fat (%) | 62.34 ± 2.17 |
|  |  |
| Glucose (mg/dL) | 100.56 ± 12.96 |
| HbA1c (%) | 5.57 ± 0.12 |
| Triglycerides (mg/dL) | 124.56 ± 18.72 |
| Total cholesterol (mg/dL) | 144.00 ± 11.46 |
| LDL-cholesterol (mg/dL) | 76.50 ± 8.62 |
| HDL-cholesterol (mg/dL) | 38.63 ± 1.77 |
| CRP (mg/L) | 8.49 ± 2.48 |
| AST (IU/L) | 23.63 ± 2.40 |
| ALT (IU/L) | 30.63 ± 6.61 |
| Ferritin (ng/ml) | 144.20 ± 34.18 |
| Hematocrit (%) | 40.92 ± 1.63 |
| Haematids [million/mm^3^] | 4.62 ± 0.19 |
| Platelets [million/mm^3^] | 0.24 ± 0.02 |

Data presented as mean ± SEM. AST, aspartate aminotransferase; ALT, alanine aminotransferase; CRP, high-sensitivity C-reactive protein.
