# Supplementary material for: Localization, traffic and function of Rab34 in adipocyte lipid and endocrine functions
Source: J Biomed Sci. 2024 Jan 5;31:2. doi: 10.1186/s12929-023-00990-8 (PMC10770960; doi:10.1186/s12929-023-00990-8)
Supplement: Supplementary file 2 — Additional file 2. List of antibodies employed in this study. [file 12929_2023_990_MOESM2_ESM.doc]

**Additional file 2. List of antibodies employed in this study.**

| Antibody | Species | Dilution | Supplier | Reference |
| --- | --- | --- | --- | --- |
|  |  |  |  |  |
| **Western blot and/or**  **Co-immunoprecipitation** |  |  |  |  |
| Rab34 | Rabbit | 1:2000 | Abcam, Cambridge, UK | ab73383 |
| β-Actin | Mouse | 1:10000 | Sigma-Aldrich, Madrid, Spain | A2066 |
| Adiponectin | Mouse | 1:1000 | Novus Biologicals, Littleton, CO, USA | NBP2-22450 |
| PPAR-γ | Rabbit | 1:1000 | Cell Signaling, Danvers, MS, USA | 2443 |
| PLIN2 | Mouse | 1:200 | Santa Cruz Biotechnology, Heilderberg, Germany | sc-377429 |
| PLIN1 | Guinea pig | 1:2000 | Progen, Heidelberg, Germany | GP29 |
| DGAT2 | Goat | 1:1000 | Novus Biologicals, Littleton, CO, USA | NB100-57851 |
| HSL | Rabbit | 1:1000 | Abcam, Cambridge, UK | ab45422 |
| p-HSL | Rabbit | 1:750 | Cell Signaling, Danvers, MS, USA | 4139 |
| ATGL | Rabbit | 1:1000 | Wuhan Huamei Biotech. Co. Ltd., Huamei, China | WH180241 |
| CGI58 | Goat | 1:1000 | Abcam, Cambridge, UK | ab59488 |
| FABP4 | Rabbit | 1:1000 | Cell Signaling, Danvers, MS, USA | 3544 |
| FABP5 | Rabbit | 1:1000 | Cell Signaling, Danvers, MS, USA | 39926 |
| c-Myc | Rabbit | 1:1000 | Cell Signaling, MA, USA | 2278 |
| GFP | Rabbit | 1:1000 | Abcam, Cambridge, UK | ab290 |
| UBA1 | Mouse | 1:5000 | Invitrogen, Carlsbad, CA, USA | 67198-1-IG |
| HA | Rat | 1:1000 | Sigma-Aldrich, Madrid, Spain | 11867423001 |
| Peroxidase-conjugated secondary antibody | Goat | 1:2500 | Jackson ImmunoResearch, West Grove, PA, USA | 111-035-144 |
| Peroxidase-conjugated secondary antibody | Rabbit | 1:2500 | Sigma-Aldrich, Madrid, Spain | A9044 |
| Peroxidase-conjugated secondary antibody | Goat | 1:2500 | Sigma-Aldrich, Madrid, Spain | A7289 |
| Peroxidase-conjugated secondary antibody | Mouse | 1:2500 | Sigma-Aldrich, Madrid, Spain | A9452 |
| Peroxidase-conjugated secondary antibody | Goat | 1:10000 | Jackson ImmunoResearch, West Grove, PA, USA | 112-035-062 |
| **Immunocytochemistry** |  |  |  |  |
| Rab34 | Rabbit | 1:500 | Abcam, Cambridge, UK | ab73383 |
| GM130 | Mouse | 1:500 | BD Biosciences, Bedford, MA, USA | 610822 |
| Perilipin-1 | Guinea pig | 1:1000 | Progen, Heidelberg, Germany | GP29 |
| STX6 | Mouse | 1:500 | BD Biosciences, Bedford, MA, USA | 610635 |
| ARF1 | Mouse | 1:500 | Abcam, Cambridge, UK | ab2806 |
| ERGIC53 | Mouse | 1:100 | Santa Cruz Biotechnology, Heilderberg, Germany | sc-398777 |
| SEC23 | Mouse | 1:100 | Santa Cruz Biotechnology, Heilderberg, Germany | sc-12107 |
| SEC16A | Rabbit | 1:50 | Sigma-Aldrich, Madrid, Spain | HPA005684 |
| FABP5 | Mouse | 1:500 | Santa Cruz Biotechnology, Heilderberg, Germany | sc-365166 |
| UBA1 | Mouse | 1:500 | Invitrogen, Carlsbad, CA, USA | 67198-1-IG |
| Alexa Fluor^TM^ 594-conjugated secondary antibody | Donkey | 1:500 | Invitrogen, Carlsbad, CA, USA | A-21207 |
| Alexa Fluor^TM^ 594-conjugated secondary antibody | Goat | 1:500 | Invitrogen, Carlsbad, CA, USA | A-11076 |
| Alexa Fluor^TM^ 594-conjugated secondary antibody | Chicken | 1:500 | Invitrogen, Carlsbad, CA, USA | A-21201 |
| Alexa Fluor^TM^ 488-conjugated secondary antibody | Chicken | 1:500 | Invitrogen, Carlsbad, CA, USA | A-21200 |
| Alexa Fluor^TM^ 488-conjugated secondary antibody | Donkey | 1:500 | Invitrogen, Carlsbad, CA, USA | A-21206 |
| Alexa Fluor^TM^ 405-conjugated secondary antibody | Goat | 1:500 | Abcam, Cambridge, UK | ab175678 |

References and optimized concentrations of the antibodies used in each experiment. Peroxisome Proliferator Activated Receptor Gamma (PPAR-γ). Perilipin-2 (PLIN2). Perilipin-1 (PLIN1). Diacylglycerol O-Acyltransferase 2 (DGAT2). Hormone-Sensitive Lipase (HSL). Comparative gene identification-58 (CGI58). Fatty acid-binding protein 4 (FABP4). Fatty acid-binding protein 5 (FABP5). Green Fluorescent Protein (GFP). Ubiquitin-like modifier activating enzyme 1 (UBA1). Hemagglutinin (HA). Cis-Golgi Matrix protein 130 (GM130). Syntaxin-6 (STX6). ADP-Ribosylation Factor 1 (ARF1). Endoplasmic Reticulum-Golgi Intermediate Compartment 53 kDa protein (ERGIC53).
