# Supplementary material for: Localization, traffic and function of Rab34 in adipocyte lipid and endocrine functions
Source: J Biomed Sci. 2024 Jan 5;31:2. doi: 10.1186/s12929-023-00990-8 (PMC10770960; doi:10.1186/s12929-023-00990-8)
Supplement: Supplementary file 3 — Additional file 3: Figure S1. Localization of exogenously expressed Rab34 variants in 3T3-L1 adipocytes. A Colocalization study of endogenous Rab34 and GFP-Rab34 in 3T3-L1 cells. Representative confocal microscopy images of 3T3-L1 cells transfected with the GFP-Rab34 vector and stained with anti-Rab34 (red). B, C Representative confocal images of 3T3-L1 cells transfected with expression vectors coding for the constitutively active (GFP-Rab34-Q111L; B) or inactive (GFP-Rab34-T66N; C) Rab34 mutant proteins. The insets show high-magnification images of LDs. Scale bar: 10 μm. Figure S2. Validation of Rab34 expression and silencing experiments and characterization of Rab34 variants (related to Figure 5). A Quantitative immunoblotting analysis of Rab34 protein levels in 3T3-L1 cells expressing GFP-Rab34 or Rab34 siRNA (siRab34). Data are expressed as a percentage of values in control groups: GFP alone (Mock) or Scramble siRNA (Scr) (100%). B Histogram of percentage of LD size distribution in 3T3-L1 cells expressing Rab34 siRNA (siRab34) vs. scramble siRNA. C Representative immunoblots and quantification of Rab34 in rescue experiments. Cells were transfected with Rab34 siRNA (siRab34), alone or in combination with GFP-Rab34 (Rab34 recovery). At the end of the experiments, cells were processed for immunoblotting studies. D, E RT-qPCR analysis of ACSL1 and GPAT (D) or FASN mRNA (E) expression levels in 3T3-L1 cells expressing or silencing Rab34. ACSL1, GPAT and FASN mRNA levels were calculated using the Ct method and HPRT as the housekeeping gene. F Quantification of lipogenic and lipolytic activities in 3T3-L1 cells expressing wild-type Rab34 (WT), the constitutively active (Q111L) or the inactive (T66N) Rab34 variants. G Representative immunoblots and quantification of proteins related to lipid metabolism in 3T3-L1 cells expressing wild-type Rab34 (WT), the constitutively active (Q111L) or the inactive (T66N) Rab34 variants. H Co-immunoprecipitation experiments in HEK-293 [file 12929_2023_990_MOESM3_ESM.docx]

**Additional File 3**

**Localization, traffic and function of Rab34 in adipocyte lipid and endocrine functions**

**Jaime López-Alcalá^1^, Ana Gordon^1^*, Andrés Trávez^1^, Carmen Tercero-Alcázar^1^, Alejandro Correa-Sáez^1^, María Jesús González-Rellán^2,3^, Oriol A. Rangel-Zúñiga^2,4^, Amaia Rodríguez^2,5^, Antonio Membrives^6^, Gema Frühbeck^2,5^, Rubén Nogueiras^2,3^, Marco A. Calzado^1^, Rocío Guzmán-Ruiz^1,2^ and María M. Malagón^1,2^***

^1^Department of Cell Biology, Physiology, and Immunology, Maimonides Institute for Biomedical Research of Córdoba (IMIBIC)/University of Córdoba (UCO)/Reina Sofía University Hospital (HURS), Córdoba, Spain

^2^CIBER Physiopathology of Obesity and Nutrition (CIBERobn), ISCIII, Spain

^3^Department of Physiology, CiMUS, University of Santiago de Compostela-Instituto de Investigación Sanitaria, Santiago de Compostela, Spain

^4^Lipids and Atherosclerosis Unit, IMIBIC/University of Córdoba (UCO)/Reina Sofía University Hospital (HURS), Córdoba, Spain

^5^Metabolic Research Laboratory, Department of Endocrinology & Nutrition/Clinic University of Navarra, IdiSNA, Pamplona, Spain.

^6^Department of Medical-Surgical Specialties/ University of Córdoba (UCO)/Reina Sofia University Hospital (HURS), Córdoba, Spain

**
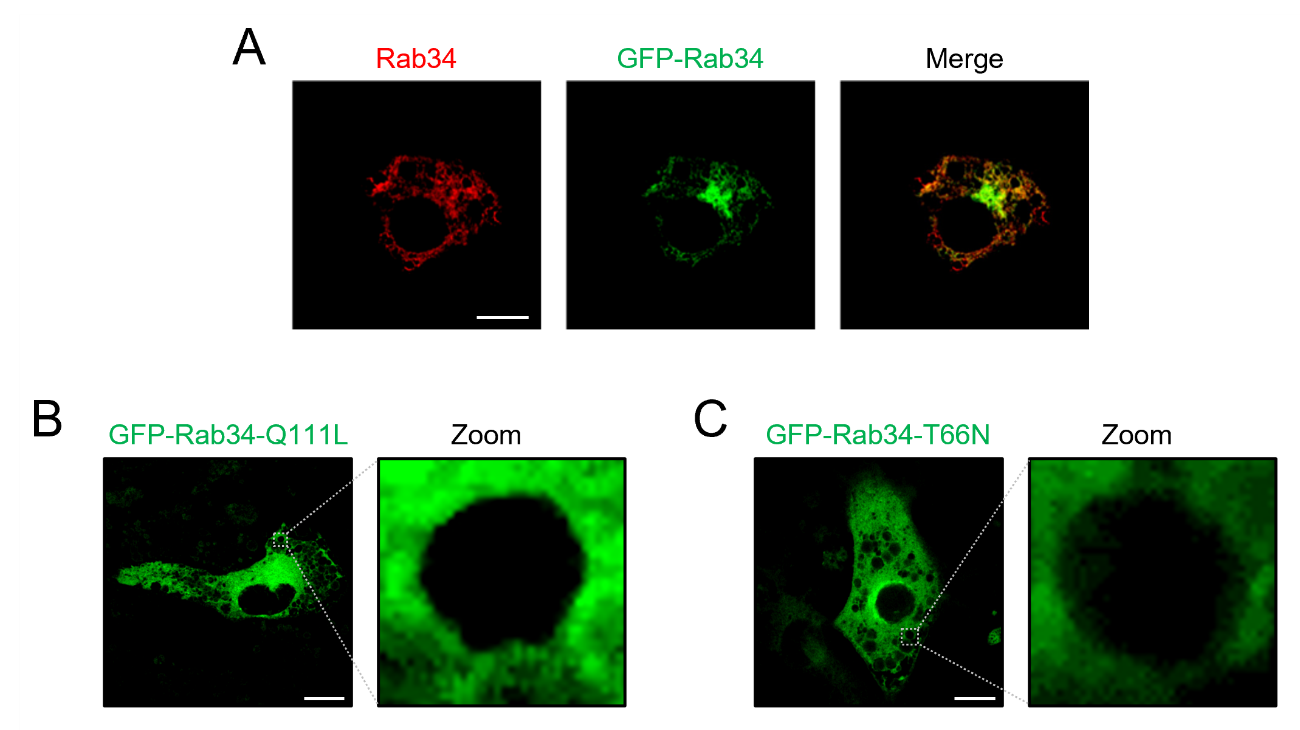
**

**Additional file 3: Fig. S1. Localization of exogenously expressed Rab34 variants in 3T3-L1 adipocytes.** (**A**) Colocalization study of endogenous Rab34 and GFP-Rab34 in 3T3-L1 cells. Representative confocal microscopy images of 3T3-L1 cells transfected with the GFP-Rab34 vector and stained with anti-Rab34 (red). (**B**-**C**) Representative confocal images of 3T3-L1 cells transfected with expression vectors coding for the constitutively active (GFP-Rab34-Q111L; **B**) or inactive (GFP-Rab34-T66N; **C**) Rab34 mutant proteins. The insets show high-magnification images of LDs. Scale bar: 10 μm.


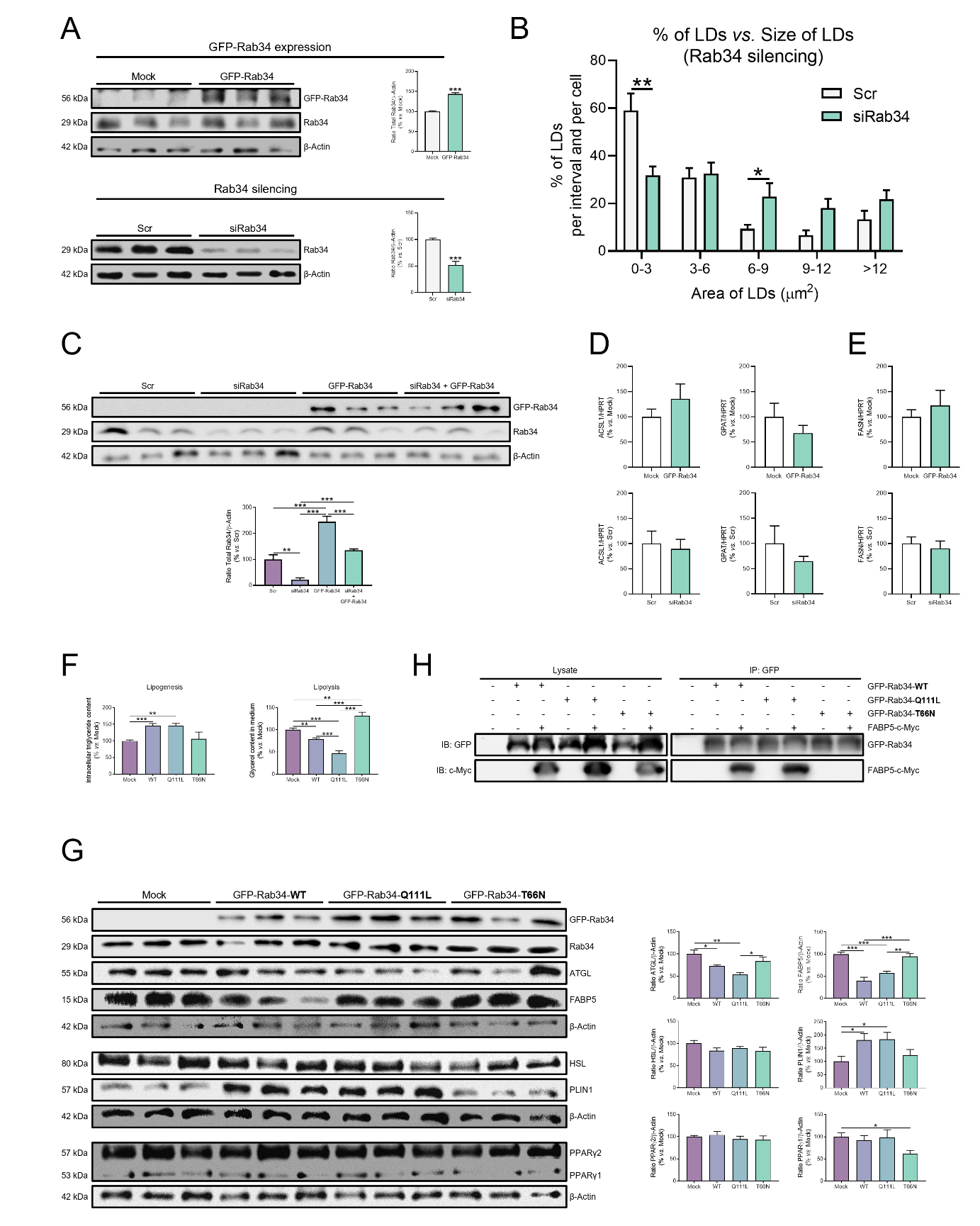


**Additional file 3: Fig. S2. Validation of Rab34 expression and silencing experiments and characterization of Rab34 variants (related to Fig. 5).** (**A**) Quantitative immunoblotting analysis of Rab34 protein levels in 3T3-L1 cells expressing GFP-Rab34 or Rab34 siRNA (siRab34). Data are expressed as a percentage of values in control groups: GFP alone (Mock) or Scramble siRNA (Scr) (100%). (**B**) Histogram of percentage of LD size distribution in 3T3-L1 cells expressing Rab34 siRNA (siRab34) *vs.* scramble siRNA. (**C**) Representative immunoblots and quantification of Rab34 in rescue experiments. Cells were transfected with Rab34 siRNA (siRab34), alone or in combination with GFP-Rab34 (Rab34 recovery). At the end of the experiments, cells were processed for immunoblotting studies. (**D**, **E**) RT-qPCR analysis of ACSL1 and GPAT (**D**) or FASN mRNA (**E**) expression levels in 3T3-L1 cells expressing or silencing Rab34. ACSL1, GPAT and FASN mRNA levels were calculated using the Ct method and HPRT as the housekeeping gene. (**F**) Quantification of lipogenic and lipolytic activities in 3T3-L1 cells expressing wild-type Rab34 (WT), the constitutively active (Q111L) or the inactive (T66N) Rab34 variants. (**G**) Representative immunoblots and quantification of proteins related to lipid metabolism in 3T3-L1 cells expressing wild-type Rab34 (WT), the constitutively active (Q111L) or the inactive (T66N) Rab34 variants. (**H**) Co-immunoprecipitation experiments in HEK-293 AD cells expressing FABP5-c-Myc and either GFP-Rab34-WT, GFP-Rab34-Q111L or GFP-Rab34-T66N using anti-GFP beads. Both lysates and immunoprecipitates (IP) were subjected to immunoblotting with anti-GFP and anti-c-Myc antibodies. Data are referred to values in control cells expressing GFP alone (Mock) or Scramble siRNA (Scr) (100%), and expressed as mean ± SEM (n=3 biological replicates). *, P<0.05; **, P<0.01; ***, P<0.001.


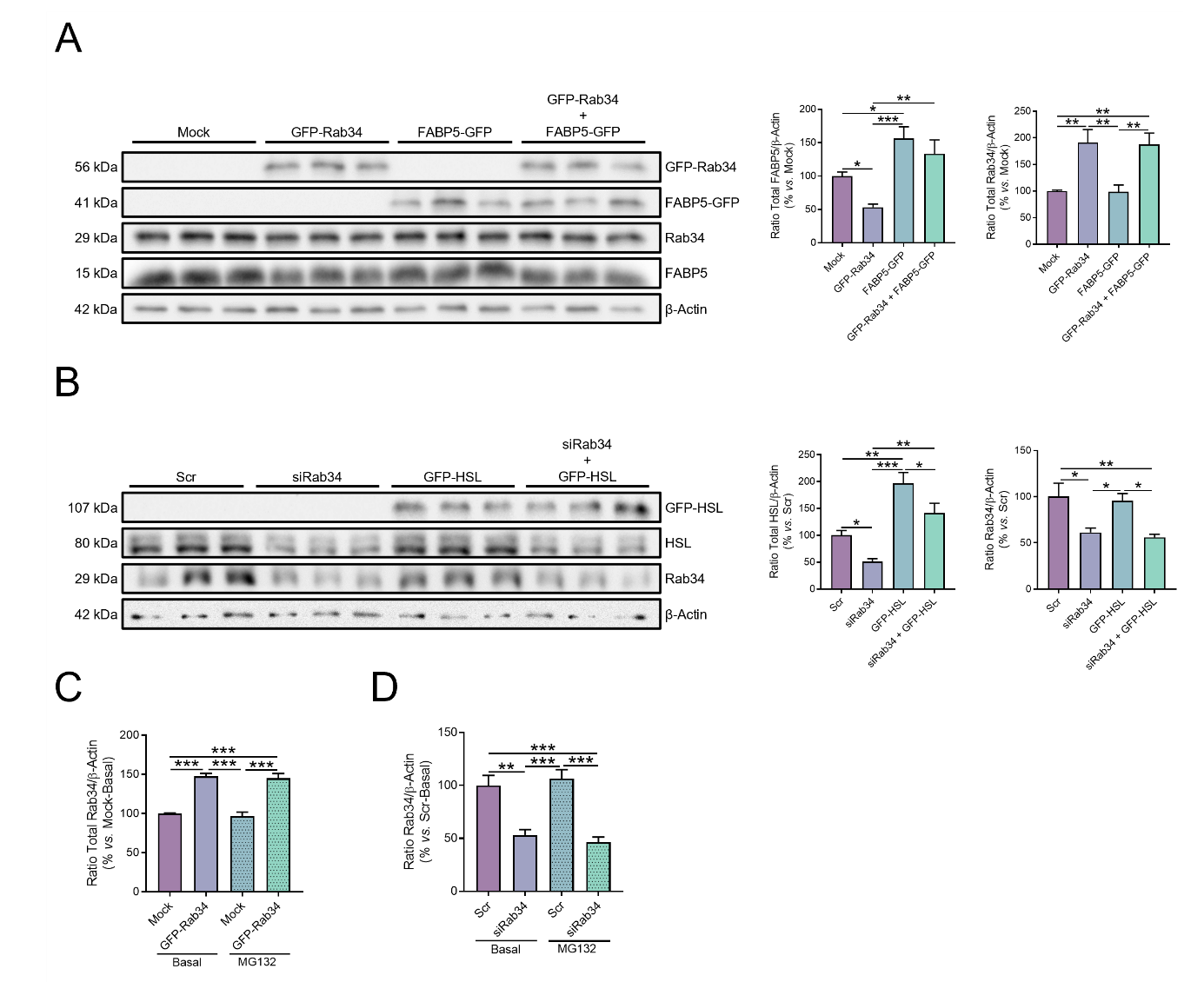


**Additional file 3: Fig. S3. FABP5 and HSL rescue experiments and analysis of Rab34 expression regulation by the proteasome (related to Fig. 7).** (**A**) Representative immunoblots and quantification of protein extracts from 3T3-L1 cells transfected with expression vectors coding for GFP-Rab34 or FABP5-GFP, alone or in combination (FABP5 recovery group). (**B**) Representative immunoblots and quantification of protein extracts from 3T3-L1 cells transfected with Rab34 siRNA (siRab34) or GFP-HSL, alone or in combination (HSL recovery group). Data are expressed as a percentage of values in control groups (GFP alone, Mock; Scramble siRNA, Scr) (100%). (**C**, **D**) Quantification of Rab34 protein levels in 3T3-L1 cells expressing GFP-Rab34 (**C**), or Rab34 siRNA (siRab34) (**D**) and exposed or not (Basal) to MG132 (10 μmol/L, 12 h). Basal cells transfected with GFP alone (Mock) or scramble siRNA (Scr) were employed as controls. Graphs show the ratio of each immunosignal to β-actin immunosignal. Data are referred to values in control cells (Mock; Scr) (100%) and expressed as the mean ± SEM (n=3 biological replicates). *, P<0.05; **, P<0.01; ***, P<0.001.


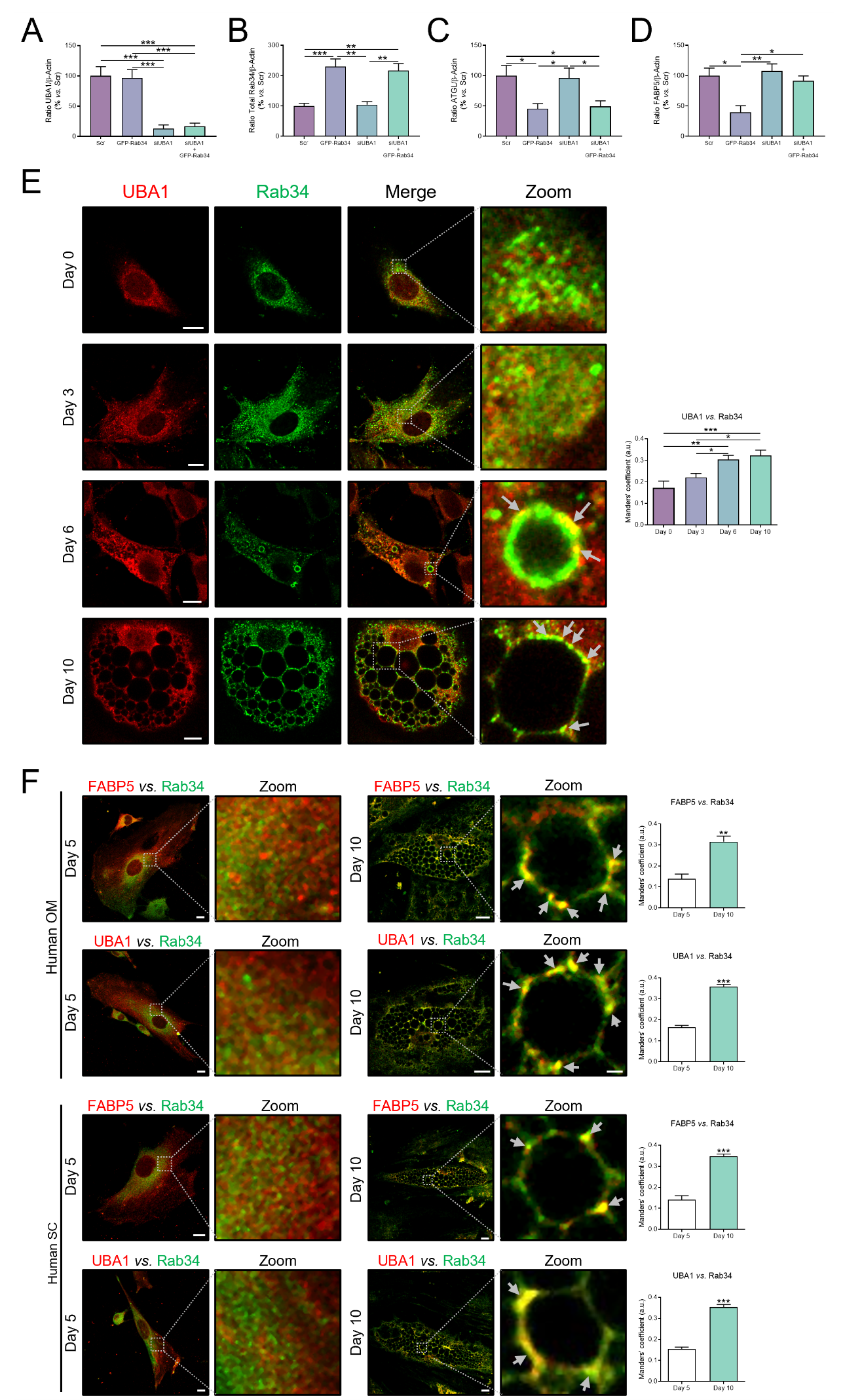


**Additional file 3: Fig. S4. Immunoblot quantifications of protein extracts from 3T3-L1 cells** **silenced for UBA1, and Rab34-FABP5/UBA1 colocalization in 3T3-L1/human cells (related to Fig. 8).** Quantification of UBA1 (**A**), Rab34 (**B**), ATGL (**C**) and FABP5 (**D**) protein levels in 3T3-L1 cells transfected with UBA1 siRNA (siUBA1) or GFP-Rab34, alone or in combination. Cells transfected with scramble siRNA (Scr) were employed as controls. Graphs show the ratio of each immunosignal to β-actin immunosignal. Data are expressed as a percentage of values in control cells (Scr) (100%) and expressed as the mean ± SEM (n=3 biological replicates). (**E**) Representative confocal images of 3T3-L1 cells showing the colocalization (merge) of Rab34 (green) and UBA1 (red) during differentiation (days 0, 3, 6 and 10). Arrows indicate Rab34/UBA1 colocalization (yellow) at the LD surface. Manders’ coefficient between Rab34 and UBA1 was calculated to quantify the degree of colocalization between both signals. Data represent the mean ± SEM (n=6 cells/differentiation day, 2 biological replicates). (**F**) Representative confocal microscopy images of human OM and SC preadipocytes at different days of differentiation (day 5 and 10). Cells were incubated with the anti-Rab34 antibody and either antibodies against FABP5 or UBA1. Manders’ coefficients were calculated to assess the colocalization between signals. Data are expressed as the mean ± SEM (n=6 cells/differentiation day, 2 biological replicates). Scale bar: 10 μm. *, P<0.05; **, P<0.01; ***, P<0.001.


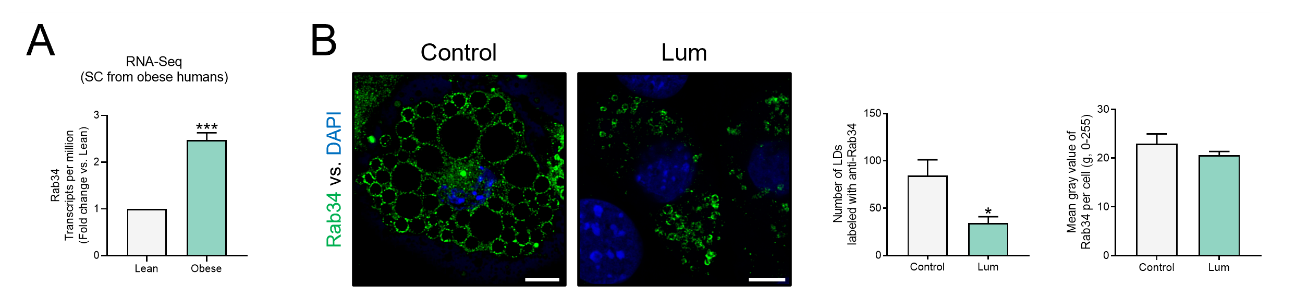


**Additional file 3: Fig. S5. Rab34 expression levels in human adipose tissue and regulation of Rab34 in response to obesity insults.** (**A**) Rab34 expression levels in subcutaneous (SC) adipose tissue samples from individuals with normal weight (lean; n=5) or obesity (n=12). The graph represents manually curated transcriptomic data from previously published works [1, 2]. ***, P<0.001. (**B**) Analysis of Rab34 binding to LDs in 3T3-L1 adipocytes differentiated in 3D cultures mimicking adipose tissue fibrosis. Representative confocal images of 3T3-L1 cells grown on collagen I-based matrices in the absence (Control) or presence of lumican (30 ng/ml) (Lum) and immunostained for Rab34 (green) and counterstained with DAPI (blue) for nuclei identification. Morphometric analysis of the number of Rab34-labeled LDs and the intensity of Rab34 immunolabeling per cell were carried out using ImageJ. *, P<0.05. Scale bar: 10 μm.


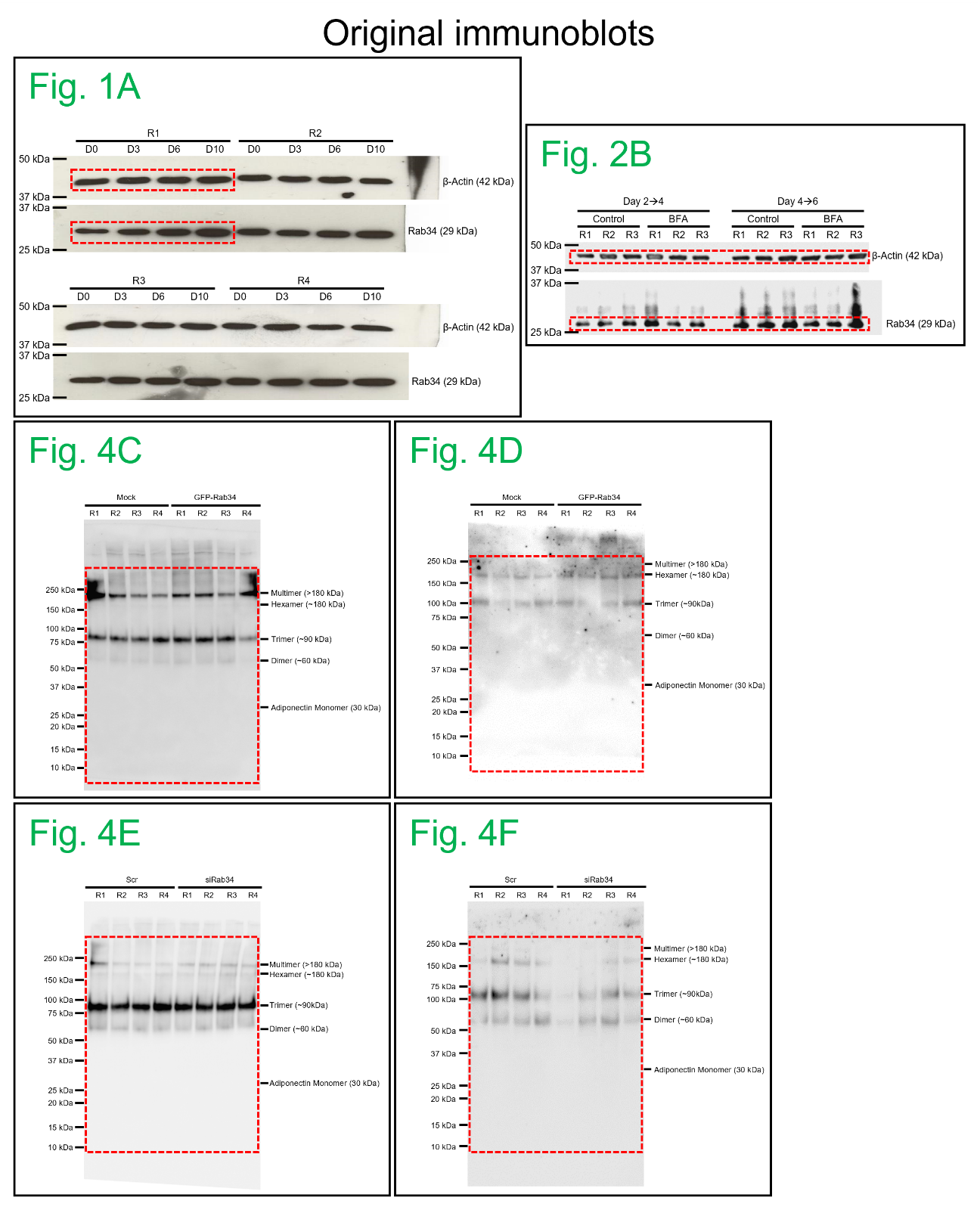


**Additional file 3: Fig. S6. Uncropped scans of all the western blots from Figures 1, 2 and 4.** The red dashed boxes indicate the regions of interest shown in the corresponding figures. Biological replicates in Figure 1A were run in two gels.


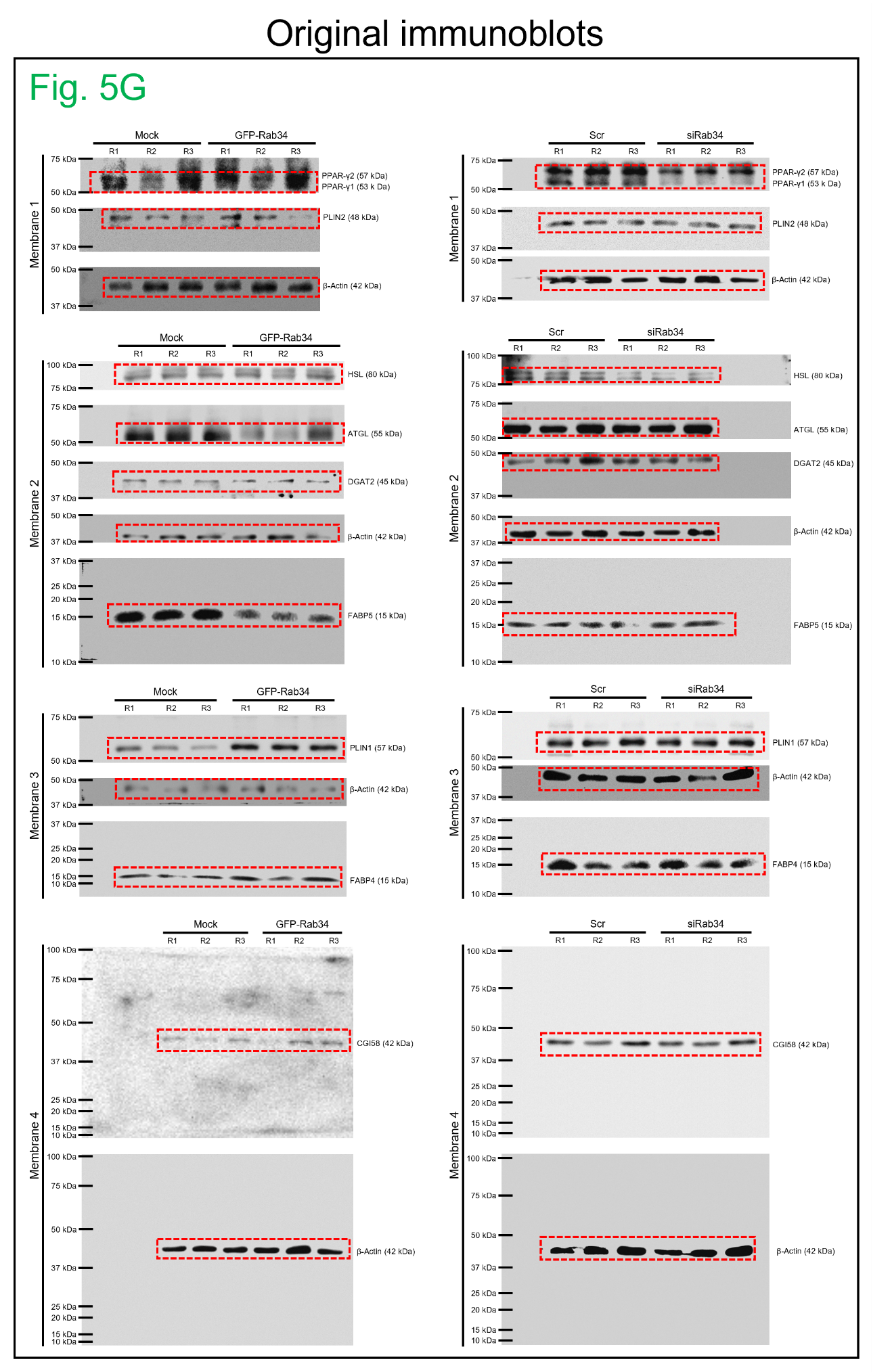


**Additional file 3: Fig. S7. Uncropped scans of all the western blots from Figure 5.** The red dashed boxes indicate the regions of interest shown in the corresponding figure.


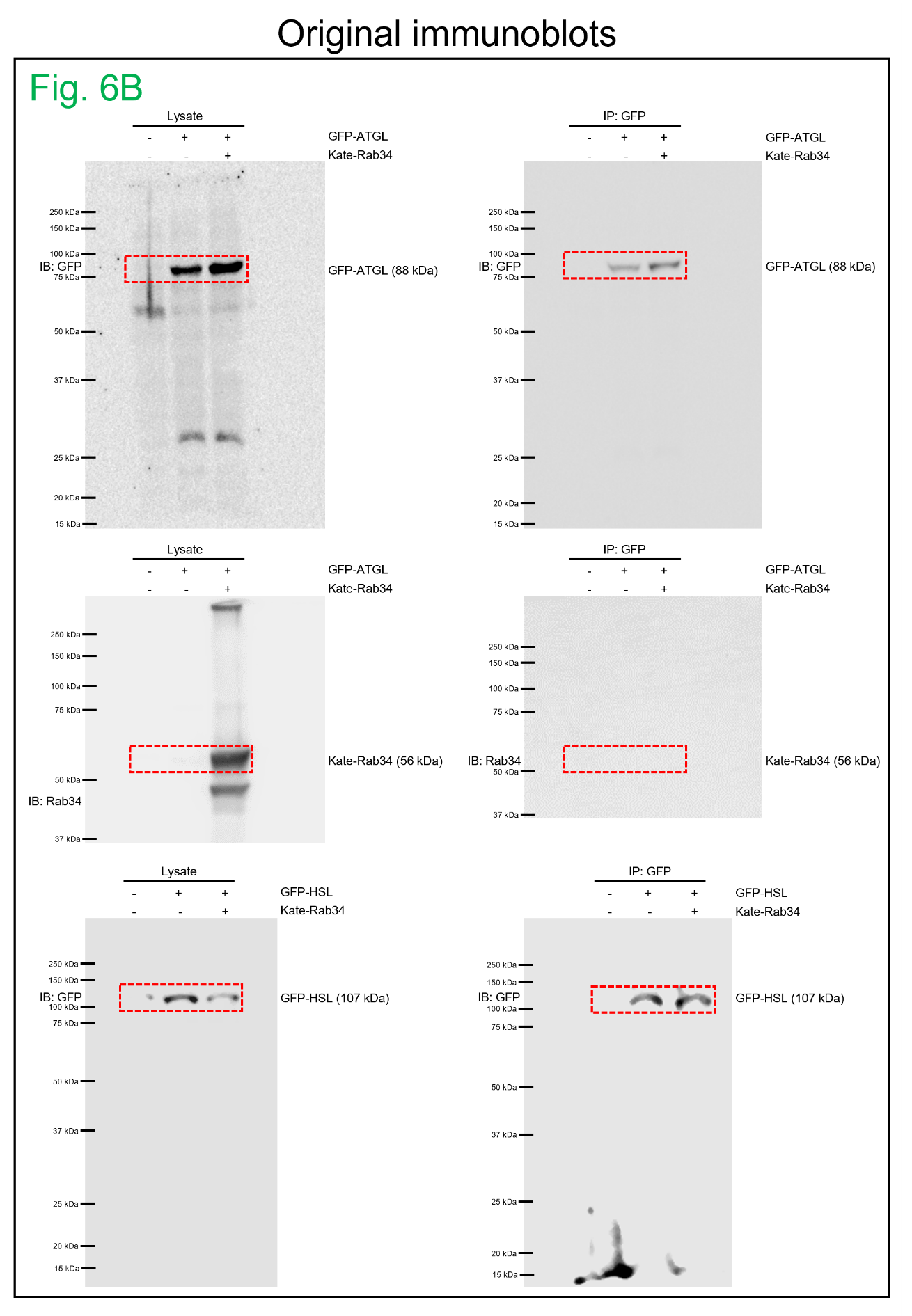


**Additional file 3: Fig. S8. Uncropped scans of western blots from Figure 6 (part 1 of 4).** The red dashed boxes indicate the regions of interest shown in the corresponding figure.


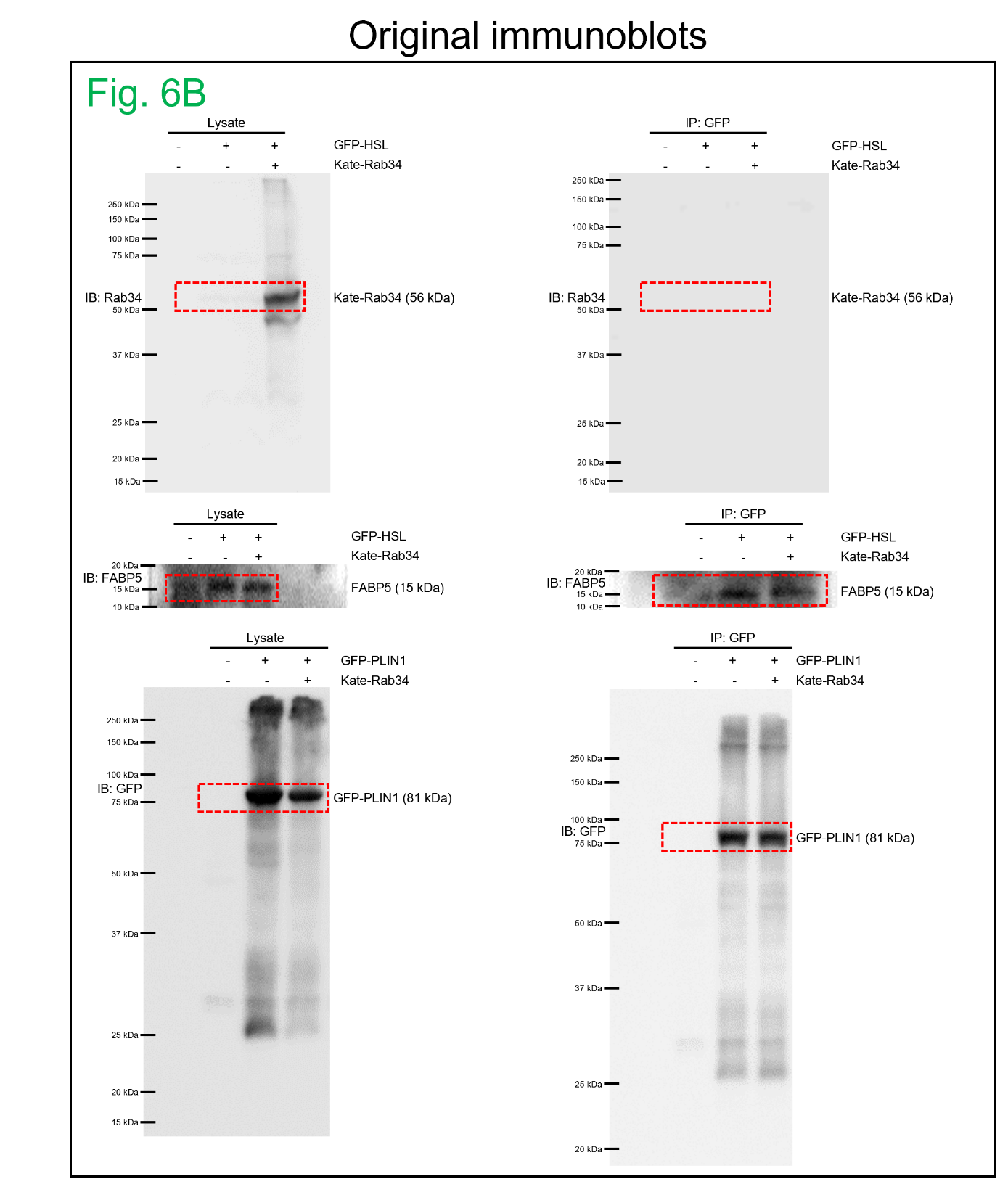


**Additional file 3: Fig. S9. Uncropped scans of western blots from Figure 6 (part 2 of 4).** The red dashed boxes indicate the regions of interest shown in the corresponding figure.


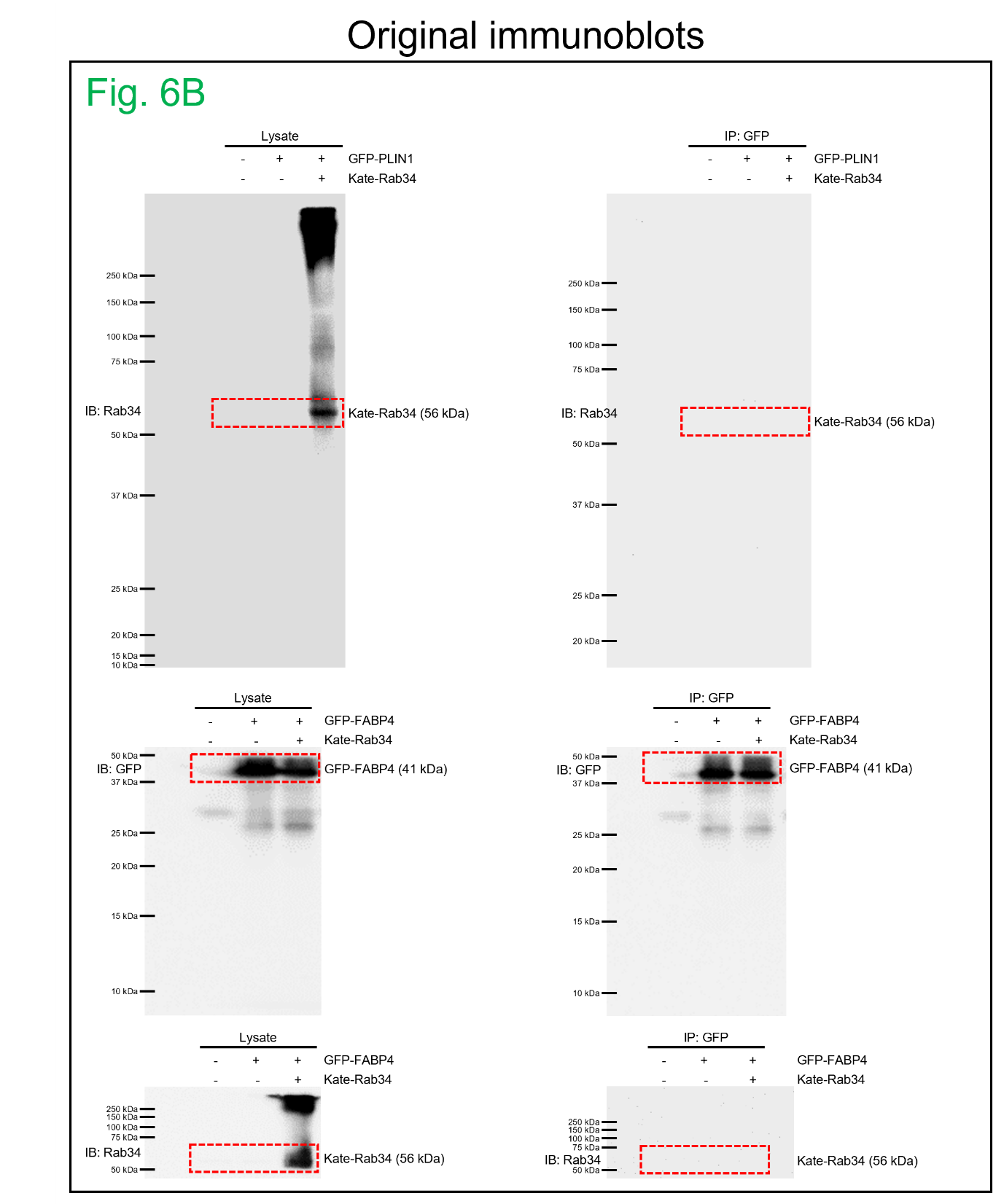


**Additional file 3:** **Fig. S10. Uncropped scans of western blots from Figure 6 (part 3 of 4).** The red dashed boxes indicate the regions of interest shown in the corresponding figure.


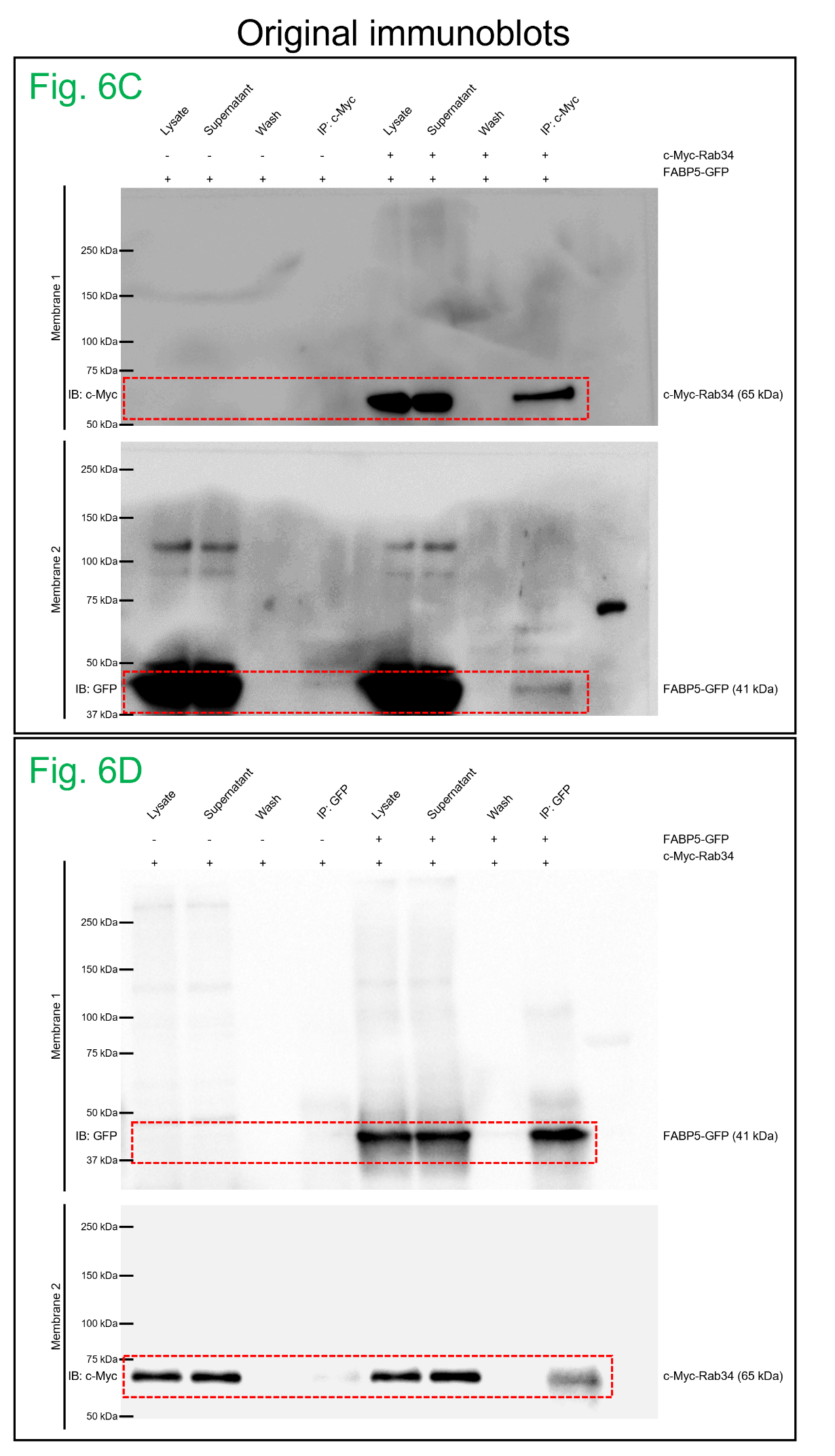


**Additional file 3: Fig. S11. Uncropped scans of western blots from Figure 6 (part 4 of 4).** The red dashed boxes indicate the regions of interest shown in the corresponding figure.


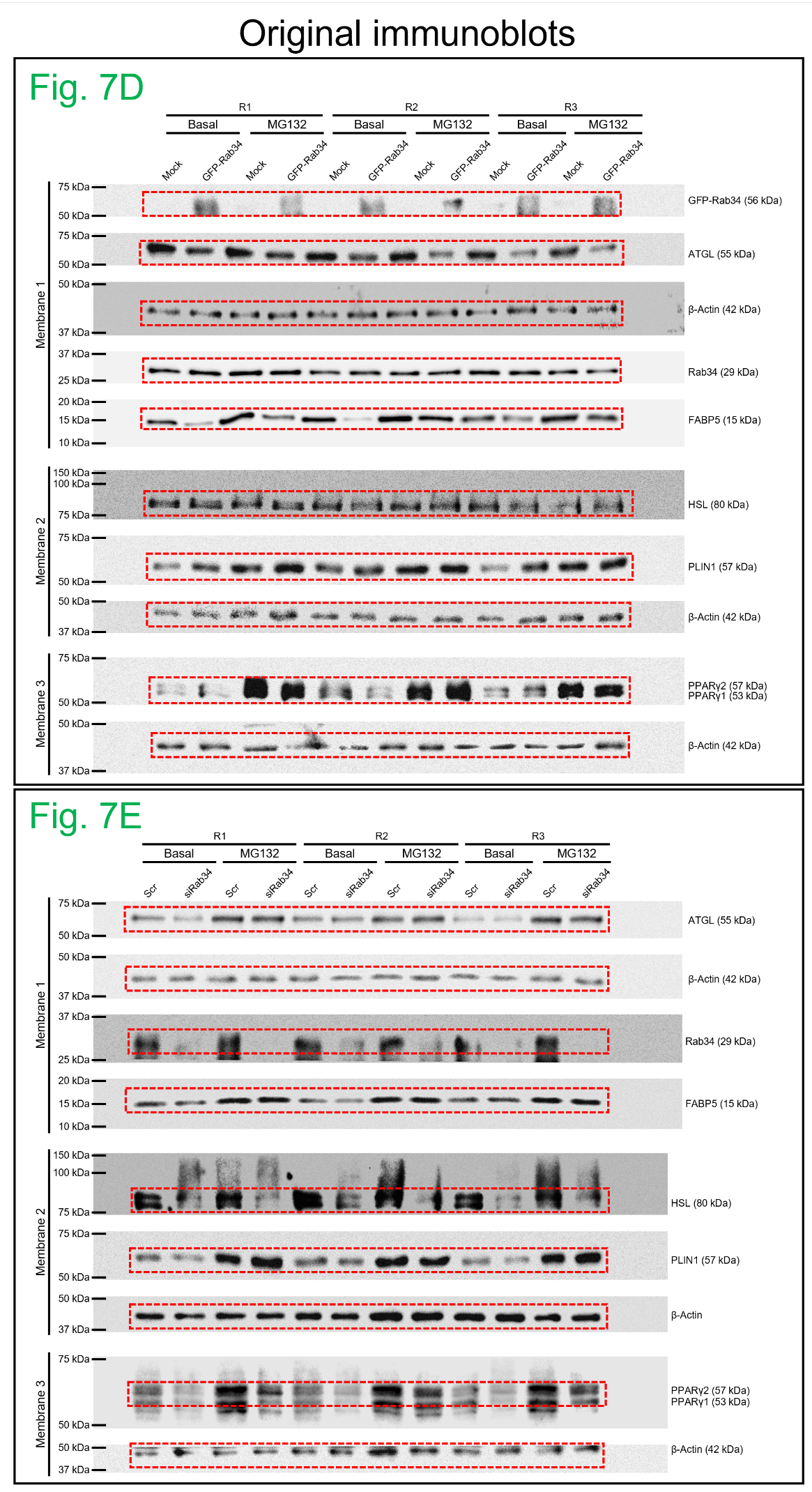


**Additional file 3: Fig. S12. Uncropped scans of all the western blots from Figure 7.** The red dashed boxes indicate the regions of interest shown in the corresponding figure.


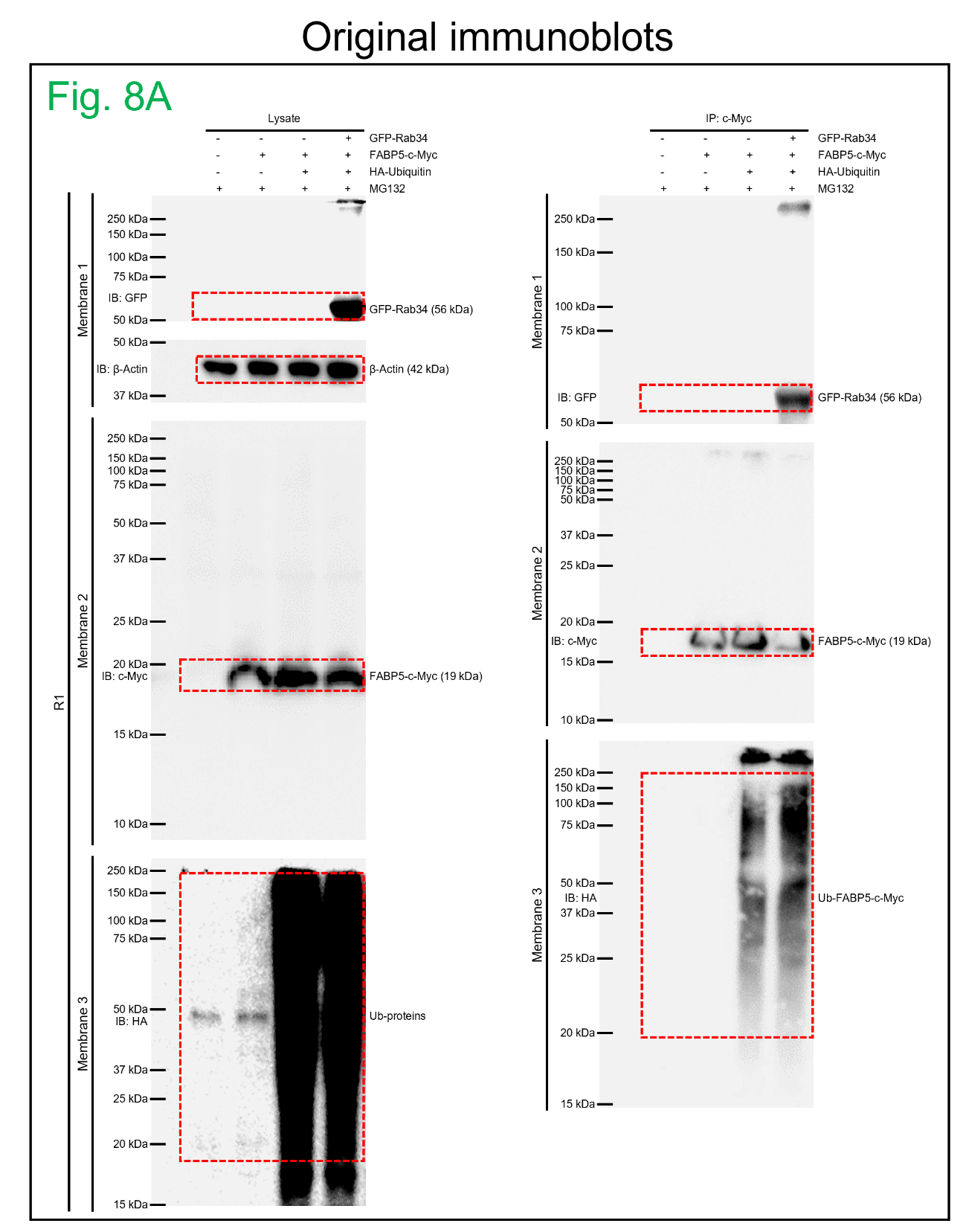


**Additional file 3: Fig. S13. Uncropped scans of western blots from Figure 8 (part 1 of 6).** The red dashed boxes indicate the regions of interest shown in the corresponding figure. Biological replicates in Figure 8A were run in three gels.


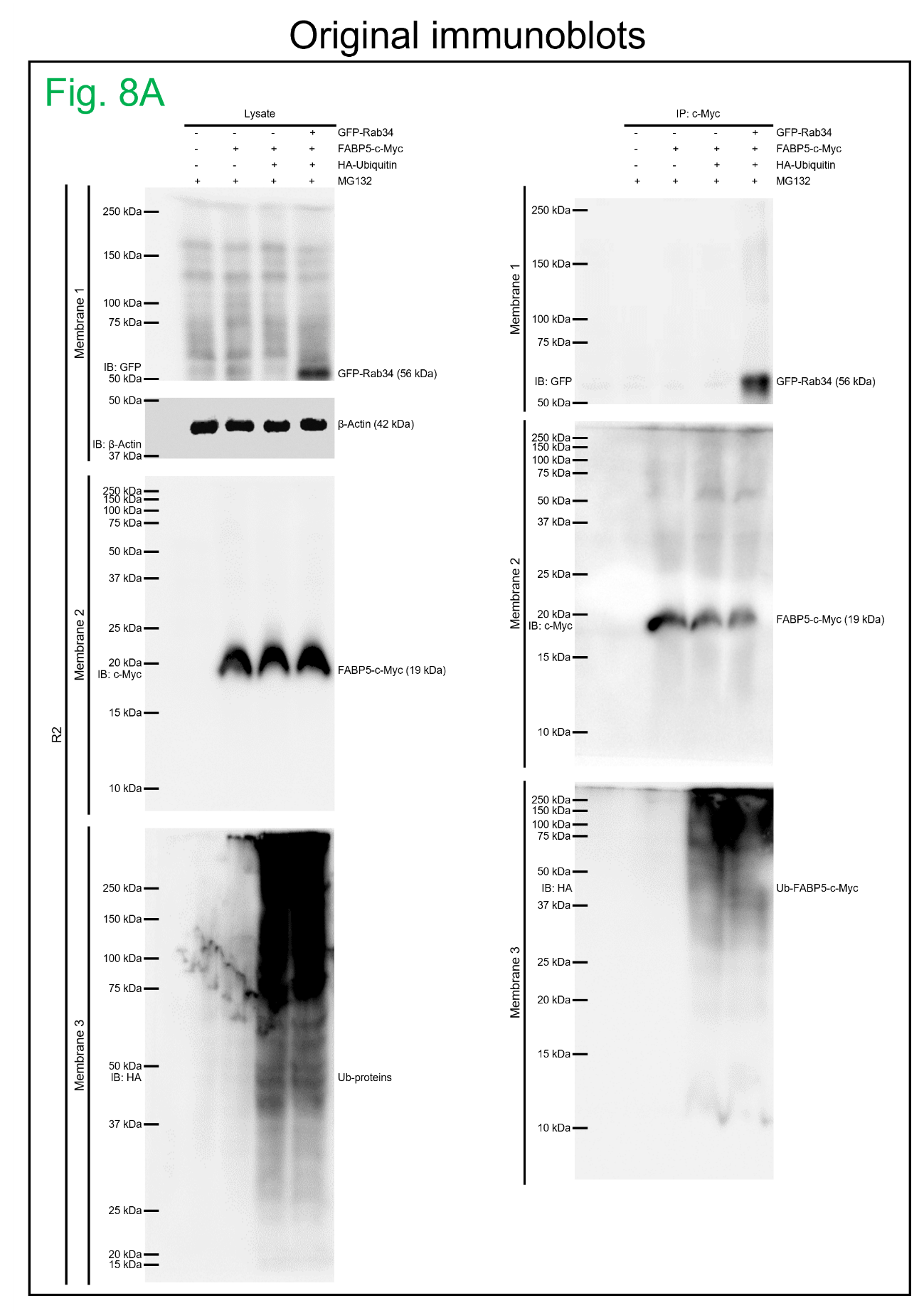


**Additional file 3: Fig. S14. Uncropped scans of western blots from Figure 8 (part 2 of 6).**


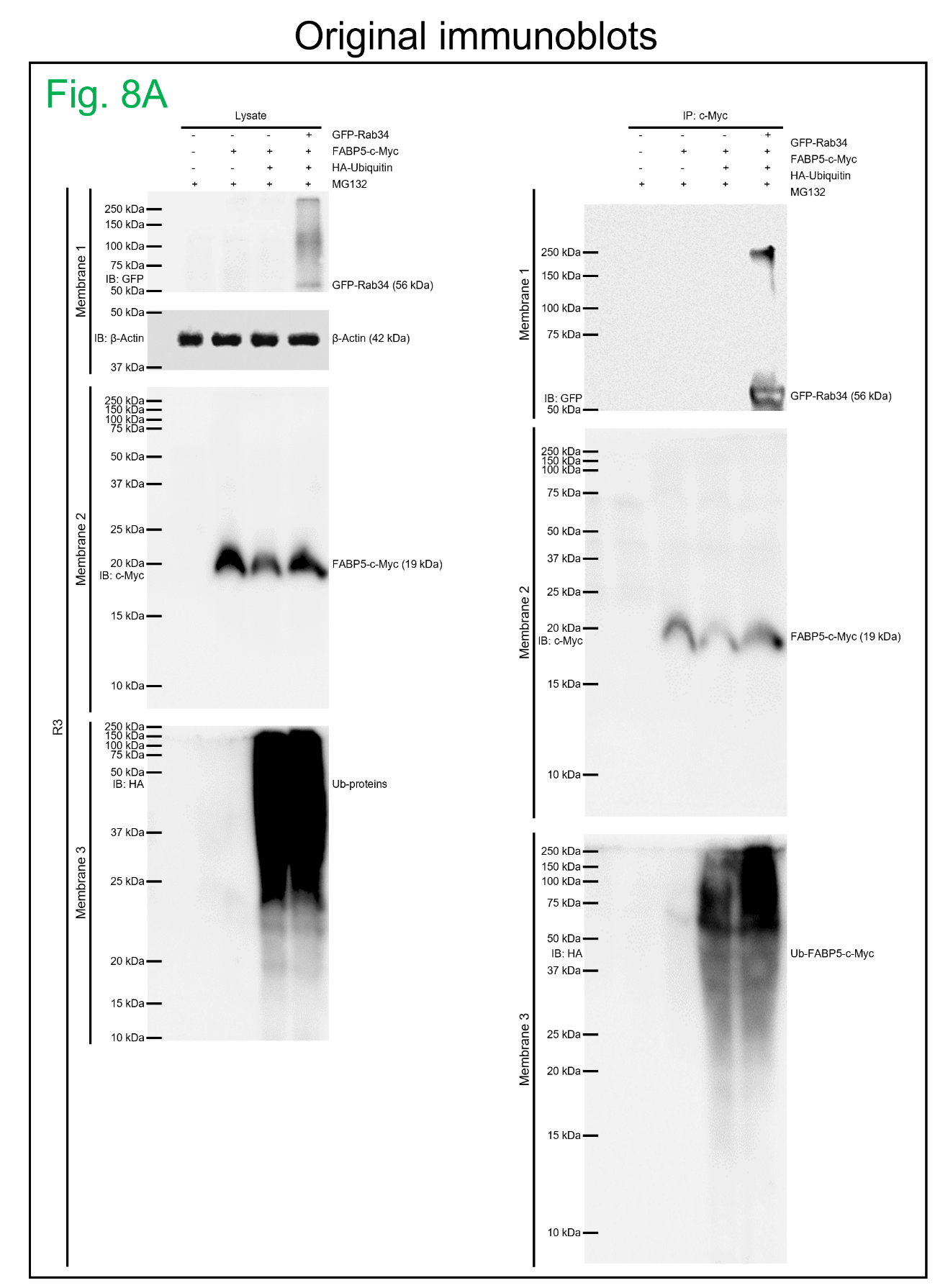


**Additional file 3: Fig. S15. Uncropped scans of western blots from Figure 8 (part 3 of 6).**


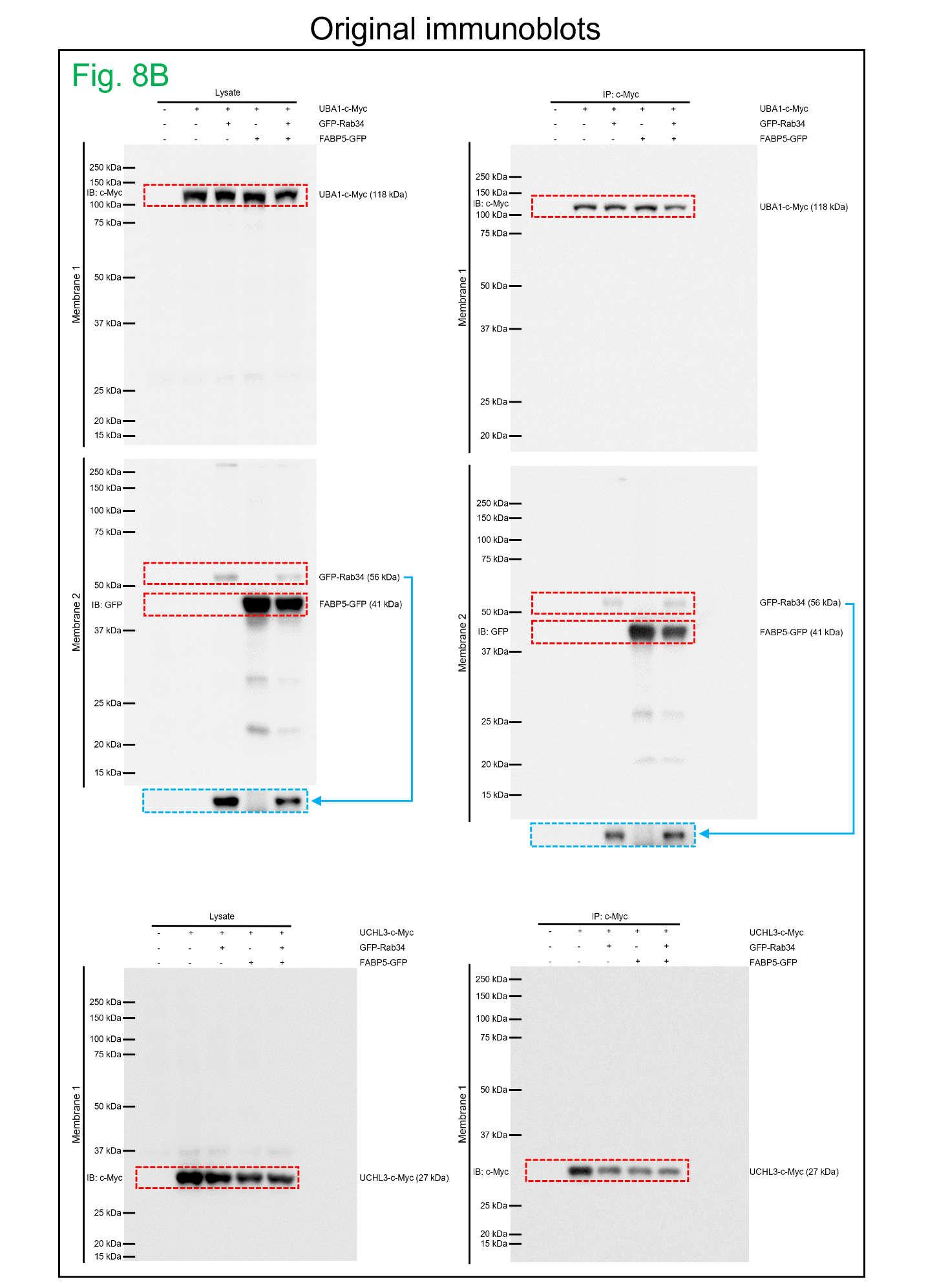


**Additional file 3: Fig. S16. Uncropped scans of western blots from Figure 8 (part 4 of 6).** The red dashed boxes indicate the regions of interest shown in the corresponding figure. The blue dashed boxes indicate the regions of interest shown in the corresponding figure when it was necessary to increase the exposure time to improve the band observation.


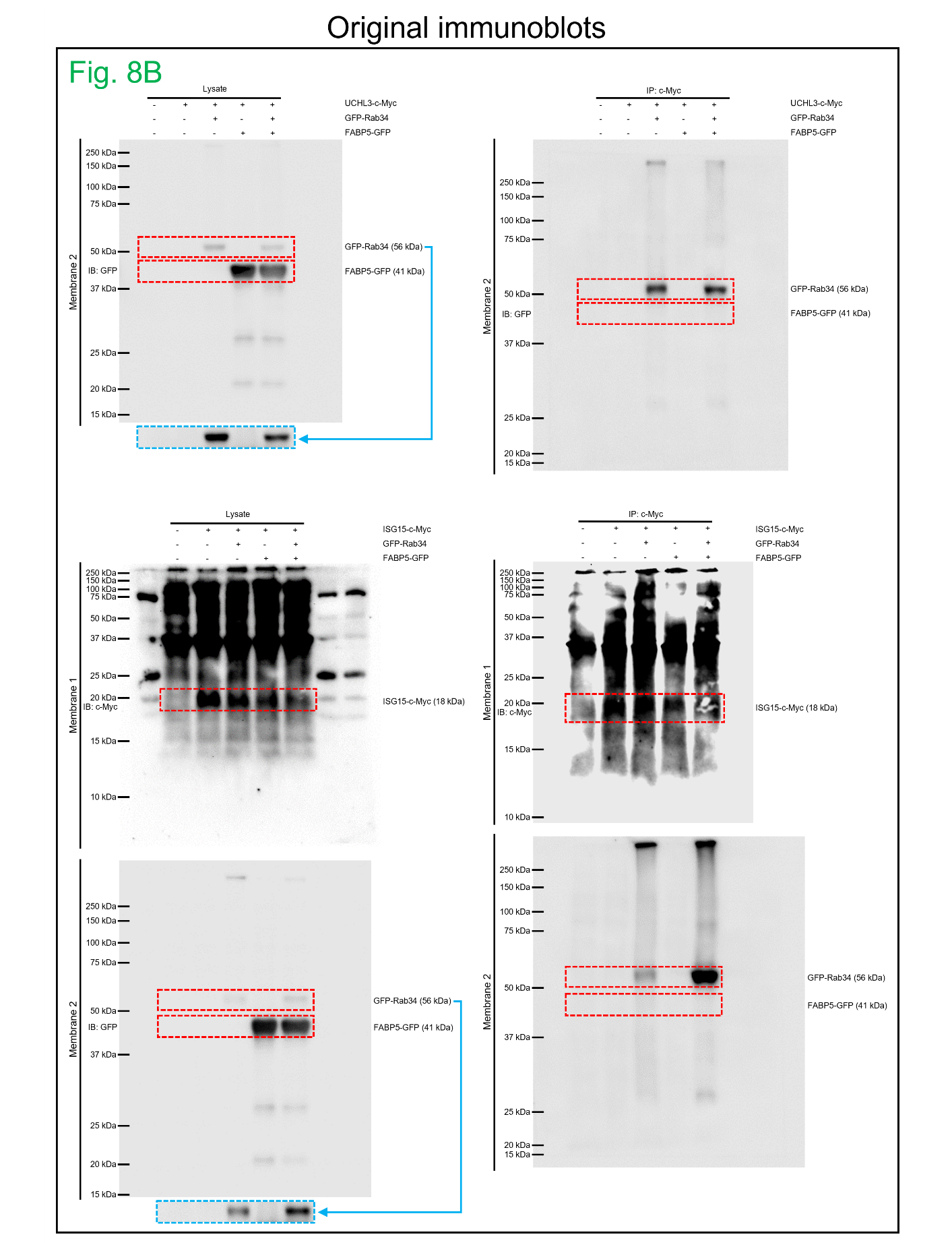


**Additional file 3: Fig. S17. Uncropped scans of western blots from Figure 8 (part 5 of 6).** The red dashed boxes indicate the regions of interest shown in the corresponding figure. The blue dashed boxes indicate the regions of interest shown in the corresponding figure when it was necessary to increase the exposure time to improve the band observation.


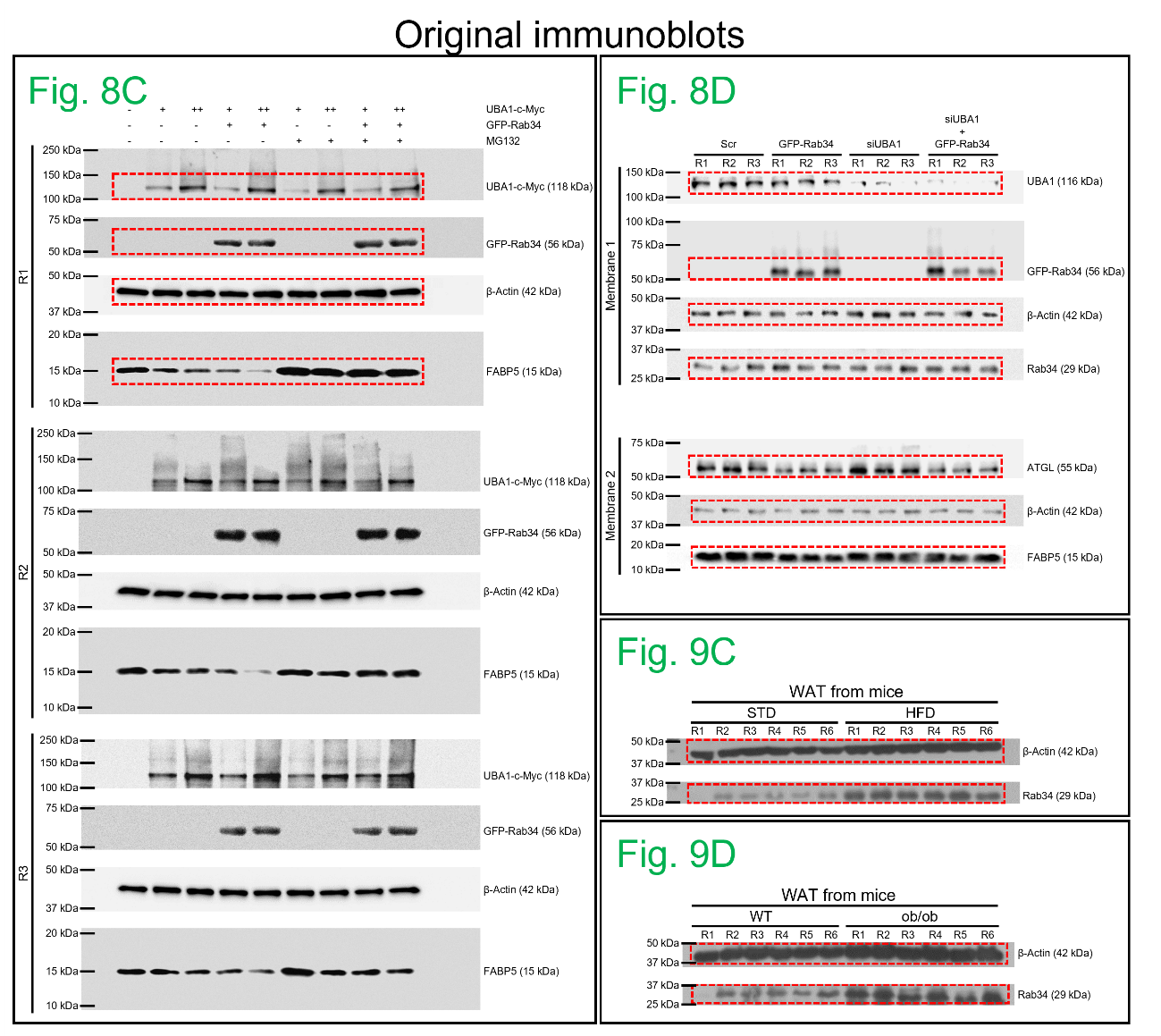


**Additional file 3: Fig. S18. Uncropped scans of western blots from Figures 8 (part 6 of 6) and 9.** The red dashed boxes indicate the regions of interest shown in the corresponding figure. Biological replicates in Figure 8C were run in three gels.


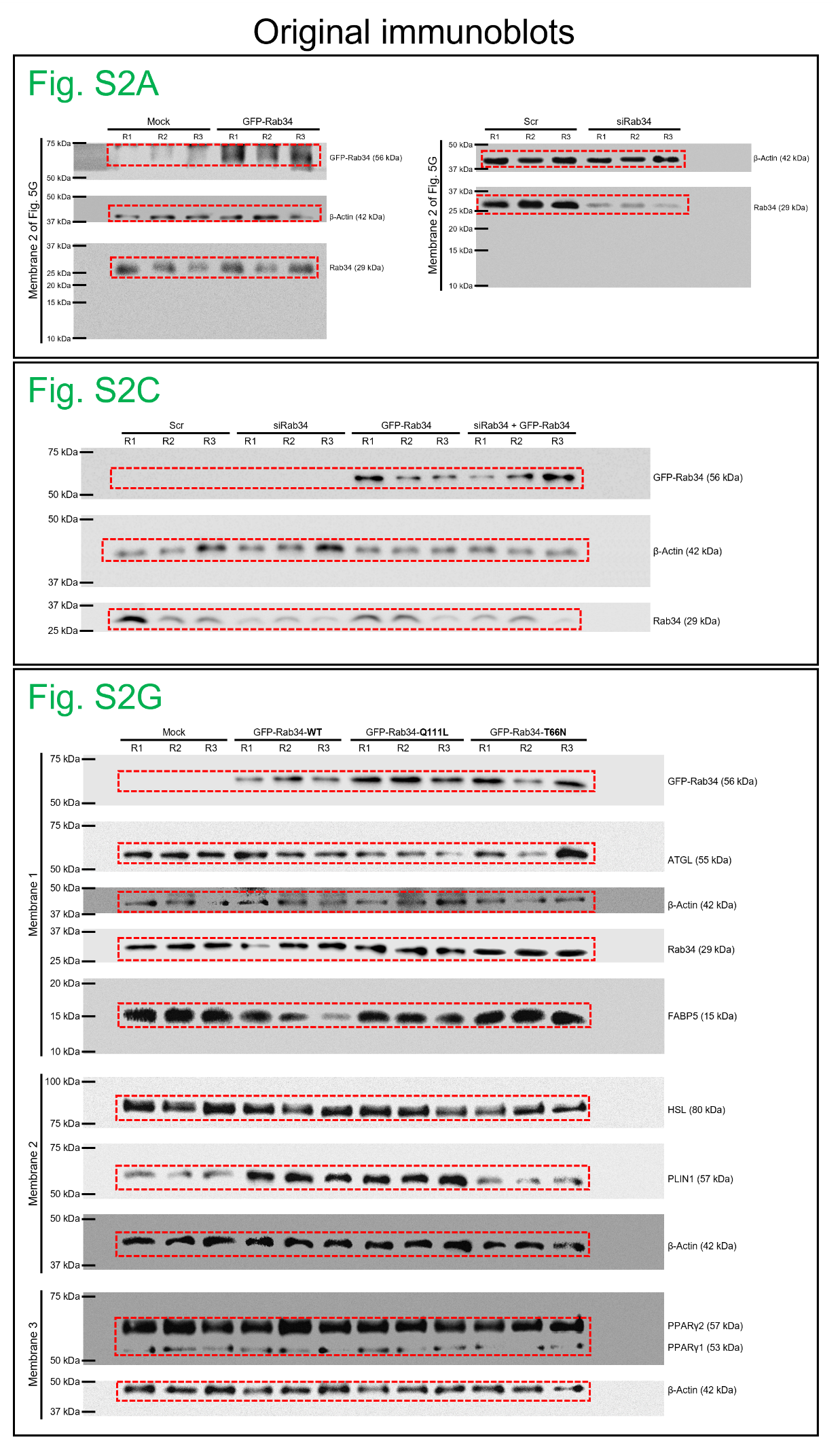


**Additional file 3: Fig. S19. Uncropped scans of western blots from Figures S2A, S2C and S2G.** The red dashed boxes indicate the regions of interest shown in the corresponding figure.


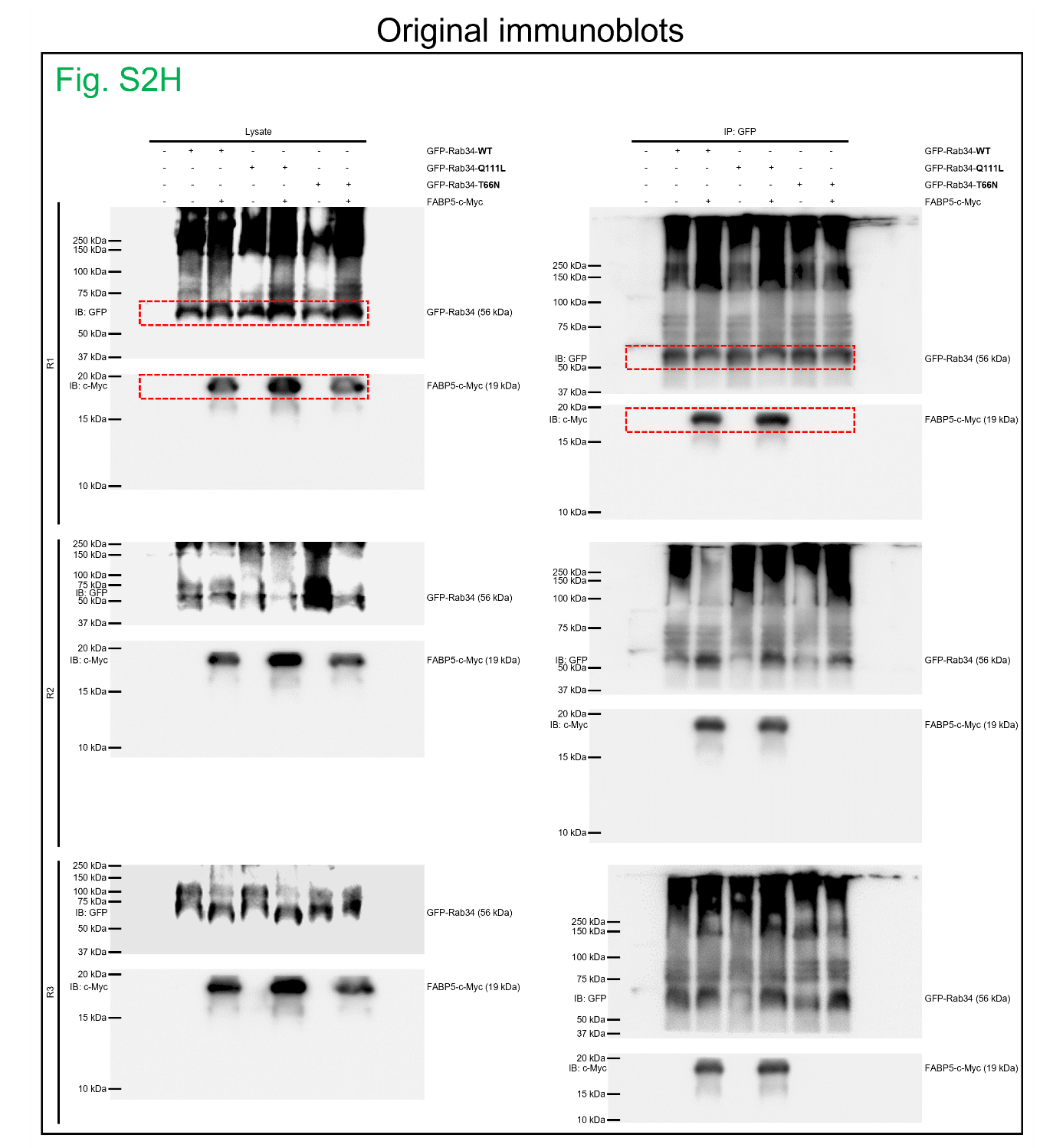


**Additional file 3: Fig. S20. Uncropped scans of western blots from Figure S2H.** The red dashed boxes indicate the regions of interest shown in the corresponding figure. Biological replicates were run in three gels.


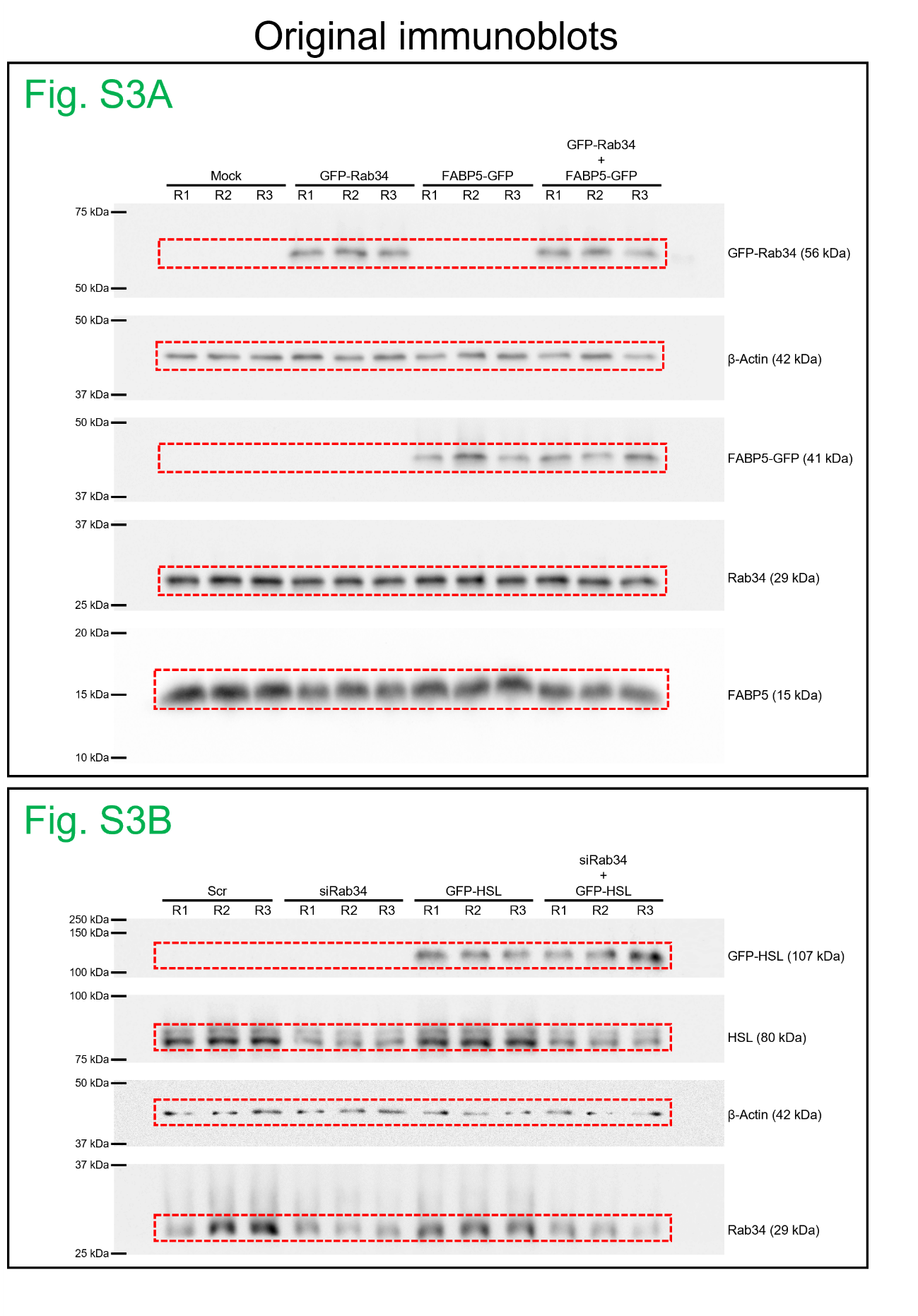


**Additional file 3: Fig. S21. Uncropped scans of western blots from Figures S3.** The red dashed boxes indicate the regions of interest shown in the corresponding figure.

**References**

[1] Fagerberg L, Hallstrom BM, Oksvold P, et al. Analysis of the human tissue-specific expression by genome-wide integration of transcriptomics and antibody-based proteomics. *Mol Cell Proteomics* 2014; 13: 397–406.

[2] Lee S, Zhang C, Kilicarslan M, et al. Integrated Network Analysis Reveals an Association between Plasma Mannose Levels and Insulin Resistance. *Cell Metab* 2016; 24: 172–184.
